# Identification of Differentially Expressed Circular RNAs as miRNA Sponges in Lung Adenocarcinoma

**DOI:** 10.1155/2021/5193913

**Published:** 2021-09-10

**Authors:** Yuechao Liu, Xin Wang, Lulu Bi, Hongbo Huo, Shi Yan, Yimeng Cui, Yaowen Cui, Ruixue Gu, Dexin Jia, Shuai Zhang, Li Cai, Xiaomei Li, Ying Xing

**Affiliations:** ^1^The Fourth Department of Medical Oncology, Harbin Medical University Cancer Hospital, 150 Haping Road, Harbin 150040, China; ^2^Department of Pathology, Harbin Medical University Cancer Hospital, 150 Haping Road, Harbin 150040, China

## Abstract

**Background:**

Circular RNAs (circRNAs) may function as the decoys for microRNAs (miRNAs) or proteins, the templates for translation, and the sources of pseudogene generation. The purpose of this study is to determine the diagnostic circRNAs, which are related to lung adenocarcinoma (LUAD), that adsorb miRNAs on the basis of the competing endogenous RNA (ceRNA) hypothesis.

**Methods:**

The differentially expressed circRNAs (DEcircRNAs) in LUAD were revealed by the microarray data (GSE101586 and GSE101684) that were obtained from the Gene Expression Omnibus (GEO) database. The miRNAs that were targeted by the DEcircRNAs were predicted with the CircInteractome, and the target mRNAs of the miRNAs were found by the miRDB and the TargetScan. The ceRNA network was built by the Cytoscape. The potential biological roles and the regulatory mechanisms of the circRNAs were investigated by the Gene Ontology (GO) enrichment analysis and the Kyoto Encyclopedia of Genes and Genomes (KEGG) analysis. The expression of the host genes of circRNAs was examined by the Ualcan. The survival analysis was performed by the Kaplan–Meier plotter.

**Results:**

In comparison with normal lung tissues, LUAD tissues contained 7 overlapping cancer-specific DEcircRNAs with 294 miRNA response elements (MREs). Among the 7 DEcircRNAs, 3 circRNAs (hsa_circ_0072088, hsa_circ_0003528, and hsa_circ_0008274) were upregulated and 4 circRNAs (hsa_circ_0003162, hsa_circ_0029426, hsa_circ_0049271, and hsa_circ_0043256) were downregulated. A circRNA-miRNA-mRNA regulatory network, which included 33 differentially expressed miRNAs (DEmiRNAs) and 2007 differentially expressed mRNAs (DEmRNAs), was constructed. These mRNAs were enriched in the biological function of cell-cell adhesion, response to hypoxia, and stem cell differentiation and were involved in the PI3K-Akt signaling, HIF-1 signaling, and cAMP signaling pathways.

**Conclusion:**

Our results indicated that 7 DEcircRNAs could have diagnostic value for LUAD. Additionally, the circRNAs-mediated ceRNA network might provide a novel perspective into unraveling the pathogenesis and progression of LUAD.

## 1. Introduction

Lung cancer is the second most commonly diagnosed cancer and the primary cause of cancer-related death worldwide [1]. In 2020, over 2.2 million new lung cancer cases and 1.8 million lung-cancer-related deaths were estimated [1]. Non-small-cell lung cancer (NSCLC) cases account for 85% of lung cancer, and the most common histological type of NSCLC is lung adenocarcinoma (LUAD) [2]. In spite of the improvement in chemotherapy and radiotherapy, the 5-year survival rate of LUAD remains below 20% [3]. Therefore, it is critical to clarify the underlying mechanisms and therapeutic targets of LUAD, which are beneficial for the diagnosis and prognostic evaluation [4].

An increasing amount of evidence has shown that noncoding RNAs such as long noncoding RNAs (lncRNAs), pseudogenic RNAs, and circular RNAs (circRNAs) may act as competing endogenous RNAs (ceRNAs) by competitively binding to several miRNAs [5, 6]. Compared with the traditional liner RNAs, circular RNAs (circRNAs) have a completely closed-loop structure [7]. CircRNAs have been identified to be the miRNA sponges involved in cancer development [8–10]. CircRNAs and mRNAs compete for binding to the limited targeting miRNAs via plentiful miRNA binding sites (MREs) regions to construct a ceRNA regulatory network [11]. When mRNAs competitively bind to miRNAs, the translation process is interrupted and the stability of miRNAs is compromised [12–14]. However, circRNAs remain stable and resist the degradation by RNA exonucleases [11].

Interestingly, ceRNA regulatory networks are dysregulated and are closely related to the occurrence and progression of different types of cancers, including LUAD [11, 15–17]. For example, a previous study revealed that circRNA-ENO1 played a crucial role in glycolysis and tumor progression of LUAD by promoting the expression of its host gene ENO1 [18]. In addition, circ_EPB41L2 played a protective role by repressing proliferation, migration, and invasion through regulating CDH4 and miR-211-5p in LUAD cells [19]. Moreover, in vivo studies indicated that the overexpression of circ_EPB41L2 inhibited tumor growth by regulating miR-211-5p and CDH4 [19]. Furthermore, bioinformatics analysis, which depends on the rise of high-throughput sequencing technology, has been widely used in the research on the etiology and the underlying mechanism of cancers. To explore the tumor markers with prognostic significance, researchers need to build a comprehensive ceRNA regulatory network through the in-depth analysis of public databases. Although many bioinformatics studies have been conducted on ceRNAs, novel circRNA molecules and ceRNA networks are worth further investigation [20–22]. Further research on the ceRNA network will help to explore novel diagnostic and treatment methods of LUAD.

In the present study, we collected the expression profiles of circRNAs (GSE101586 and GSE101684), miRNAs (GSE135918 and TCGA-LUAD), and mRNAs (TCGA-LUAD) of LUAD. Seven differentially expressed and cancer-specific circRNAs in LUAD were identified by bioinformatic analysis. A circRNA-miRNA-mRNA regulatory network was constructed. Furthermore, we performed functional enrichment analysis to reveal the potential biological function and mechanism of the circRNAs, which might provide new insights into the diagnosis and treatment of LUAD.

## 2. Methods

### 2.1. Research Process Design

**Figure 1:**
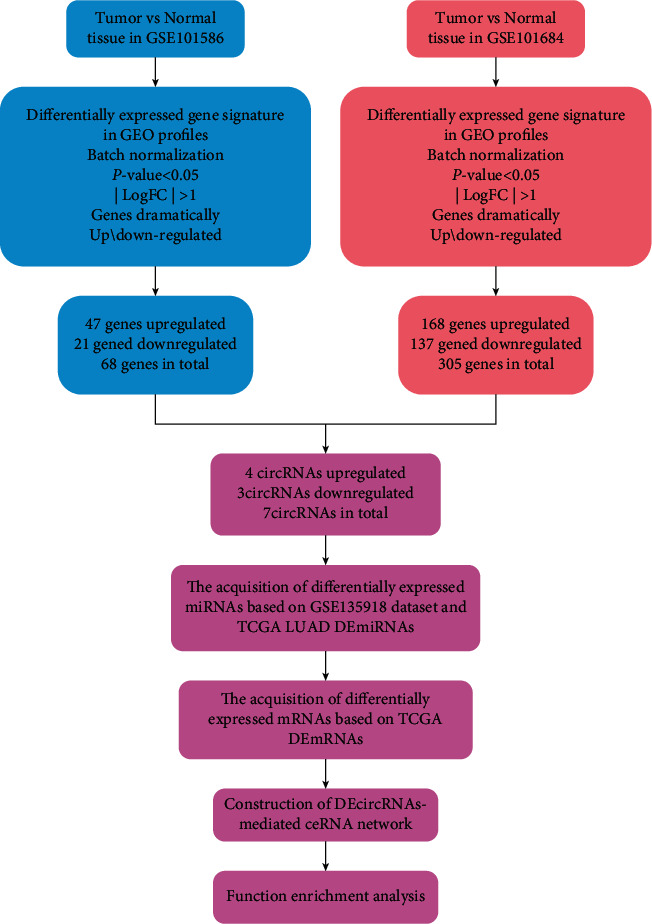
Flowchart for bioinformatics analysis of this study.

The experimental design and the specific implementation scheme of the study are shown in Figure 1.

### 2.2. Collection of the Data from Public Database

The Gene Expression Omnibus (GEO, https://www.ncbi.nlm.nih.gov/geo/) is a database widely applied in many fields. It involves the noncoding RNA analysis, the comparative genomic analysis, the proteomics analysis, the single nucleotide polymorphism genome analysis, and the DNA methylation status analysis. We searched for the microarray data in the GEO dataset by inputting “lung adenocarcinoma” and “circRNA” keywords. The inclusion and exclusion criteria were (1) human LUAD tissues and the adjacent normal human lung tissues, (2) microarray expression profiling of circRNA, and (3) data annotation platform and the original data matrix. Two datasets (GSE101586 and GSE101684) that satisfied the screening criteria were obtained and downloaded.

### 2.3. Data Processing

The base 2 logarithm (log_2_) transformation was used to transform the expression value of circRNAs and the R software was used to interpret the raw microarray data. For similar circRNAs in the expression matrix, we took the average expression value of the duplicates. The information on the probe annotation of the circRNAs was downloaded from GPL19978 and GPL21825 platform files. The detailed annotation of the probe, the sample, and the platform was obtained from GEO database. We standardized original expression data by the log_2_ transformed. We used an R package for the differentiation analysis of the microarray data and set the thresholds of |log_2_ (fold-change)| > 1 and *P* < 0.05 to determine the DEcircRNAs in each dataset. Then, we retained the DEcircRNAs and removed the circRNAs that were not differentially expressed.

### 2.4. Identification of DEcircRNAs

When multiple probes were mapped to a certain Agilent ID, the corresponding mean value was selected. The Perl scripting was used to convert the Agilent ID to gene names. To standardize the matrix information and analyze the differential gene expression among models, we used the limma package to normalize the raw microarray data. Whereafter, the fold-change and the *P* value were used to determine the differentially expressed genes in the 2 datasets, the screening criteria of which were |log_2_ (fold-change)| > 1 and *P* value <0.05. We used the CircBase database to obtain the host genes related to the circRNAs [23].

### 2.5. Prediction of CircRNA-MicroRNA-Target Gene Networks

The CircBase database (circbase.org/) and cancer-specific circRNAs database (CSCD, https://gb.whu.edu.cn/CSCD/) were used to predict MREs of the circRNAs that provided information on the circRNAs from multiple perspectives, including the location of circRNAs on chromosomes, the length variation of the circRNAs, and the interaction between the circRNAs and miRNAs. Specific miRNA targets were predicted based on the MicroRNA Target Prediction Database (miRDB, http://mirdb.org/) and the TargetScan (http://www.targetscan.org/vert_71/). The overlapping genes of the three databases were selected as host genes. The prognostic value of host genes were investigated by the Kaplan–Meier plotter.

### 2.6. GO Enrichment Analysis

In order to understand the potential function of the circRNAs, we performed the GO enrichment analysis of target genes by DAVID (https://david.ncifcrf.gov/home.jsp). GO analysis included the analysis of cellular components (CC), molecular functions (MF), and biological processes (BP). Each category of the GO analysis explained the different biological function of the genes. We used Sangerbox (http://www.sangerbox.com/) to visualize the results of the GO enrichment analysis.

## 3. Results

### 3.1. Identification of DEcircRNAs

**Figure 2:**
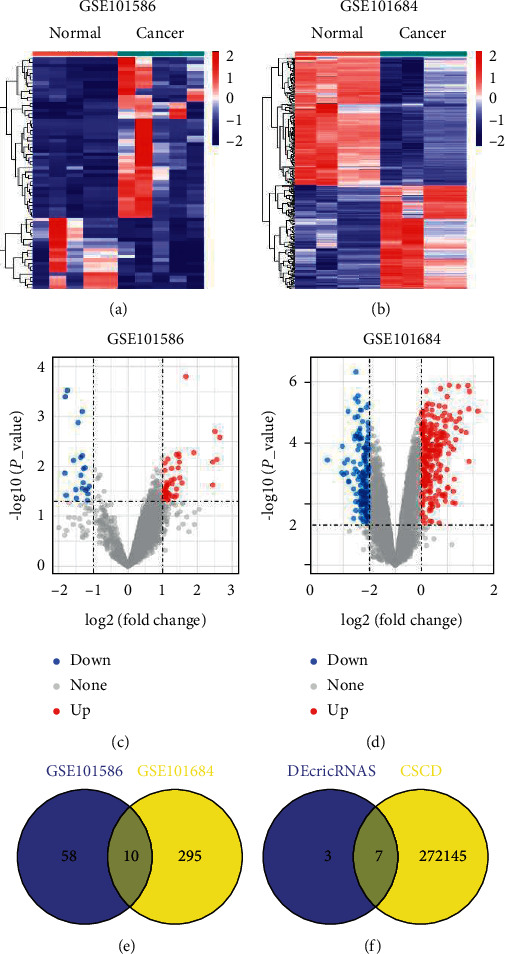
Identification of differentially expressed circRNAs in LUAD. (a, b) Hierarchical clustering heat map of the DEcircRNAs of LUAD samples compared with normal lung samples from GSE101586 and GSE101684, with absolute fold-changes >1 and *P* value < 0.05 as significant. (c, d) The volcano maps showed the number and distribution of the DEcircRNAs. The red dots represent the upregulated circRNAs and the blue dots indicate the downregulated circRNAs. (e) Venn diagram of the intersection of DEcircRNAs in GSE101586 and GSE101684 datasets. (f) Venn diagram of the intersection of differentially expressed and cancer-specific circRNAs based on Cancer-Specific CircRNA Database (CSCD, http://gb.whu.edu.cn/CSCD).

We downloaded and analyzed the GSE101586 and GSE101684 microarray data by the GEO2R tool to identify the DEcircRNAs between paired NSCLC tissues and adjacent nontumor tissues. The basic information on these 2 datasets is illustrated in Table 1. We obtained 68 DEcircRNAs that included 47 upregulated and 21 downregulated circRNAs on the basis of GSE101586 dataset and 305 DEcircRNAs that consisted of 168 upregulated and 137 downregulated circRNAs on the basis of the GSE101684 dataset (Figures 2(a) and 2(b), *P* value < 0.05 and absolute value of fold-change >1). We provided two volcano plots to visualize the DEcircRNAs (Figures 2(c) and 2(d)). The Venn diagram shown in Figure 2(e) displays the 10 overlapping DEcircRNAs between the two datasets.

The cancer-specific circRNA database (CSCD, http://gb.whu.edu.cn/CSCD) is a useful circRNA database to deduce whether particular circRNAs are cancer-specific [24]. The 7 differentially expressed and cancer-specific circRNAs in LUAD are presented in Figure 2(f).

### 3.2. Characterization of DEcircRNAs

**Figure 3:**
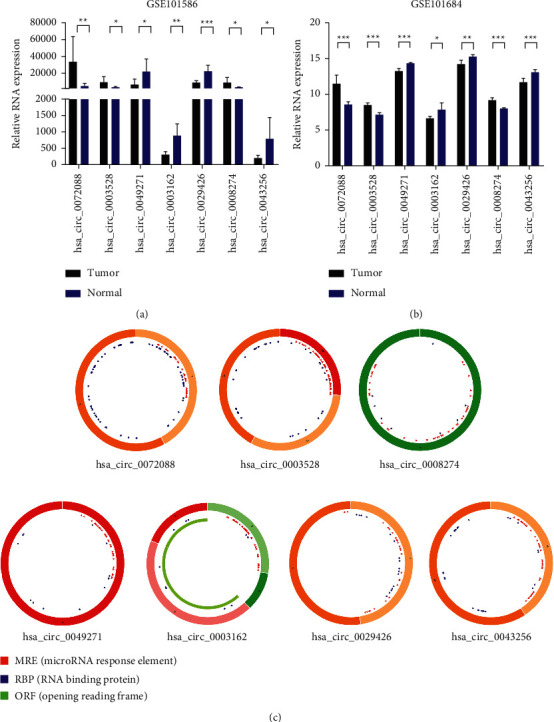
Characterization of DEcircRNAs. (a, b) The expression levels of DEcircRNAs in human LUAD tissues and normal lung samples were analyzed based on GSE101586 and GSE101684 datasets. The differences were compared by paired *t*-test. Mean ± SEM, *n* = 3, ^*∗*^*P* < 0.05; ^*∗∗*^*P* < 0.01; ^*∗∗∗*^*P* < 0.001. (c) Structural patterns of the 7 circRNAs: hsa_circ_0072088, hsa_circ_0003528, hsa_circ_0008274, hsa_circ_0049271, hsa_circ_0003162, hsa_circ_0029426, and hsa_circ_0043256. The green part represents the open reading frame (ORF) of circRNAs. The blue part is the place where circRNA binds to the proteins. The red small triangle represents the binding position of the circRNA to the miRNA.

As shown in Figures 3(a) and 3(b), 3 circRNAs (hsa_circ_0072088, hsa_circ_0003528, and hsa_circ_0008274) were upregulated and 4 circRNAs (hsa_circ_0003162, hsa_circ_0029426, hsa_circ_0049271, and hsa_circ_0043256) were downregulated. In this study, we used the CircBase database to determine the location, genomic length, strand, and gene symbol of the 7 DEcircRNAs. The basic characteristics of the DEcircRNAs are listed in Table 2.

Moreover, the structural patterns of the 7 circRNAs from the CSCD database are shown in Figure 3(c). 294 MREs were predicted for the cancer-specific DEcircRNA candidates. Specifically, we found that hsa_circ_0072088 harbored 32 MREs, hsa_circ_0003528 harbored 57 MREs, hsa_circ_0008274 harbored 51 MREs, hsa_circ_0049271 harbored 38 MREs, hsa_circ_0003162 harbored 42 MREs, hsa_circ_0029426 harbored 28 MREs, and hsa_circ_0043256 harbored 48 MREs. These findings suggested that the DEcircRNAs were potential miRNA sponges (Figure 3(c)).

The DEcircRNAs were the partial fragments transcribed by the host genes. Next, we examined the expression and diagnostic and prognostic significance of the host genes of these 7 cancer-specific DEcircRNAs. The host genes of hsa_circ_0049271, hsa_circ_0029426, and hsa_circ_0072088 had diagnostic and prognostic significance (Supplementary Figure S1).

### 3.3. The Determination of Differentially Expressed miRNAs and Differentially Expressed mRNAs

**Figure 4:**
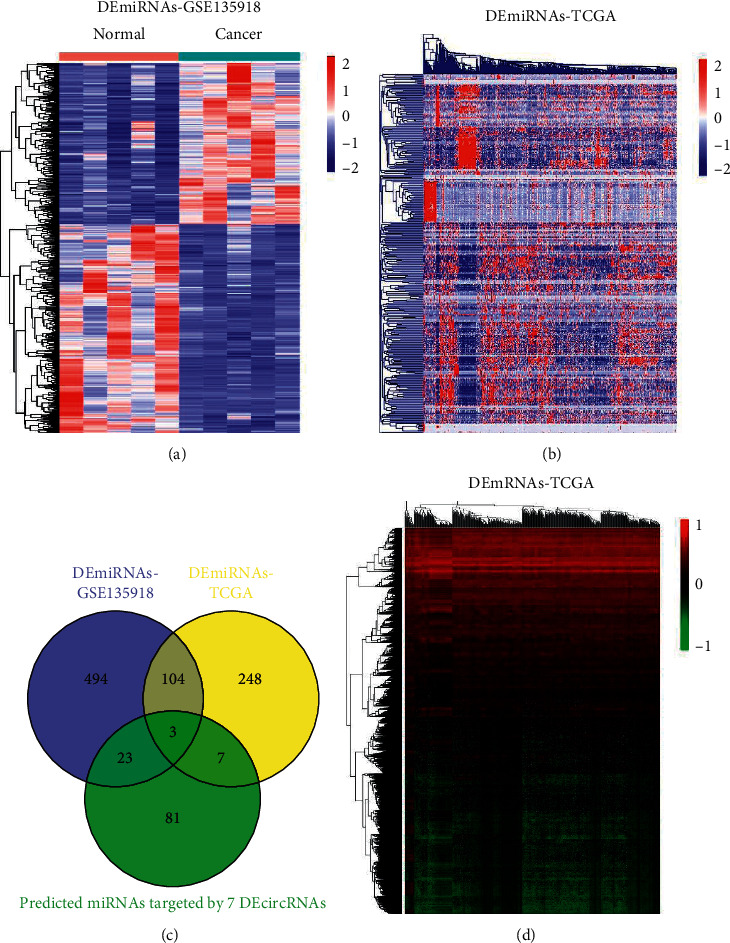
Identification of differentially expressed miRNAs and mRNAs. (a, b) Hierarchical clustering heat map of the DEmiRNAs of LUAD samples compared with normal lung samples from GSE135918 dataset (a) and TCGA-LUAD dataset (b). (c) Venn diagram of the union of DEmiRNAs in GSE135918 dataset and TCGA-LUAD dataset. (d) Hierarchical clustering heat map of the DEmRNAs of LUAD samples compared with normal lung samples based on TCGA-LUAD dataset.

On the basis of the GSE135918 dataset, we determined out 624 differentially expressed miRNAs (DEmiRNAs) in LUAD. CircInteractome database was used to predict the miRNAs targeted by the 7 DEcircRNAs, and 114 miRNAs were found.  Figure 4(a) illustrated GSE135918 dataset contained 2693 miRNAs, with 624 DEmiRNAs. On the other hand, the TCGA-LUAD dataset contained 2197 miRNAs and 362 DEmiRNAs (Figure 4(b)). 33 DEmiRNAs that were related to the DEcircRNAs were found by Venn analysis (Figure 4(c)). Then, by the miRDB and TargetScan databases, we searched for the target mRNAs of the DEmiRNAs targeted by the 7 DEcircRNAs. A total of 7622 target genes were bound to the 33 DEmiRNAs. On the basis of the TCGA-LUAD database, 2007 out of 7622 mRNAs were found to be differentially expressed (Figure 4(d)).

### 3.4. Construction of a ceRNA Network

**Figure 5:**
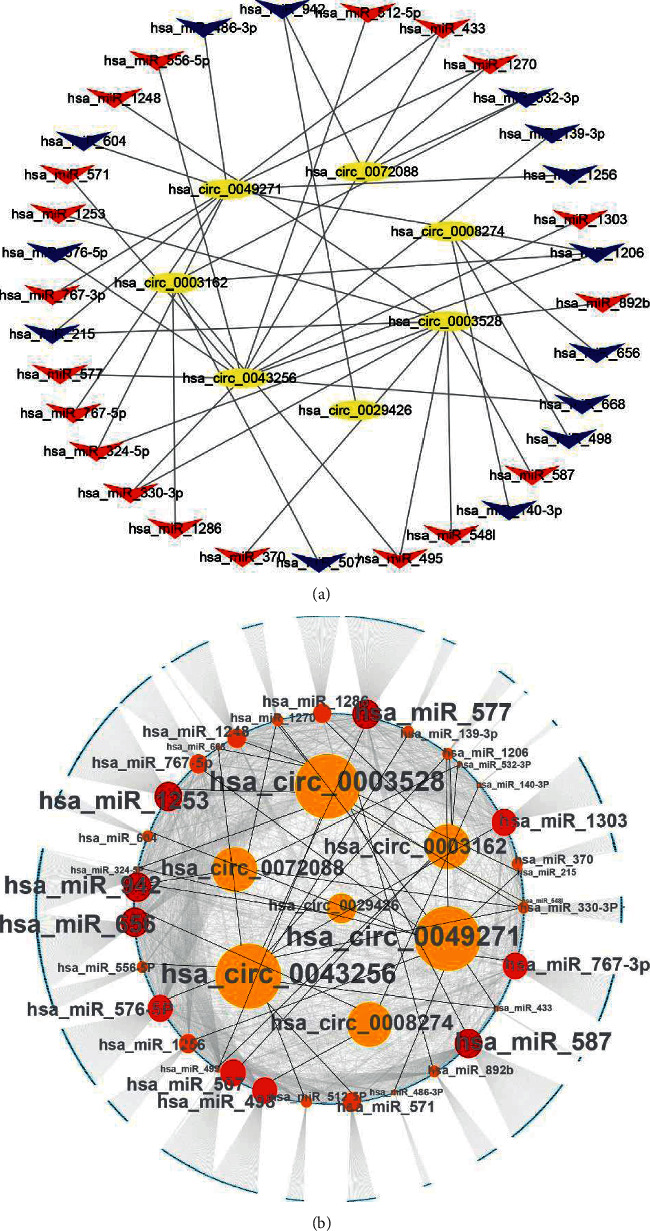
Construction of circRNA-miRNA-mRNA networks. (a) The relationship between 7 DEcircRNAs and their interacting DEmiRNAs (*n* = 33). Red, upregulation. Blue, downregulation. (b) circRNA-miRNA-mRNA regulatory networks using their interactions with Cytoscape software (version 3.8.2). Sizes of circles represent the weight of connection of circRNAs and miRNAs. The black lines connect circRNAs with miRNAs. The grey lines connect miRNAs with mRNAs.

In previous sections, we obtained 7 DEcircRNAs, 33 DEmiRNAs, and 2007 DEmRNAs and elucidated the interaction among the DEcircRNAs, the DEmiRNAs, and the DEmRNAs. Next, we used Cytoscape 3.8.2 to visualize the circRNA-miRNA-mRNA regulatory network (Figures 5(a) and 5(b) and Supplementary Table 1).

### 3.5. Functional Enrichment Analysis

**Figure 6:**
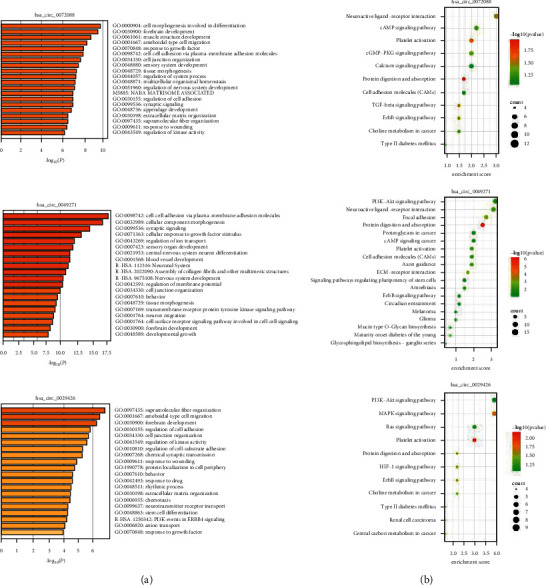
Functional enrichment analysis of targeted mRNA. (a) GO enrichment analysis and (b) KEGG pathway enrichment analysis of DEmRNAs of hsa_circ_0072088, hsa_circ_0049271, and hsa_circ_0029426.

The potential biological roles and regulatory mechanisms of circRNAs were investigated using GO enrichment analysis and Kyoto Encyclopedia of Genes and Genomes (KEGG) analysis. The mRNAs were enriched in the biological function of cell-cell adhesion, response to hypoxia, and stem cell differentiation (Figure 6(a) and Supplementary Figure S2(a)). Moreover, the mRNAs were involved in the PI3K-Akt signaling, HIF-1 signaling, and cAMP signaling pathways (Figure 6(b) and Supplementary Figure S2(b)).

## 4. Discussion

In view of the high mortality and the extremely low survival rate of LUAD, the determination of the specific biomarkers for the diagnosis and the treatment of LUAD remains critical [4, 25]. Previous studies found that noncoding RNAs, especially circRNAs, contribute to the malignant progression (the metastasis, proliferation, and tumor growth) of multiple types of cancer [26–30]. CircRNAs have high stability due to the unique covalent closed-loop structure that allows the circRNAs to resist the RNase R degradation [31]. Many studies reported that the ceRNA regulatory networks that were constructed by circRNAs contained substantial diagnosis and prognostic value. In our study, we comprehensively analyzed the ceRNA network for reliable diagnostic markers. Similarly, Li et al. [32] selected the combination of GSE101586, GSE101684, and GSE112214 and identified the DEcircRNAs in the patients with early-stage NSCLC. By utilizing the CSCD, we identified that the differentially expressed and cancer-specific cirRNAs in LUAD were potential miRNA sponges for the first time.

Subsequently, the function enrichment analysis of mRNAs suggested the relevant biological function and pathways for LUAD. Our study revealed that the target mRNAs selected from the ceRNA network were enriched in the biological function of cell-cell adhesion, response to hypoxia, and stem cell differentiation. Moreover, the target mRNAs were involved in the PI3K-Akt signaling, HIF-1 signaling, and cAMP signaling pathways. Then, we applied bioinformatics analysis to explore the structure of circRNAs and used CircBase and CSCD database to figure out the location, genomic length, strand, and gene symbol of the circRNAs. The cancer-specific circRNAs that exhibited the MRE, the RNA binding protein (RBP), and the open reading frame (ORF) were determined to be ceRNAs. MREs are observed on lncRNAs, circRNAs, and protein-coding mRNAs, which can compete for the miRNAs, form a ceRNA regulatory network, and absorb RBPs [12]. A large amount of evidence identified that noncoding RNAs played a role in regulating gene expression at both the transcriptional and posttranscriptional level by the physical interaction with RBPs or other noncoding RNAs [33]. On the other hand, RBPs contribute to circRNA biogenesis of exons circularization through narrowing the distance between the donor site and the receptor site by binding to the introns on the flank regions [34]. Additionally, ORF advances the translation of engineered circRNAs [35].

Out of the 7 DEcircRNAs identified in this study, 3 DEcircRNAs had been reported in previous studies [36, 37]. Hsa_circ_0072088 was identified as a ceRNA of miR-377-5p via upregulating NOVA2 to expedite the proliferation and metastasis of NSCLC [36]. Moreover, hsa_circ_0008274 and hsa_circ_0043256 that were derived from the ceRNA network were reported to be involved in the cancer progression [37]. Bioinformatics-related analysis suggested the significance of the understanding of the potential mechanisms of circRNAs-miRNAs-target gene network [10, 38–40].

Although we have described the significance of the ceRNA network in the clinical diagnosis of LUAD, the investigation of the prognostic value and clinical parameters is urgently needed to validate our findings in the samples from the patients with clinical LUAD. Moreover, molecular experiments need to be conducted to validate the biological function and the molecular mechanisms of the identified genes in LUAD cell lines.

## 5. Conclusions

In summary, by bioinformatics analysis, we identified 3 significantly upregulated circRNAs and 4 significantly downregulated circRNAs from public databases, which indicated the potential of the DEcircRNAs for the noninvasive biomarkers for the LUAD diagnosis. In future study, the biological function and the molecular regulatory mechanisms of the DEcircRNAs need to be experimentally validated.

## Figures and Tables

**Table 1:** The information of GSE101586 and GSE101684 datasets obtained from the GEO database.

Reference	Tissue	GEO	Platform	Normal	Tumor	Upregulated gene	Downregulated gene
Qiu M. et al., 2017	LUAD	GSE101586	GPL19978	5	5	47	21
Xu M. et al., 2019	LUAD	GSE101684	GPL21825	4	4	168	137

**Table 2:** The information of the 7 cancer-specific DEcircRNAs.

Genes total	Up-/downregulated	Position	Genomic length	Gene symbol	Strand
hsa_circ_0029426	Down	chr12 : 131357380-131357465	85	RAN	+
hsa_circ_0043256	Down	chr17 : 35604934-35609962	5028	ACACA	−
hsa_circ_0049271	Down	chr19 : 10610070-10610756	686	KEAP1	−
hsa_circ_0003162	Down	chr7 : 33185853-33217203	31350	BBS9	+
hsa_circ_0003528	Up	chr5 : 134032815-134044578	11763	SEC24A	+
hsa_circ_0072088	Up	chr5 : 32379220-32388780	9560	ZFR	−
hsa_circ_0008274	Up	chr13 : 96485180-96489456	4276	UGGT2	−

## Data Availability

All data generated or analyzed during this study are included in this published article and its supplementary information files.

## Supplementary Materials

Supplementary Materials Supplementary Figure S1: the diagnostic and prognostic significance of the host gene. (a) The expression of the host genes of hsa_circ_0049271, hsa_circ_0029426, and hsa_circ_0072088 in The Cancer Genome Atlas (TCGA)-LUAD dataset. (b) Survival analysis for the host genes of the three circRNAs in LUAD patients was performed using the Kaplan–Meier plotter. ^*∗*^*P* < 0.05; ^*∗∗*^*P* < 0.01; ^*∗∗∗*^*P* < 0.001. Supplementary Figure S2: (a) GO enrichment analysis and (b) KEGG pathway enrichment analysis of DEmRNAs of hsa_circ_0003162, hsa_circ_0003528, hsa_circ_0008274, and hsa_circ_0043256. Supplementary Table 1: the construction of a circRNA-miRNA-mRNA regulatory network.
