# Supplementary material for: Identification of Differentially Expressed Circular RNAs as miRNA Sponges in Lung Adenocarcinoma
Source: J Oncol. 2021 Sep 10;2021:5193913. doi: 10.1155/2021/5193913 (PMC8448594; doi:10.1155/2021/5193913)
Supplement: Supplementary Materials — Supplementary Figure S1: the diagnostic and prognostic significance of the host gene. (a) The expression of the host genes of hsa_circ_0049271, hsa_circ_0029426, and hsa_circ_0072088 in The Cancer Genome Atlas (TCGA)-LUAD dataset. (b) Survival analysis for the host genes of the three circRNAs in LUAD patients was performed using the Kaplan–Meier plotter. ∗P < 0.05; ∗∗P < 0.01; ∗∗∗P < 0.001. Supplementary Figure S2: (a) GO enrichment analysis and (b) KEGG pathway enrichment analysis of DEmRNAs of hsa_circ_0003162, hsa_circ_0003528, hsa_circ_0008274, and hsa_circ_0043256. Supplementary Table 1: the construction of a circRNA-miRNA-mRNA regulatory network. [file 5193913.f1.zip › 5193913.f1/Supplementary Table 1.pdf]

Supplementary Table 1. The construction of a circRNA-miRNA-mRNA regulatory network.

DEcircRNA	DEmiRNA	DEmRNA
hsa_circ_0003162	hsa_miR_507	RBMS3
hsa_circ_0003162	hsa_miR_507	SCN3A
hsa_circ_0003162	hsa_miR_507	PRKCE
hsa_circ_0003162	hsa_miR_507	MDGA2
hsa_circ_0003162	hsa_miR_507	LPGAT1
hsa_circ_0003162	hsa_miR_507	KCNN3
hsa_circ_0003162	hsa_miR_507	CBX4
hsa_circ_0003162	hsa_miR_507	TET1
hsa_circ_0003162	hsa_miR_507	SCN1A
hsa_circ_0003162	hsa_miR_507	RIMS2
hsa_circ_0003162	hsa_miR_507	UNC13A
hsa_circ_0003162	hsa_miR_507	GRP
hsa_circ_0003162	hsa_miR_507	ISX
hsa_circ_0003162	hsa_miR_507	AMPH
hsa_circ_0003162	hsa_miR_507	FST
hsa_circ_0003162	hsa_miR_507	GRIK2
hsa_circ_0003162	hsa_miR_507	TRPM1
hsa_circ_0003162	hsa_miR_507	OLIG3
hsa_circ_0003162	hsa_miR_507	CTNNA2
hsa_circ_0003162	hsa_miR_507	GRIP1
hsa_circ_0003162	hsa_miR_507	NELL2
hsa_circ_0003162	hsa_miR_507	DAB2IP
hsa_circ_0003162	hsa_miR_507	DKK2
hsa_circ_0003162	hsa_miR_507	SECISBP2L
hsa_circ_0003162	hsa_miR_507	PAX6
hsa_circ_0003162	hsa_miR_507	CRTAM
hsa_circ_0003162	hsa_miR_507	CNKSR2
hsa_circ_0003162	hsa_miR_507	FAM43A
hsa_circ_0003162	hsa_miR_507	MEIS1
hsa_circ_0003162	hsa_miR_507	KDELR2
hsa_circ_0003162	hsa_miR_507	TRIP13
hsa_circ_0003162	hsa_miR_507	SYT14
hsa_circ_0003162	hsa_miR_507	LHX8
hsa_circ_0003162	hsa_miR_507	MLLT11
hsa_circ_0003162	hsa_miR_507	SEMA6D
hsa_circ_0003162	hsa_miR_507	PTPRM
hsa_circ_0003162	hsa_miR_507	LRRN1
hsa_circ_0003162	hsa_miR_507	PTPRD
hsa_circ_0003162	hsa_miR_507	FLRT3
hsa_circ_0003162	hsa_miR_507	PF4
hsa_circ_0003162	hsa_miR_507	ATP1B1
hsa_circ_0003162	hsa_miR_507	E2F7
hsa_circ_0003162	hsa_miR_507	LCN10
hsa_circ_0003162	hsa_miR_507	GLRA3
hsa_circ_0003162	hsa_miR_507	DTL
hsa_circ_0003162	hsa_miR_507	PABPC1
hsa_circ_0003162	hsa_miR_507	CHODL
hsa_circ_0003162	hsa_miR_507	LCOR
hsa_circ_0003162	hsa_miR_507	AVL9
hsa_circ_0003162	hsa_miR_507	PHKA1
hsa_circ_0003162	hsa_miR_507	EPHB1
hsa_circ_0003162	hsa_miR_507	JAKMIP3
hsa_circ_0003162	hsa_miR_507	KCNH7
hsa_circ_0003162	hsa_miR_507	ZIC1
hsa_circ_0003162	hsa_miR_507	TMTC4
hsa_circ_0003162	hsa_miR_507	DAZL

hsa_circ_0003162	hsa_miR_507	EMP1
hsa_circ_0003162	hsa_miR_507	CCND2
hsa_circ_0003162	hsa_miR_507	GNAQ
hsa_circ_0003162	hsa_miR_507	NECAB1
hsa_circ_0003162	hsa_miR_507	SIM1
hsa_circ_0003162	hsa_miR_507	EPHA8
hsa_circ_0003162	hsa_miR_507	DLK1
hsa_circ_0003162	hsa_miR_507	SPTSSB
hsa_circ_0003162	hsa_miR_507	RUNX2
hsa_circ_0003162	hsa_miR_507	PKHD1
hsa_circ_0003162	hsa_miR_507	FGFBP2
hsa_circ_0003162	hsa_miR_507	GABRA4
hsa_circ_0003162	hsa_miR_507	CACNA1E
hsa_circ_0003162	hsa_miR_507	NKX2-2
hsa_circ_0003162	hsa_miR_507	FBN2
hsa_circ_0003162	hsa_miR_507	SIX3
hsa_circ_0003162	hsa_miR_507	VSX1
hsa_circ_0003162	hsa_miR_507	AK5
hsa_circ_0003162	hsa_miR_507	MCIDAS
hsa_circ_0003162	hsa_miR_507	ZNF681
hsa_circ_0003162	hsa_miR_507	SYNM
hsa_circ_0003162	hsa_miR_507	SATB2
hsa_circ_0003162	hsa_miR_507	POTEF
hsa_circ_0003162	hsa_miR_507	PROX1
hsa_circ_0003162	hsa_miR_507	ZEB2
hsa_circ_0003162	hsa_miR_507	MS4A2
hsa_circ_0003162	hsa_miR_507	SOX2
hsa_circ_0003162	hsa_miR_507	TIGIT
hsa_circ_0003162	hsa_miR_507	ELAVL4
hsa_circ_0003162	hsa_miR_507	ARRB1
hsa_circ_0003162	hsa_miR_507	ST8SIA3
hsa_circ_0003162	hsa_miR_507	TP63
hsa_circ_0003162	hsa_miR_507	HECW1
hsa_circ_0003162	hsa_miR_507	SLC5A3
hsa_circ_0003162	hsa_miR_507	TNFRSF9
hsa_circ_0003162	hsa_miR_507	STK32B
hsa_circ_0003162	hsa_miR_507	RSPH10B
hsa_circ_0003162	hsa_miR_507	SLC16A7
hsa_circ_0003162	hsa_miR_507	CNTNAP3B
hsa_circ_0003162	hsa_miR_507	RUSC1
hsa_circ_0003162	hsa_miR_507	CYP2C9
hsa_circ_0003162	hsa_miR_507	PDCD6
hsa_circ_0003162	hsa_miR_507	PABPC3
hsa_circ_0003162	hsa_miR_507	HOXC13
hsa_circ_0003162	hsa_miR_507	NEUROD1
hsa_circ_0003162	hsa_miR_507	PLEKHO2
hsa_circ_0003162	hsa_miR_507	KLHL23
hsa_circ_0003162	hsa_miR_507	PCDH17
hsa_circ_0003162	hsa_miR_507	CYP2C19
hsa_circ_0003162	hsa_miR_507	ZPLD1
hsa_circ_0003162	hsa_miR_507	PABPC1L
hsa_circ_0003162	hsa_miR_507	MSRB3
hsa_circ_0003162	hsa_miR_507	ETS1
hsa_circ_0003162	hsa_miR_507	QKI
hsa_circ_0003162	hsa_miR_507	CALCRL
hsa_circ_0003162	hsa_miR_507	PRLR
hsa_circ_0003162	hsa_miR_507	ZIC5
hsa_circ_0003162	hsa_miR_507	NCAPD3

hsa_circ_0003162	hsa_miR_507	PRR9
hsa_circ_0003162	hsa_miR_507	SLC39A8
hsa_circ_0003162	hsa_miR_507	IFNB1
hsa_circ_0003162	hsa_miR_507	CFL2
hsa_circ_0003162	hsa_miR_507	FAM167B
hsa_circ_0003162	hsa_miR_507	KLHL34
hsa_circ_0003162	hsa_miR_507	NOS1
hsa_circ_0003162	hsa_miR_507	PGR
hsa_circ_0003162	hsa_miR_507	KCNMB2
hsa_circ_0003162	hsa_miR_507	TNFSF11
hsa_circ_0003162	hsa_miR_507	GDF6
hsa_circ_0003162	hsa_miR_507	DUS1L
hsa_circ_0003162	hsa_miR_507	WWC2
hsa_circ_0003162	hsa_miR_507	HEY2
hsa_circ_0003162	hsa_miR_507	CNNM1
hsa_circ_0003162	hsa_miR_507	CAMK2A
hsa_circ_0003162	hsa_miR_507	OSBPL6
hsa_circ_0003162	hsa_miR_507	P2RY14
hsa_circ_0003162	hsa_miR_507	PCDH7
hsa_circ_0003162	hsa_miR_507	PLCE1
hsa_circ_0003162	hsa_miR_507	PAPSS2
hsa_circ_0003162	hsa_miR_507	MSI2
hsa_circ_0003162	hsa_miR_507	PDE5A
hsa_circ_0003162	hsa_miR_507	SDC2
hsa_circ_0003162	hsa_miR_507	SDS
hsa_circ_0003162	hsa_miR_507	ATP2B2
hsa_circ_0003162	hsa_miR_507	GUCY1A2
hsa_circ_0003162	hsa_miR_507	PTPRZ1
hsa_circ_0003162	hsa_miR_507	HCN1
hsa_circ_0003162	hsa_miR_507	KCND2
hsa_circ_0003162	hsa_miR_507	IL22RA2
hsa_circ_0003162	hsa_miR_507	FERMT1
hsa_circ_0003162	hsa_miR_507	ZNF710
hsa_circ_0003162	hsa_miR_507	DCBLD2
hsa_circ_0003162	hsa_miR_507	KCNB1
hsa_circ_0003162	hsa_miR_507	GFRA2
hsa_circ_0003162	hsa_miR_507	ASCL1
hsa_circ_0003162	hsa_miR_507	SLC9A7
hsa_circ_0003162	hsa_miR_507	LRRC8C
hsa_circ_0003162	hsa_miR_507	TUBA4A
hsa_circ_0003162	hsa_miR_507	DSN1
hsa_circ_0003162	hsa_miR_507	ELOVL6
hsa_circ_0003162	hsa_miR_507	RFX6
hsa_circ_0003162	hsa_miR_507	FAXC
hsa_circ_0003162	hsa_miR_507	DLX4
hsa_circ_0003162	hsa_miR_507	SYNE1
hsa_circ_0003162	hsa_miR_507	TFPI2
hsa_circ_0003162	hsa_miR_507	TCF4
hsa_circ_0003162	hsa_miR_507	FCRL2
hsa_circ_0003162	hsa_miR_507	SLC34A3
hsa_circ_0003162	hsa_miR_507	DLC1
hsa_circ_0003162	hsa_miR_507	LRRC2
hsa_circ_0003162	hsa_miR_507	SYTL5
hsa_circ_0003162	hsa_miR_507	GABBR2
hsa_circ_0003162	hsa_miR_507	PKIB
hsa_circ_0003162	hsa_miR_507	PRICKLE1
hsa_circ_0003162	hsa_miR_507	STARD9
hsa_circ_0003162	hsa_miR_507	NT5E

hsa_circ_0003162	hsa_miR_507	XKR9
hsa_circ_0003162	hsa_miR_507	SEMA6A
hsa_circ_0003162	hsa_miR_507	RORA
hsa_circ_0003162	hsa_miR_507	PAX2
hsa_circ_0003162	hsa_miR_507	PI15
hsa_circ_0003162	hsa_miR_507	UNC13C
hsa_circ_0003162	hsa_miR_507	KNOP1
hsa_circ_0003162	hsa_miR_507	EMX2
hsa_circ_0003162	hsa_miR_507	ZBTB20
hsa_circ_0003162	hsa_miR_507	MEOX2
hsa_circ_0003162	hsa_miR_507	IDO2
hsa_circ_0003162	hsa_miR_507	TMEM255A
hsa_circ_0003162	hsa_miR_507	SCARF1
hsa_circ_0003162	hsa_miR_507	TRIM59
hsa_circ_0003162	hsa_miR_507	CCBE1
hsa_circ_0003162	hsa_miR_507	CACNA1B
hsa_circ_0003162	hsa_miR_507	SLAMF7
hsa_circ_0003162	hsa_miR_507	LINGO2
hsa_circ_0003162	hsa_miR_507	CBX3
hsa_circ_0003162	hsa_miR_507	SOSTDC1
hsa_circ_0003162	hsa_miR_507	FOXE1
hsa_circ_0003162	hsa_miR_507	SGMS2
hsa_circ_0003162	hsa_miR_507	ERBB4
hsa_circ_0003162	hsa_miR_507	EMILIN2
hsa_circ_0003162	hsa_miR_507	PAK3
hsa_circ_0003162	hsa_miR_507	CD93
hsa_circ_0003162	hsa_miR_507	CCDC170
hsa_circ_0003162	hsa_miR_507	CDKN2A
hsa_circ_0003162	hsa_miR_507	MARCKSL1
hsa_circ_0003162	hsa_miR_507	FAM135B
hsa_circ_0003162	hsa_miR_507	CAT
hsa_circ_0003162	hsa_miR_507	TBX18
hsa_circ_0003162	hsa_miR_507	CAPN14
hsa_circ_0003162	hsa_miR_507	PAX9
hsa_circ_0003162	hsa_miR_1286	PCDH17
hsa_circ_0003162	hsa_miR_1286	DRC1
hsa_circ_0003162	hsa_miR_1286	IPCEF1
hsa_circ_0003162	hsa_miR_1286	PCSK6
hsa_circ_0003162	hsa_miR_1286	ASPH
hsa_circ_0003162	hsa_miR_1286	GAS7
hsa_circ_0003162	hsa_miR_1286	C20orf197
hsa_circ_0003162	hsa_miR_1286	IGSF9B
hsa_circ_0003162	hsa_miR_1286	ZNF423
hsa_circ_0003162	hsa_miR_1286	DYNAP
hsa_circ_0003162	hsa_miR_1286	DIRAS1
hsa_circ_0003162	hsa_miR_1286	ARAP2
hsa_circ_0003162	hsa_miR_1286	DGKI
hsa_circ_0003162	hsa_miR_1286	WNT1
hsa_circ_0003162	hsa_miR_1286	MAGEC2
hsa_circ_0003162	hsa_miR_1286	DDX11
hsa_circ_0003162	hsa_miR_1286	FBXO41
hsa_circ_0003162	hsa_miR_1286	MMP16
hsa_circ_0003162	hsa_miR_1286	SLC24A4
hsa_circ_0003162	hsa_miR_1286	TH
hsa_circ_0003162	hsa_miR_1286	NPHS1
hsa_circ_0003162	hsa_miR_1286	NFASC
hsa_circ_0003162	hsa_miR_1286	F8
hsa_circ_0003162	hsa_miR_1286	KCNE1

hsa_circ_0003162	hsa_miR_1286	ASF1B
hsa_circ_0003162	hsa_miR_1286	RAB11FIP1
hsa_circ_0003162	hsa_miR_1286	TMEM132E
hsa_circ_0003162	hsa_miR_1286	NAT9
hsa_circ_0003162	hsa_miR_1286	TXNDC17
hsa_circ_0003162	hsa_miR_1286	FAM155B
hsa_circ_0003162	hsa_miR_1286	WNK3
hsa_circ_0003162	hsa_miR_1286	SNTG2
hsa_circ_0003162	hsa_miR_1286	DBNDD1
hsa_circ_0003162	hsa_miR_1286	LGALSL
hsa_circ_0003162	hsa_miR_1286	CHRD
hsa_circ_0003162	hsa_miR_1286	AVPR2
hsa_circ_0003162	hsa_miR_1286	NOS1
hsa_circ_0003162	hsa_miR_1286	PCP4
hsa_circ_0003162	hsa_miR_1286	CPLX1
hsa_circ_0003162	hsa_miR_1286	RASGEF1B
hsa_circ_0003162	hsa_miR_1286	CDCA5
hsa_circ_0003162	hsa_miR_1286	NTNG2
hsa_circ_0003162	hsa_miR_1286	PTPN5
hsa_circ_0003162	hsa_miR_1286	OPRK1
hsa_circ_0003162	hsa_miR_1286	NGB
hsa_circ_0003162	hsa_miR_1286	PCYT1B
hsa_circ_0003162	hsa_miR_1286	CHAF1A
hsa_circ_0003162	hsa_miR_1286	CDKN2A
hsa_circ_0003162	hsa_miR_1286	EGLN3
hsa_circ_0003162	hsa_miR_1286	FBXL19
hsa_circ_0003162	hsa_miR_1286	CAB39L
hsa_circ_0003162	hsa_miR_1286	SH2D4B
hsa_circ_0003162	hsa_miR_1286	KRTAP5-7
hsa_circ_0003162	hsa_miR_1286	SMOX
hsa_circ_0003162	hsa_miR_1286	CD68
hsa_circ_0003162	hsa_miR_1286	CAMKV
hsa_circ_0003162	hsa_miR_1286	NMNAT2
hsa_circ_0003162	hsa_miR_1286	PXDC1
hsa_circ_0003162	hsa_miR_1286	SLC7A14
hsa_circ_0003162	hsa_miR_1286	KCNK3
hsa_circ_0003162	hsa_miR_1286	CALCA
hsa_circ_0003162	hsa_miR_1286	DOCK3
hsa_circ_0003162	hsa_miR_1286	ABCB9
hsa_circ_0003162	hsa_miR_1286	RORA
hsa_circ_0003162	hsa_miR_1286	KDM5B
hsa_circ_0003162	hsa_miR_1286	ELAVL2
hsa_circ_0003162	hsa_miR_1286	DAO
hsa_circ_0003162	hsa_miR_1286	PPP1R16A
hsa_circ_0003162	hsa_miR_1286	GRIK3
hsa_circ_0003162	hsa_miR_1286	RD3
hsa_circ_0003162	hsa_miR_1286	ADPRHL1
hsa_circ_0003162	hsa_miR_1286	RCC1
hsa_circ_0003162	hsa_miR_1286	DPT
hsa_circ_0003162	hsa_miR_1286	CHEK1
hsa_circ_0003162	hsa_miR_1286	SLC26A4
hsa_circ_0003162	hsa_miR_1286	PXT1
hsa_circ_0003162	hsa_miR_1286	KRT79
hsa_circ_0003162	hsa_miR_1286	FCRLA
hsa_circ_0003162	hsa_miR_1286	NTNG1
hsa_circ_0003162	hsa_miR_1286	STOML3
hsa_circ_0003162	hsa_miR_1286	ADAMTS19
hsa_circ_0003162	hsa_miR_1286	MMP8

hsa_circ_0003162	hsa_miR_1286	DOCK4
hsa_circ_0003162	hsa_miR_1286	AMOTL1
hsa_circ_0003162	hsa_miR_1286	KCNAB2
hsa_circ_0003162	hsa_miR_1286	LCT
hsa_circ_0003162	hsa_miR_1286	SYNPO
hsa_circ_0003162	hsa_miR_1286	TRHDE
hsa_circ_0003162	hsa_miR_1286	TSHR
hsa_circ_0003162	hsa_miR_1286	FHL1
hsa_circ_0003162	hsa_miR_1286	PNPLA5
hsa_circ_0003162	hsa_miR_1286	REG1B
hsa_circ_0003162	hsa_miR_1286	STON1
hsa_circ_0003162	hsa_miR_1286	ROBO2
hsa_circ_0003162	hsa_miR_1286	APOLD1
hsa_circ_0003162	hsa_miR_1286	APOC3
hsa_circ_0003162	hsa_miR_1286	COBL
hsa_circ_0003162	hsa_miR_1286	MFAP4
hsa_circ_0003162	hsa_miR_1286	IGDCC3
hsa_circ_0003162	hsa_miR_1286	CDK5R1
hsa_circ_0003162	hsa_miR_1286	BEST2
hsa_circ_0003162	hsa_miR_1286	MCM4
hsa_circ_0003162	hsa_miR_1286	C1orf226
hsa_circ_0003162	hsa_miR_1286	NIPSNAP1
hsa_circ_0003162	hsa_miR_1286	CNR1
hsa_circ_0003162	hsa_miR_1286	IGF2
hsa_circ_0003162	hsa_miR_1286	GABBR2
hsa_circ_0003162	hsa_miR_1286	ZIC5
hsa_circ_0003162	hsa_miR_1286	GPR158
hsa_circ_0003162	hsa_miR_1286	GJA3
hsa_circ_0003162	hsa_miR_1286	MMP10
hsa_circ_0003162	hsa_miR_1286	GOLGA7B
hsa_circ_0003162	hsa_miR_1286	GRIN2A
hsa_circ_0003162	hsa_miR_1286	POLE
hsa_circ_0003162	hsa_miR_1286	EMCN
hsa_circ_0003162	hsa_miR_1286	SLC6A3
hsa_circ_0003162	hsa_miR_1286	SLC24A2
hsa_circ_0003162	hsa_miR_1286	CEACAM1
hsa_circ_0003162	hsa_miR_1286	CAMK2A
hsa_circ_0003162	hsa_miR_1286	AK4
hsa_circ_0003162	hsa_miR_1286	GAP43
hsa_circ_0003162	hsa_miR_1286	PIK3R5
hsa_circ_0003162	hsa_miR_1286	LIN28A
hsa_circ_0003162	hsa_miR_1286	COX6B2
hsa_circ_0003162	hsa_miR_1286	ADARB1
hsa_circ_0003162	hsa_miR_1286	CNRIP1
hsa_circ_0003162	hsa_miR_1286	GALNTL6
hsa_circ_0003162	hsa_miR_1286	HPCA
hsa_circ_0003162	hsa_miR_1286	CYBB
hsa_circ_0003162	hsa_miR_1286	SIAH3
hsa_circ_0003162	hsa_miR_1286	GAL3ST3
hsa_circ_0003162	hsa_miR_1286	REPS2
hsa_circ_0003162	hsa_miR_495	CADM1
hsa_circ_0003162	hsa_miR_495	DDIT4
hsa_circ_0003162	hsa_miR_495	BDNF
hsa_circ_0003162	hsa_miR_495	CELF2
hsa_circ_0003162	hsa_miR_495	THBS2
hsa_circ_0003162	hsa_miR_495	NTNG1
hsa_circ_0003162	hsa_miR_495	ATP2B2
hsa_circ_0003162	hsa_miR_495	TCF4

hsa_circ_0003162	hsa_miR_495	ETS1
hsa_circ_0003162	hsa_miR_495	PAQR9
hsa_circ_0003162	hsa_miR_495	CYP26B1
hsa_circ_0003162	hsa_miR_532-3p	NDP
hsa_circ_0003162	hsa_miR_532-3p	CLVS2
hsa_circ_0003162	hsa_miR_532-3p	CXCL2
hsa_circ_0003162	hsa_miR_532-3p	SLC39A8
hsa_circ_0003162	hsa_miR_532-3p	LINGO2
hsa_circ_0003162	hsa_miR_532-3p	NKD1
hsa_circ_0003162	hsa_miR_532-3p	ERCC6L
hsa_circ_0003162	hsa_miR_532-3p	NSG2
hsa_circ_0003162	hsa_miR_532-3p	TRHDE
hsa_circ_0003162	hsa_miR_532-3p	CHL1
hsa_circ_0003162	hsa_miR_532-3p	CPD
hsa_circ_0003162	hsa_miR_507	RBMS3
hsa_circ_0003162	hsa_miR_507	SCN3A
hsa_circ_0003162	hsa_miR_507	PRKCE
hsa_circ_0003162	hsa_miR_507	MDGA2
hsa_circ_0003162	hsa_miR_507	LPGAT1
hsa_circ_0003162	hsa_miR_507	KCNN3
hsa_circ_0003162	hsa_miR_507	CBX4
hsa_circ_0003162	hsa_miR_507	TET1
hsa_circ_0003162	hsa_miR_507	SCN1A
hsa_circ_0003162	hsa_miR_507	RIMS2
hsa_circ_0003162	hsa_miR_507	UNC13A
hsa_circ_0003162	hsa_miR_507	GRP
hsa_circ_0003162	hsa_miR_507	ISX
hsa_circ_0003162	hsa_miR_507	AMPH
hsa_circ_0003162	hsa_miR_507	FST
hsa_circ_0003162	hsa_miR_507	GRIK2
hsa_circ_0003162	hsa_miR_507	TRPM1
hsa_circ_0003162	hsa_miR_507	OLIG3
hsa_circ_0003162	hsa_miR_507	CTNNA2
hsa_circ_0003162	hsa_miR_507	GRIP1
hsa_circ_0003162	hsa_miR_507	NELL2
hsa_circ_0003162	hsa_miR_507	DAB2IP
hsa_circ_0003162	hsa_miR_507	DKK2
hsa_circ_0003162	hsa_miR_507	SECISBP2L
hsa_circ_0003162	hsa_miR_507	PAX6
hsa_circ_0003162	hsa_miR_507	CRTAM
hsa_circ_0003162	hsa_miR_507	CNKSR2
hsa_circ_0003162	hsa_miR_507	FAM43A
hsa_circ_0003162	hsa_miR_507	MEIS1
hsa_circ_0003162	hsa_miR_507	KDELR2
hsa_circ_0003162	hsa_miR_507	TRIP13
hsa_circ_0003162	hsa_miR_507	SYT14
hsa_circ_0003162	hsa_miR_507	LHX8
hsa_circ_0003162	hsa_miR_507	MLLT11
hsa_circ_0003162	hsa_miR_507	SEMA6D
hsa_circ_0003162	hsa_miR_507	PTPRM
hsa_circ_0003162	hsa_miR_507	LRRN1
hsa_circ_0003162	hsa_miR_507	PTPRD
hsa_circ_0003162	hsa_miR_507	FLRT3
hsa_circ_0003162	hsa_miR_507	PF4
hsa_circ_0003162	hsa_miR_507	ATP1B1
hsa_circ_0003162	hsa_miR_507	E2F7
hsa_circ_0003162	hsa_miR_507	LCN10
hsa_circ_0003162	hsa_miR_507	GLRA3

hsa_circ_0003162	hsa_miR_507	DTL
hsa_circ_0003162	hsa_miR_507	PABPC1
hsa_circ_0003162	hsa_miR_507	CHODL
hsa_circ_0003162	hsa_miR_507	LCOR
hsa_circ_0003162	hsa_miR_507	AVL9
hsa_circ_0003162	hsa_miR_507	PHKA1
hsa_circ_0003162	hsa_miR_507	EPHB1
hsa_circ_0003162	hsa_miR_507	JAKMIP3
hsa_circ_0003162	hsa_miR_507	KCNH7
hsa_circ_0003162	hsa_miR_507	ZIC1
hsa_circ_0003162	hsa_miR_507	TMTC4
hsa_circ_0003162	hsa_miR_507	DAZL
hsa_circ_0003162	hsa_miR_507	EMP1
hsa_circ_0003162	hsa_miR_507	CCND2
hsa_circ_0003162	hsa_miR_507	GNAQ
hsa_circ_0003162	hsa_miR_507	NECAB1
hsa_circ_0003162	hsa_miR_507	SIM1
hsa_circ_0003162	hsa_miR_507	EPHA8
hsa_circ_0003162	hsa_miR_507	DLK1
hsa_circ_0003162	hsa_miR_507	SPTSSB
hsa_circ_0003162	hsa_miR_507	RUNX2
hsa_circ_0003162	hsa_miR_507	PKHD1
hsa_circ_0003162	hsa_miR_507	FGFBP2
hsa_circ_0003162	hsa_miR_507	GABRA4
hsa_circ_0003162	hsa_miR_507	CACNA1E
hsa_circ_0003162	hsa_miR_507	NKX2-2
hsa_circ_0003162	hsa_miR_507	FBN2
hsa_circ_0003162	hsa_miR_507	SIX3
hsa_circ_0003162	hsa_miR_507	VSX1
hsa_circ_0003162	hsa_miR_507	AK5
hsa_circ_0003162	hsa_miR_507	MCIDAS
hsa_circ_0003162	hsa_miR_507	ZNF681
hsa_circ_0003162	hsa_miR_507	SYNM
hsa_circ_0003162	hsa_miR_507	SATB2
hsa_circ_0003162	hsa_miR_507	POTEF
hsa_circ_0003162	hsa_miR_507	PROX1
hsa_circ_0003162	hsa_miR_507	ZEB2
hsa_circ_0003162	hsa_miR_507	MS4A2
hsa_circ_0003162	hsa_miR_507	SOX2
hsa_circ_0003162	hsa_miR_507	TIGIT
hsa_circ_0003162	hsa_miR_507	ELAVL4
hsa_circ_0003162	hsa_miR_507	ARRB1
hsa_circ_0003162	hsa_miR_507	ST8SIA3
hsa_circ_0003162	hsa_miR_507	TP63
hsa_circ_0003162	hsa_miR_507	HECW1
hsa_circ_0003162	hsa_miR_507	SLC5A3
hsa_circ_0003162	hsa_miR_507	TNFRSF9
hsa_circ_0003162	hsa_miR_507	STK32B
hsa_circ_0003162	hsa_miR_507	RSPH10B
hsa_circ_0003162	hsa_miR_507	SLC16A7
hsa_circ_0003162	hsa_miR_507	CNTNAP3B
hsa_circ_0003162	hsa_miR_507	RUSC1
hsa_circ_0003162	hsa_miR_507	CYP2C9
hsa_circ_0003162	hsa_miR_507	PDCD6
hsa_circ_0003162	hsa_miR_507	PABPC3
hsa_circ_0003162	hsa_miR_507	HOXC13
hsa_circ_0003162	hsa_miR_507	NEUROD1
hsa_circ_0003162	hsa_miR_507	PLEKHO2

hsa_circ_0003162	hsa_miR_507	KLHL23
hsa_circ_0003162	hsa_miR_507	PCDH17
hsa_circ_0003162	hsa_miR_507	CYP2C19
hsa_circ_0003162	hsa_miR_507	ZPLD1
hsa_circ_0003162	hsa_miR_507	PABPC1L
hsa_circ_0003162	hsa_miR_507	MSRB3
hsa_circ_0003162	hsa_miR_507	ETS1
hsa_circ_0003162	hsa_miR_507	QKI
hsa_circ_0003162	hsa_miR_507	CALCRL
hsa_circ_0003162	hsa_miR_507	PRLR
hsa_circ_0003162	hsa_miR_507	ZIC5
hsa_circ_0003162	hsa_miR_507	NCAPD3
hsa_circ_0003162	hsa_miR_507	PRR9
hsa_circ_0003162	hsa_miR_507	SLC39A8
hsa_circ_0003162	hsa_miR_507	IFNB1
hsa_circ_0003162	hsa_miR_507	CFL2
hsa_circ_0003162	hsa_miR_507	FAM167B
hsa_circ_0003162	hsa_miR_507	KLHL34
hsa_circ_0003162	hsa_miR_507	NOS1
hsa_circ_0003162	hsa_miR_507	PGR
hsa_circ_0003162	hsa_miR_507	KCNMB2
hsa_circ_0003162	hsa_miR_507	TNFSF11
hsa_circ_0003162	hsa_miR_507	GDF6
hsa_circ_0003162	hsa_miR_507	DUS1L
hsa_circ_0003162	hsa_miR_507	WWC2
hsa_circ_0003162	hsa_miR_507	HEY2
hsa_circ_0003162	hsa_miR_507	CNNM1
hsa_circ_0003162	hsa_miR_507	CAMK2A
hsa_circ_0003162	hsa_miR_507	OSBPL6
hsa_circ_0003162	hsa_miR_507	P2RY14
hsa_circ_0003162	hsa_miR_507	PCDH7
hsa_circ_0003162	hsa_miR_507	PLCE1
hsa_circ_0003162	hsa_miR_507	PAPSS2
hsa_circ_0003162	hsa_miR_507	MSI2
hsa_circ_0003162	hsa_miR_507	PDE5A
hsa_circ_0003162	hsa_miR_507	SDC2
hsa_circ_0003162	hsa_miR_507	SDS
hsa_circ_0003162	hsa_miR_507	ATP2B2
hsa_circ_0003162	hsa_miR_507	GUCY1A2
hsa_circ_0003162	hsa_miR_507	PTPRZ1
hsa_circ_0003162	hsa_miR_507	HCN1
hsa_circ_0003162	hsa_miR_507	KCND2
hsa_circ_0003162	hsa_miR_507	IL22RA2
hsa_circ_0003162	hsa_miR_507	FERMT1
hsa_circ_0003162	hsa_miR_507	ZNF710
hsa_circ_0003162	hsa_miR_507	DCBLD2
hsa_circ_0003162	hsa_miR_507	KCNB1
hsa_circ_0003162	hsa_miR_507	GFRA2
hsa_circ_0003162	hsa_miR_507	ASCL1
hsa_circ_0003162	hsa_miR_507	SLC9A7
hsa_circ_0003162	hsa_miR_507	LRRC8C
hsa_circ_0003162	hsa_miR_507	TUBA4A
hsa_circ_0003162	hsa_miR_507	DSN1
hsa_circ_0003162	hsa_miR_507	ELOVL6
hsa_circ_0003162	hsa_miR_507	RFX6
hsa_circ_0003162	hsa_miR_507	FAXC
hsa_circ_0003162	hsa_miR_507	DLX4
hsa_circ_0003162	hsa_miR_507	SYNE1

hsa_circ_0003162	hsa_miR_507	TFPI2
hsa_circ_0003162	hsa_miR_507	TCF4
hsa_circ_0003162	hsa_miR_507	FCRL2
hsa_circ_0003162	hsa_miR_507	SLC34A3
hsa_circ_0003162	hsa_miR_507	DLC1
hsa_circ_0003162	hsa_miR_507	LRRC2
hsa_circ_0003162	hsa_miR_507	SYTL5
hsa_circ_0003162	hsa_miR_507	GABBR2
hsa_circ_0003162	hsa_miR_507	PKIB
hsa_circ_0003162	hsa_miR_507	PRICKLE1
hsa_circ_0003162	hsa_miR_507	STARD9
hsa_circ_0003162	hsa_miR_507	NT5E
hsa_circ_0003162	hsa_miR_507	XKR9
hsa_circ_0003162	hsa_miR_507	SEMA6A
hsa_circ_0003162	hsa_miR_507	RORA
hsa_circ_0003162	hsa_miR_507	PAX2
hsa_circ_0003162	hsa_miR_507	PI15
hsa_circ_0003162	hsa_miR_507	UNC13C
hsa_circ_0003162	hsa_miR_507	KNOP1
hsa_circ_0003162	hsa_miR_507	EMX2
hsa_circ_0003162	hsa_miR_507	ZBTB20
hsa_circ_0003162	hsa_miR_507	MEOX2
hsa_circ_0003162	hsa_miR_507	IDO2
hsa_circ_0003162	hsa_miR_507	TMEM255A
hsa_circ_0003162	hsa_miR_507	SCARF1
hsa_circ_0003162	hsa_miR_507	TRIM59
hsa_circ_0003162	hsa_miR_507	CCBE1
hsa_circ_0003162	hsa_miR_507	CACNA1B
hsa_circ_0003162	hsa_miR_507	SLAMF7
hsa_circ_0003162	hsa_miR_507	LINGO2
hsa_circ_0003162	hsa_miR_507	CBX3
hsa_circ_0003162	hsa_miR_507	SOSTDC1
hsa_circ_0003162	hsa_miR_507	FOXE1
hsa_circ_0003162	hsa_miR_507	SGMS2
hsa_circ_0003162	hsa_miR_507	ERBB4
hsa_circ_0003162	hsa_miR_507	EMILIN2
hsa_circ_0003162	hsa_miR_507	PAK3
hsa_circ_0003162	hsa_miR_507	CD93
hsa_circ_0003162	hsa_miR_507	CCDC170
hsa_circ_0003162	hsa_miR_507	CDKN2A
hsa_circ_0003162	hsa_miR_507	MARCKSL1
hsa_circ_0003162	hsa_miR_507	FAM135B
hsa_circ_0003162	hsa_miR_507	CAT
hsa_circ_0003162	hsa_miR_507	TBX18
hsa_circ_0003162	hsa_miR_507	CAPN14
hsa_circ_0003162	hsa_miR_507	PAX9
hsa_circ_0003162	hsa_miR_1206	SNTG1
hsa_circ_0003162	hsa_miR_1206	TMEFF1
hsa_circ_0003162	hsa_miR_1206	KCND2
hsa_circ_0003162	hsa_miR_1206	SEMA6A
hsa_circ_0003162	hsa_miR_1206	KCNQ5
hsa_circ_0003162	hsa_miR_1206	HOXA1
hsa_circ_0003162	hsa_miR_1206	LRP1B
hsa_circ_0003162	hsa_miR_1206	NDNF
hsa_circ_0003162	hsa_miR_1206	FUT8
hsa_circ_0003162	hsa_miR_1206	NR5A2
hsa_circ_0003162	hsa_miR_1206	DSCC1
hsa_circ_0003162	hsa_miR_1206	DOK6

hsa_circ_0003162	hsa_miR_1206	SKA3
hsa_circ_0003162	hsa_miR_1206	THRB
hsa_circ_0003162	hsa_miR_1206	KLHL32
hsa_circ_0003162	hsa_miR_1206	LRRC31
hsa_circ_0003162	hsa_miR_1206	KCNB2
hsa_circ_0003162	hsa_miR_1206	TIMP3
hsa_circ_0003162	hsa_miR_1206	SHISA3
hsa_circ_0003162	hsa_miR_1206	TPBG
hsa_circ_0003162	hsa_miR_1206	VSTM4
hsa_circ_0003162	hsa_miR_1206	GRIK2
hsa_circ_0003162	hsa_miR_1206	FAT1
hsa_circ_0003162	hsa_miR_1206	HTR4
hsa_circ_0003162	hsa_miR_1206	RHNO1
hsa_circ_0003162	hsa_miR_1206	PHEX
hsa_circ_0003162	hsa_miR_1206	NTN4
hsa_circ_0003162	hsa_miR_1206	TLL2
hsa_circ_0003162	hsa_miR_1206	QKI
hsa_circ_0003162	hsa_miR_1206	NIPSNAP1
hsa_circ_0003162	hsa_miR_1206	OPRK1
hsa_circ_0003162	hsa_miR_1206	GCLM
hsa_circ_0003162	hsa_miR_1206	ADAMTS19
hsa_circ_0003162	hsa_miR_1206	IGF2
hsa_circ_0003162	hsa_miR_1206	PROS1
hsa_circ_0003162	hsa_miR_1206	FAXC
hsa_circ_0003162	hsa_miR_1206	CCDC141
hsa_circ_0003162	hsa_miR_1206	PHF21B
hsa_circ_0003162	hsa_miR_1206	ABCA10
hsa_circ_0003162	hsa_miR_1206	SASH1
hsa_circ_0003162	hsa_miR_1206	SORBS1
hsa_circ_0003162	hsa_miR_1206	EPHA7
hsa_circ_0003162	hsa_miR_1206	TIMM8A
hsa_circ_0003162	hsa_miR_1206	ZBTB20
hsa_circ_0003162	hsa_miR_1206	MRAS
hsa_circ_0003162	hsa_miR_1206	SLC27A2
hsa_circ_0003162	hsa_miR_1206	FUT9
hsa_circ_0003162	hsa_miR_1206	FAM216B
hsa_circ_0003162	hsa_miR_1206	CCBE1
hsa_circ_0003162	hsa_miR_1206	CHML
hsa_circ_0003162	hsa_miR_1206	GDAP1L1
hsa_circ_0003162	hsa_miR_1206	F2RL2
hsa_circ_0003162	hsa_miR_1206	ALDH1L2
hsa_circ_0003162	hsa_miR_1206	BARD1
hsa_circ_0003162	hsa_miR_1206	NKAIN3
hsa_circ_0003162	hsa_miR_1206	ZNF367
hsa_circ_0003162	hsa_miR_1206	RIPPLY3
hsa_circ_0003162	hsa_miR_1206	RBMS3
hsa_circ_0003162	hsa_miR_1206	SHROOM4
hsa_circ_0003162	hsa_miR_1206	CAMK2B
hsa_circ_0003162	hsa_miR_1206	C1QTNF7
hsa_circ_0003162	hsa_miR_1206	SSTR1
hsa_circ_0003162	hsa_miR_1206	NTM
hsa_circ_0003162	hsa_miR_1206	EGLN3
hsa_circ_0003162	hsa_miR_1206	SYTL5
hsa_circ_0003162	hsa_miR_1206	DUSP4
hsa_circ_0003162	hsa_miR_1206	SCN3A
hsa_circ_0003162	hsa_miR_1206	ROR1
hsa_circ_0003162	hsa_miR_1206	SEMA3A
hsa_circ_0003162	hsa_miR_1206	RASSF6

hsa_circ_0003162	hsa_miR_1206	KCNB1
hsa_circ_0003162	hsa_miR_1206	PEAR1
hsa_circ_0003162	hsa_miR_1206	CDH10
hsa_circ_0003162	hsa_miR_1206	GUCY1A2
hsa_circ_0003162	hsa_miR_1206	MITF
hsa_circ_0003162	hsa_miR_1206	NOVA1
hsa_circ_0003162	hsa_miR_1206	RS1
hsa_circ_0003162	hsa_miR_1206	SLC5A7
hsa_circ_0003162	hsa_miR_1206	KCNK15
hsa_circ_0003162	hsa_miR_1206	CBR1
hsa_circ_0003162	hsa_miR_1206	VSX2
hsa_circ_0003162	hsa_miR_1206	PRDM5
hsa_circ_0003162	hsa_miR_1206	FRMD3
hsa_circ_0003162	hsa_miR_1206	TFF3
hsa_circ_0003162	hsa_miR_1206	KCNJ1
hsa_circ_0003162	hsa_miR_1206	RASSF8
hsa_circ_0003162	hsa_miR_1206	ROBO2
hsa_circ_0003162	hsa_miR_1206	RASSF2
hsa_circ_0003162	hsa_miR_1206	SLC2A2
hsa_circ_0003162	hsa_miR_1206	RD3
hsa_circ_0003162	hsa_miR_1206	IL5RA
hsa_circ_0003162	hsa_miR_1206	SCAI
hsa_circ_0003162	hsa_miR_1206	CREG2
hsa_circ_0003162	hsa_miR_1206	NSG2
hsa_circ_0003162	hsa_miR_1206	TMEM132C
hsa_circ_0003162	hsa_miR_1206	CHL1
hsa_circ_0003162	hsa_miR_1206	C20orf197
hsa_circ_0003528	hsa_miR_215	EREG
hsa_circ_0003528	hsa_miR_215	NIPAL1
hsa_circ_0003528	hsa_miR_215	GPR22
hsa_circ_0003528	hsa_miR_215	NKAIN2
hsa_circ_0003528	hsa_miR_215	ZEB2
hsa_circ_0003528	hsa_miR_215	FRMD4B
hsa_circ_0003528	hsa_miR_215	OLIG3
hsa_circ_0003528	hsa_miR_215	ZNF536
hsa_circ_0003528	hsa_miR_215	MYLK
hsa_circ_0003528	hsa_miR_215	RAD54B
hsa_circ_0003528	hsa_miR_215	GALNTL6
hsa_circ_0003528	hsa_miR_215	IGDCC3
hsa_circ_0003528	hsa_miR_215	FAXC
hsa_circ_0003528	hsa_miR_215	ENC1
hsa_circ_0003528	hsa_miR_215	RUNX1T1
hsa_circ_0003528	hsa_miR_215	COL5A1
hsa_circ_0003528	hsa_miR_215	TCTEX1D1
hsa_circ_0003528	hsa_miR_587	RAD54B
hsa_circ_0003528	hsa_miR_587	COL11A1
hsa_circ_0003528	hsa_miR_587	CCDC181
hsa_circ_0003528	hsa_miR_587	RBFOX1
hsa_circ_0003528	hsa_miR_587	CNTNAP2
hsa_circ_0003528	hsa_miR_587	ZNF781
hsa_circ_0003528	hsa_miR_587	PABPC1
hsa_circ_0003528	hsa_miR_587	ZIC3
hsa_circ_0003528	hsa_miR_587	SIM1
hsa_circ_0003528	hsa_miR_587	GRM3
hsa_circ_0003528	hsa_miR_587	THRB
hsa_circ_0003528	hsa_miR_587	KCND2
hsa_circ_0003528	hsa_miR_587	SLIT3
hsa_circ_0003528	hsa_miR_587	NXPH1

hsa_circ_0003528	hsa_miR_587	SORCS3
hsa_circ_0003528	hsa_miR_587	COL1A2
hsa_circ_0003528	hsa_miR_587	ZNF367
hsa_circ_0003528	hsa_miR_587	GNG11
hsa_circ_0003528	hsa_miR_587	ETV1
hsa_circ_0003528	hsa_miR_587	BMP2
hsa_circ_0003528	hsa_miR_587	ADAM28
hsa_circ_0003528	hsa_miR_587	SMAD6
hsa_circ_0003528	hsa_miR_587	GFRA2
hsa_circ_0003528	hsa_miR_587	SLC2A2
hsa_circ_0003528	hsa_miR_587	MAP3K8
hsa_circ_0003528	hsa_miR_587	BCL2A1
hsa_circ_0003528	hsa_miR_587	LRIG3
hsa_circ_0003528	hsa_miR_587	IYD
hsa_circ_0003528	hsa_miR_587	CACNB4
hsa_circ_0003528	hsa_miR_587	QPCT
hsa_circ_0003528	hsa_miR_587	KCNAB2
hsa_circ_0003528	hsa_miR_587	HGD
hsa_circ_0003528	hsa_miR_587	GAS6
hsa_circ_0003528	hsa_miR_587	GPSM2
hsa_circ_0003528	hsa_miR_587	ESYT3
hsa_circ_0003528	hsa_miR_587	NOX4
hsa_circ_0003528	hsa_miR_587	SLC7A11
hsa_circ_0003528	hsa_miR_587	MS4A4A
hsa_circ_0003528	hsa_miR_587	GAD1
hsa_circ_0003528	hsa_miR_587	NUSAP1
hsa_circ_0003528	hsa_miR_587	NR2E1
hsa_circ_0003528	hsa_miR_587	SLC5A3
hsa_circ_0003528	hsa_miR_587	MYOZ1
hsa_circ_0003528	hsa_miR_587	COL25A1
hsa_circ_0003528	hsa_miR_587	GALNT13
hsa_circ_0003528	hsa_miR_587	SLITRK1
hsa_circ_0003528	hsa_miR_587	ZFHX4
hsa_circ_0003528	hsa_miR_587	MFAP3L
hsa_circ_0003528	hsa_miR_587	SLC6A15
hsa_circ_0003528	hsa_miR_587	NR4A3
hsa_circ_0003528	hsa_miR_587	NKX2-2
hsa_circ_0003528	hsa_miR_587	GRIA2
hsa_circ_0003528	hsa_miR_587	MSRB3
hsa_circ_0003528	hsa_miR_587	ADAMTS18
hsa_circ_0003528	hsa_miR_587	GRIP1
hsa_circ_0003528	hsa_miR_587	TMIGD1
hsa_circ_0003528	hsa_miR_587	GPM6A
hsa_circ_0003528	hsa_miR_587	BET1
hsa_circ_0003528	hsa_miR_587	KAT2B
hsa_circ_0003528	hsa_miR_587	ODC1
hsa_circ_0003528	hsa_miR_587	RTKN2
hsa_circ_0003528	hsa_miR_587	AQP11
hsa_circ_0003528	hsa_miR_587	ZNF92
hsa_circ_0003528	hsa_miR_587	AGBL1
hsa_circ_0003528	hsa_miR_587	KL
hsa_circ_0003528	hsa_miR_587	GABRG2
hsa_circ_0003528	hsa_miR_587	SHOX2
hsa_circ_0003528	hsa_miR_587	GRIK2
hsa_circ_0003528	hsa_miR_587	INHBC
hsa_circ_0003528	hsa_miR_587	MS4A7
hsa_circ_0003528	hsa_miR_587	FAM124A
hsa_circ_0003528	hsa_miR_587	CHAC2

hsa_circ_0003528	hsa_miR_587	PRKAA2
hsa_circ_0003528	hsa_miR_587	MCM4
hsa_circ_0003528	hsa_miR_587	ORC1
hsa_circ_0003528	hsa_miR_587	ZFP42
hsa_circ_0003528	hsa_miR_587	WFDC13
hsa_circ_0003528	hsa_miR_587	LSAMP
hsa_circ_0003528	hsa_miR_587	TLR10
hsa_circ_0003528	hsa_miR_587	ASIC1
hsa_circ_0003528	hsa_miR_587	NCAPG
hsa_circ_0003528	hsa_miR_587	ZC3H12B
hsa_circ_0003528	hsa_miR_587	MLLT11
hsa_circ_0003528	hsa_miR_587	KCNAB1
hsa_circ_0003528	hsa_miR_587	TLR4
hsa_circ_0003528	hsa_miR_587	CCDC152
hsa_circ_0003528	hsa_miR_587	EXOSC5
hsa_circ_0003528	hsa_miR_587	TLL1
hsa_circ_0003528	hsa_miR_587	ZBTB20
hsa_circ_0003528	hsa_miR_587	MYBL1
hsa_circ_0003528	hsa_miR_587	VGLL3
hsa_circ_0003528	hsa_miR_587	DKK1
hsa_circ_0003528	hsa_miR_587	CLDN12
hsa_circ_0003528	hsa_miR_587	ANKRD33B
hsa_circ_0003528	hsa_miR_587	DOCK4
hsa_circ_0003528	hsa_miR_587	E2F8
hsa_circ_0003528	hsa_miR_587	C1QTNF3
hsa_circ_0003528	hsa_miR_587	ITIH6
hsa_circ_0003528	hsa_miR_587	EFEMP1
hsa_circ_0003528	hsa_miR_587	BHMT
hsa_circ_0003528	hsa_miR_587	FHL5
hsa_circ_0003528	hsa_miR_587	GDF10
hsa_circ_0003528	hsa_miR_587	IGFBP1
hsa_circ_0003528	hsa_miR_587	DIXDC1
hsa_circ_0003528	hsa_miR_587	CXCL16
hsa_circ_0003528	hsa_miR_587	CNGA3
hsa_circ_0003528	hsa_miR_587	ZNF716
hsa_circ_0003528	hsa_miR_587	FAM81A
hsa_circ_0003528	hsa_miR_587	CEACAM8
hsa_circ_0003528	hsa_miR_587	LIN28B
hsa_circ_0003528	hsa_miR_587	RFX6
hsa_circ_0003528	hsa_miR_587	STARD13
hsa_circ_0003528	hsa_miR_587	TMEFF1
hsa_circ_0003528	hsa_miR_587	GCLM
hsa_circ_0003528	hsa_miR_587	GATA6
hsa_circ_0003528	hsa_miR_587	KCNJ1
hsa_circ_0003528	hsa_miR_587	CERS6
hsa_circ_0003528	hsa_miR_587	GABRA4
hsa_circ_0003528	hsa_miR_587	MARVELD3
hsa_circ_0003528	hsa_miR_587	PRELP
hsa_circ_0003528	hsa_miR_587	NFATC1
hsa_circ_0003528	hsa_miR_587	FAM13C
hsa_circ_0003528	hsa_miR_587	ARHGAP29
hsa_circ_0003528	hsa_miR_587	PDGFRL
hsa_circ_0003528	hsa_miR_587	HPS5
hsa_circ_0003528	hsa_miR_587	F2RL2
hsa_circ_0003528	hsa_miR_587	NFE2L3
hsa_circ_0003528	hsa_miR_587	GPR26
hsa_circ_0003528	hsa_miR_587	MTMR10
hsa_circ_0003528	hsa_miR_587	CDH19

hsa_circ_0003528	hsa_miR_587	TSPAN12
hsa_circ_0003528	hsa_miR_587	C5orf49
hsa_circ_0003528	hsa_miR_587	CDH10
hsa_circ_0003528	hsa_miR_587	SHCBP1
hsa_circ_0003528	hsa_miR_587	SEMA3D
hsa_circ_0003528	hsa_miR_587	SYNDIG1
hsa_circ_0003528	hsa_miR_587	FBXO32
hsa_circ_0003528	hsa_miR_587	C10orf90
hsa_circ_0003528	hsa_miR_587	CNTNAP4
hsa_circ_0003528	hsa_miR_587	IL22RA2
hsa_circ_0003528	hsa_miR_587	CYBRD1
hsa_circ_0003528	hsa_miR_587	SCIN
hsa_circ_0003528	hsa_miR_587	ZNF106
hsa_circ_0003528	hsa_miR_587	ZBTB8B
hsa_circ_0003528	hsa_miR_587	LAPTM4B
hsa_circ_0003528	hsa_miR_587	CAV3
hsa_circ_0003528	hsa_miR_587	RUNX1T1
hsa_circ_0003528	hsa_miR_587	SLC44A5
hsa_circ_0003528	hsa_miR_587	DBF4
hsa_circ_0003528	hsa_miR_587	ITGA8
hsa_circ_0003528	hsa_miR_587	MDFI
hsa_circ_0003528	hsa_miR_587	DPP10
hsa_circ_0003528	hsa_miR_587	CTNNAL1
hsa_circ_0003528	hsa_miR_587	GAGE1
hsa_circ_0003528	hsa_miR_587	ROR1
hsa_circ_0003528	hsa_miR_587	GIPC2
hsa_circ_0003528	hsa_miR_587	PTHLH
hsa_circ_0003528	hsa_miR_587	OTP
hsa_circ_0003528	hsa_miR_587	TCTEX1D1
hsa_circ_0003528	hsa_miR_587	PDZK1IP1
hsa_circ_0003528	hsa_miR_587	KRT222
hsa_circ_0003528	hsa_miR_587	RAD51AP1
hsa_circ_0003528	hsa_miR_587	RORA
hsa_circ_0003528	hsa_miR_587	FGF5
hsa_circ_0003528	hsa_miR_587	TMTC1
hsa_circ_0003528	hsa_miR_587	DPP4
hsa_circ_0003528	hsa_miR_587	SHISA9
hsa_circ_0003528	hsa_miR_587	PCSK6
hsa_circ_0003528	hsa_miR_587	PSG11
hsa_circ_0003528	hsa_miR_587	BEX5
hsa_circ_0003528	hsa_miR_587	COL4A4
hsa_circ_0003528	hsa_miR_587	PCDH9
hsa_circ_0003528	hsa_miR_587	ZNF486
hsa_circ_0003528	hsa_miR_587	EPHA5
hsa_circ_0003528	hsa_miR_587	EPHA8
hsa_circ_0003528	hsa_miR_587	IL17RD
hsa_circ_0003528	hsa_miR_587	SPDYA
hsa_circ_0003528	hsa_miR_587	FBXL19
hsa_circ_0003528	hsa_miR_587	OTOR
hsa_circ_0003528	hsa_miR_587	NKD1
hsa_circ_0003528	hsa_miR_587	ZEB1
hsa_circ_0003528	hsa_miR_587	NMNAT2
hsa_circ_0003528	hsa_miR_587	SGMS2
hsa_circ_0003528	hsa_miR_587	DCX
hsa_circ_0003528	hsa_miR_587	ADAMTS19
hsa_circ_0003528	hsa_miR_587	PSG2
hsa_circ_0003528	hsa_miR_587	SNX31
hsa_circ_0003528	hsa_miR_587	CCND2

hsa_circ_0003528	hsa_miR_587	ICA1
hsa_circ_0003528	hsa_miR_587	FAR2
hsa_circ_0003528	hsa_miR_587	NIPAL1
hsa_circ_0003528	hsa_miR_587	CHL1
hsa_circ_0003528	hsa_miR_587	GLYATL1
hsa_circ_0003528	hsa_miR_587	ABCA12
hsa_circ_0003528	hsa_miR_587	DDO
hsa_circ_0003528	hsa_miR_587	ST8SIA3
hsa_circ_0003528	hsa_miR_587	MFSD9
hsa_circ_0003528	hsa_miR_587	SYT16
hsa_circ_0003528	hsa_miR_587	ATP6V1C2
hsa_circ_0003528	hsa_miR_587	XRCC2
hsa_circ_0003528	hsa_miR_587	MSX2
hsa_circ_0003528	hsa_miR_587	PMAIP1
hsa_circ_0003528	hsa_miR_587	FRMD3
hsa_circ_0003528	hsa_miR_587	WDHD1
hsa_circ_0003528	hsa_miR_587	CP
hsa_circ_0003528	hsa_miR_587	SLC14A1
hsa_circ_0003528	hsa_miR_587	RIC3
hsa_circ_0003528	hsa_miR_587	STK32B
hsa_circ_0003528	hsa_miR_587	NTS
hsa_circ_0003528	hsa_miR_587	EMP2
hsa_circ_0003528	hsa_miR_587	PABPC3
hsa_circ_0003528	hsa_miR_587	FZD10
hsa_circ_0003528	hsa_miR_587	SOCS2
hsa_circ_0003528	hsa_miR_587	NOVA2
hsa_circ_0003528	hsa_miR_587	ECT2
hsa_circ_0003528	hsa_miR_587	FRMD4B
hsa_circ_0003528	hsa_miR_587	RDH10
hsa_circ_0003528	hsa_miR_587	TNS1
hsa_circ_0003528	hsa_miR_587	SCN1A
hsa_circ_0003528	hsa_miR_587	OSR2
hsa_circ_0003528	hsa_miR_587	NTM
hsa_circ_0003528	hsa_miR_587	DOCK8
hsa_circ_0003528	hsa_miR_587	SFRP4
hsa_circ_0003528	hsa_miR_587	PAX9
hsa_circ_0003528	hsa_miR_587	SPOCK3
hsa_circ_0003528	hsa_miR_587	TRPV1
hsa_circ_0003528	hsa_miR_587	RASSF2
hsa_circ_0003528	hsa_miR_587	CKAP2L
hsa_circ_0003528	hsa_miR_587	NAALADL2
hsa_circ_0003528	hsa_miR_587	FILIP1
hsa_circ_0003528	hsa_miR_587	DRAM1
hsa_circ_0003528	hsa_miR_587	RBMS3
hsa_circ_0003528	hsa_miR_587	TMEM178B
hsa_circ_0003528	hsa_miR_587	TMPRSS15
hsa_circ_0003528	hsa_miR_587	FRG2
hsa_circ_0003528	hsa_miR_587	PRG4
hsa_circ_0003528	hsa_miR_587	MOCS1
hsa_circ_0003528	hsa_miR_587	HLTF
hsa_circ_0003528	hsa_miR_587	STON1-GTF2A1L
hsa_circ_0003528	hsa_miR_587	DMC1
hsa_circ_0003528	hsa_miR_587	SLC7A14
hsa_circ_0003528	hsa_miR_587	IQUB
hsa_circ_0003528	hsa_miR_587	ADAM8
hsa_circ_0003528	hsa_miR_587	TENM1
hsa_circ_0003528	hsa_miR_587	GLDN
hsa_circ_0003528	hsa_miR_587	FRG2C

hsa_circ_0003528	hsa_miR_587	ARRB1
hsa_circ_0003528	hsa_miR_587	SLCO1A2
hsa_circ_0003528	hsa_miR_587	CHST6
hsa_circ_0003528	hsa_miR_587	ODAM
hsa_circ_0003528	hsa_miR_587	EPHB2
hsa_circ_0003528	hsa_miR_587	PPARGC1B
hsa_circ_0003528	hsa_miR_587	UBASH3B
hsa_circ_0003528	hsa_miR_587	GPR4
hsa_circ_0003528	hsa_miR_587	VSX2
hsa_circ_0003528	hsa_miR_587	CLVS2
hsa_circ_0003528	hsa_miR_587	MDGA2
hsa_circ_0003528	hsa_miR_587	PLAGL1
hsa_circ_0003528	hsa_miR_587	STYK1
hsa_circ_0003528	hsa_miR_587	VCAN
hsa_circ_0003528	hsa_miR_587	AK7
hsa_circ_0003528	hsa_miR_587	C20orf197
hsa_circ_0003528	hsa_miR_587	DENND2C
hsa_circ_0003528	hsa_miR_587	PCSK2
hsa_circ_0003528	hsa_miR_587	ASPHD2
hsa_circ_0003528	hsa_miR_587	SLC25A2
hsa_circ_0003528	hsa_miR_587	UST
hsa_circ_0003528	hsa_miR_587	UNC13A
hsa_circ_0003528	hsa_miR_587	FAM169A
hsa_circ_0003528	hsa_miR_587	SPIN2A
hsa_circ_0003528	hsa_miR_587	PCDH7
hsa_circ_0003528	hsa_miR_587	SLC25A15
hsa_circ_0003528	hsa_miR_892b	MAPK10
hsa_circ_0003528	hsa_miR_892b	GPR146
hsa_circ_0003528	hsa_miR_892b	SYT1
hsa_circ_0003528	hsa_miR_892b	ETS1
hsa_circ_0003528	hsa_miR_892b	LHFPL1
hsa_circ_0003528	hsa_miR_892b	APLN
hsa_circ_0003528	hsa_miR_892b	KANK2
hsa_circ_0003528	hsa_miR_892b	HOXC6
hsa_circ_0003528	hsa_miR_892b	IGF2BP1
hsa_circ_0003528	hsa_miR_892b	SPRR2A
hsa_circ_0003528	hsa_miR_892b	ERBB4
hsa_circ_0003528	hsa_miR_892b	TIMP3
hsa_circ_0003528	hsa_miR_892b	PDX1
hsa_circ_0003528	hsa_miR_892b	CCL16
hsa_circ_0003528	hsa_miR_892b	PGR
hsa_circ_0003528	hsa_miR_892b	ADCY9
hsa_circ_0003528	hsa_miR_892b	ROR1
hsa_circ_0003528	hsa_miR_892b	CRIM1
hsa_circ_0003528	hsa_miR_892b	CCNB1
hsa_circ_0003528	hsa_miR_892b	KCNMB2
hsa_circ_0003528	hsa_miR_892b	SLC6A15
hsa_circ_0003528	hsa_miR_892b	TMEM74
hsa_circ_0003528	hsa_miR_892b	SH3GL2
hsa_circ_0003528	hsa_miR_892b	CDH7
hsa_circ_0003528	hsa_miR_892b	RDH10
hsa_circ_0003528	hsa_miR_892b	STXBP5L
hsa_circ_0003528	hsa_miR_892b	OMG
hsa_circ_0003528	hsa_miR_892b	PLEKHA8
hsa_circ_0003528	hsa_miR_892b	CACNA1E
hsa_circ_0003528	hsa_miR_892b	PAK3
hsa_circ_0003528	hsa_miR_892b	EPHA5
hsa_circ_0003528	hsa_miR_892b	ACHE

hsa_circ_0003528	hsa_miR_892b	FAT1
hsa_circ_0003528	hsa_miR_892b	UNC13C
hsa_circ_0003528	hsa_miR_892b	PPM1F
hsa_circ_0003528	hsa_miR_892b	IL17RD
hsa_circ_0003528	hsa_miR_892b	TTC22
hsa_circ_0003528	hsa_miR_892b	NT5E
hsa_circ_0003528	hsa_miR_892b	MSI2
hsa_circ_0003528	hsa_miR_892b	WNT9B
hsa_circ_0003528	hsa_miR_892b	IGF2BP3
hsa_circ_0003528	hsa_miR_892b	ST6GALNAC5
hsa_circ_0003528	hsa_miR_892b	TMEM236
hsa_circ_0003528	hsa_miR_892b	HOXD13
hsa_circ_0003528	hsa_miR_892b	RIMS2
hsa_circ_0003528	hsa_miR_892b	LPGAT1
hsa_circ_0003528	hsa_miR_892b	ARHGEF15
hsa_circ_0003528	hsa_miR_892b	KIF1C
hsa_circ_0003528	hsa_miR_892b	TCF21
hsa_circ_0003528	hsa_miR_892b	HEG1
hsa_circ_0003528	hsa_miR_892b	PTPRT
hsa_circ_0003528	hsa_miR_892b	DHH
hsa_circ_0003528	hsa_miR_892b	LST1
hsa_circ_0003528	hsa_miR_892b	GSR
hsa_circ_0003528	hsa_miR_892b	ETV1
hsa_circ_0003528	hsa_miR_892b	ABCB9
hsa_circ_0003528	hsa_miR_892b	RUNX1T1
hsa_circ_0003528	hsa_miR_892b	IDH2
hsa_circ_0003528	hsa_miR_892b	PCDHGB7
hsa_circ_0003528	hsa_miR_892b	PCDHGA12
hsa_circ_0003528	hsa_miR_892b	PCDHGA1
hsa_circ_0003528	hsa_miR_892b	CREG2
hsa_circ_0003528	hsa_miR_892b	PCDHGA8
hsa_circ_0003528	hsa_miR_892b	PCDHGC3
hsa_circ_0003528	hsa_miR_892b	PCDHA8
hsa_circ_0003528	hsa_miR_892b	ADAM28
hsa_circ_0003528	hsa_miR_892b	PCDHA5
hsa_circ_0003528	hsa_miR_892b	PCDHA6
hsa_circ_0003528	hsa_miR_892b	PCDHA11
hsa_circ_0003528	hsa_miR_892b	PCDHA9
hsa_circ_0003528	hsa_miR_892b	PCDHA4
hsa_circ_0003528	hsa_miR_892b	PCDHA1
hsa_circ_0003528	hsa_miR_892b	KIF5C
hsa_circ_0003528	hsa_miR_892b	PCDHA7
hsa_circ_0003528	hsa_miR_892b	PCDHA12
hsa_circ_0003528	hsa_miR_892b	FGD6
hsa_circ_0003528	hsa_miR_892b	BTG2
hsa_circ_0003528	hsa_miR_892b	OLFML1
hsa_circ_0003528	hsa_miR_892b	CAPN6
hsa_circ_0003528	hsa_miR_892b	KRT86
hsa_circ_0003528	hsa_miR_892b	SASS6
hsa_circ_0003528	hsa_miR_892b	PIK3R1
hsa_circ_0003528	hsa_miR_892b	RSPO2
hsa_circ_0003528	hsa_miR_892b	NTNG1
hsa_circ_0003528	hsa_miR_892b	IGSF1
hsa_circ_0003528	hsa_miR_892b	HPRT1
hsa_circ_0003528	hsa_miR_892b	CABLES2
hsa_circ_0003528	hsa_miR_892b	SLC26A1
hsa_circ_0003528	hsa_miR_668	ID4
hsa_circ_0003528	hsa_miR_668	TOX3

hsa_circ_0003528	hsa_miR_668	AK4
hsa_circ_0003528	hsa_miR_668	LMOD3
hsa_circ_0003528	hsa_miR_668	NEGR1
hsa_circ_0003528	hsa_miR_668	SCN2B
hsa_circ_0003528	hsa_miR_668	C1orf189
hsa_circ_0003528	hsa_miR_668	FUT8
hsa_circ_0003528	hsa_miR_668	CDH2
hsa_circ_0003528	hsa_miR_1253	SOBP
hsa_circ_0003528	hsa_miR_1253	COL11A1
hsa_circ_0003528	hsa_miR_1253	SRL
hsa_circ_0003528	hsa_miR_1253	FBN2
hsa_circ_0003528	hsa_miR_1253	FCRL4
hsa_circ_0003528	hsa_miR_1253	LCOR
hsa_circ_0003528	hsa_miR_1253	KRT6B
hsa_circ_0003528	hsa_miR_1253	TCF24
hsa_circ_0003528	hsa_miR_1253	SMKR1
hsa_circ_0003528	hsa_miR_1253	HYDIN
hsa_circ_0003528	hsa_miR_1253	MSX2
hsa_circ_0003528	hsa_miR_1253	SPC25
hsa_circ_0003528	hsa_miR_1253	RBPJL
hsa_circ_0003528	hsa_miR_1253	TMEM178B
hsa_circ_0003528	hsa_miR_1253	OPRK1
hsa_circ_0003528	hsa_miR_1253	CDC20B
hsa_circ_0003528	hsa_miR_1253	PPM1H
hsa_circ_0003528	hsa_miR_1253	ACRV1
hsa_circ_0003528	hsa_miR_1253	ECT2
hsa_circ_0003528	hsa_miR_1253	SCN7A
hsa_circ_0003528	hsa_miR_1253	TAS2R13
hsa_circ_0003528	hsa_miR_1253	PTGER4
hsa_circ_0003528	hsa_miR_1253	ZBTB20
hsa_circ_0003528	hsa_miR_1253	S100A7A
hsa_circ_0003528	hsa_miR_1253	FAM124B
hsa_circ_0003528	hsa_miR_1253	PPM1N
hsa_circ_0003528	hsa_miR_1253	CACNB4
hsa_circ_0003528	hsa_miR_1253	FGL1
hsa_circ_0003528	hsa_miR_1253	IGSF11
hsa_circ_0003528	hsa_miR_1253	SLC1A7
hsa_circ_0003528	hsa_miR_1253	UGT2A3
hsa_circ_0003528	hsa_miR_1253	ENTPD7
hsa_circ_0003528	hsa_miR_1253	CHODL
hsa_circ_0003528	hsa_miR_1253	BHMT2
hsa_circ_0003528	hsa_miR_1253	ADCY10
hsa_circ_0003528	hsa_miR_1253	TRIM58
hsa_circ_0003528	hsa_miR_1253	NOVA2
hsa_circ_0003528	hsa_miR_1253	PDE11A
hsa_circ_0003528	hsa_miR_1253	GABRA4
hsa_circ_0003528	hsa_miR_1253	EPN3
hsa_circ_0003528	hsa_miR_1253	NHSL1
hsa_circ_0003528	hsa_miR_1253	CDX1
hsa_circ_0003528	hsa_miR_1253	FGF14
hsa_circ_0003528	hsa_miR_1253	NKAIN1
hsa_circ_0003528	hsa_miR_1253	VSTM4
hsa_circ_0003528	hsa_miR_1253	PDX1
hsa_circ_0003528	hsa_miR_1253	TOP2A
hsa_circ_0003528	hsa_miR_1253	QKI
hsa_circ_0003528	hsa_miR_1253	SGPP2
hsa_circ_0003528	hsa_miR_1253	EPPIN-WFDC6
hsa_circ_0003528	hsa_miR_1253	PGM2L1

hsa_circ_0003528	hsa_miR_1253	SCN3B
hsa_circ_0003528	hsa_miR_1253	LRAT
hsa_circ_0003528	hsa_miR_1253	NEXN
hsa_circ_0003528	hsa_miR_1253	KIF5A
hsa_circ_0003528	hsa_miR_1253	SLC24A4
hsa_circ_0003528	hsa_miR_1253	SPRR2B
hsa_circ_0003528	hsa_miR_1253	PAX7
hsa_circ_0003528	hsa_miR_1253	ANXA3
hsa_circ_0003528	hsa_miR_1253	SPRR2A
hsa_circ_0003528	hsa_miR_1253	ADCY9
hsa_circ_0003528	hsa_miR_1253	HOXA1
hsa_circ_0003528	hsa_miR_1253	FLT4
hsa_circ_0003528	hsa_miR_1253	TEX14
hsa_circ_0003528	hsa_miR_1253	PLEKHA8
hsa_circ_0003528	hsa_miR_1253	LYZL2
hsa_circ_0003528	hsa_miR_1253	C1orf21
hsa_circ_0003528	hsa_miR_1253	SCAI
hsa_circ_0003528	hsa_miR_1253	GCNT4
hsa_circ_0003528	hsa_miR_1253	MFAP3L
hsa_circ_0003528	hsa_miR_1253	SIAH3
hsa_circ_0003528	hsa_miR_1253	KIAA1522
hsa_circ_0003528	hsa_miR_1253	SPRY4
hsa_circ_0003528	hsa_miR_1253	GPC5
hsa_circ_0003528	hsa_miR_1253	LPGAT1
hsa_circ_0003528	hsa_miR_1253	KIF3C
hsa_circ_0003528	hsa_miR_1253	LGALSL
hsa_circ_0003528	hsa_miR_1253	SH2D4B
hsa_circ_0003528	hsa_miR_1253	TRIM67
hsa_circ_0003528	hsa_miR_1253	ASB11
hsa_circ_0003528	hsa_miR_1253	GFRA2
hsa_circ_0003528	hsa_miR_1253	GPD1
hsa_circ_0003528	hsa_miR_1253	CD33
hsa_circ_0003528	hsa_miR_1253	SLA
hsa_circ_0003528	hsa_miR_1253	KCTD12
hsa_circ_0003528	hsa_miR_1253	BACE2
hsa_circ_0003528	hsa_miR_1253	RAD51AP1
hsa_circ_0003528	hsa_miR_1253	ROBO2
hsa_circ_0003528	hsa_miR_1253	FOS
hsa_circ_0003528	hsa_miR_1253	TM4SF4
hsa_circ_0003528	hsa_miR_1253	CLEC1A
hsa_circ_0003528	hsa_miR_1253	TMTC4
hsa_circ_0003528	hsa_miR_1253	GPM6A
hsa_circ_0003528	hsa_miR_1253	UNC45B
hsa_circ_0003528	hsa_miR_1253	TSPAN5
hsa_circ_0003528	hsa_miR_1253	JPH4
hsa_circ_0003528	hsa_miR_1253	FAM83A
hsa_circ_0003528	hsa_miR_1253	KLHDC8A
hsa_circ_0003528	hsa_miR_1253	CPLX4
hsa_circ_0003528	hsa_miR_1253	CDC25C
hsa_circ_0003528	hsa_miR_1253	MEFV
hsa_circ_0003528	hsa_miR_1253	SEMA7A
hsa_circ_0003528	hsa_miR_1253	RASD1
hsa_circ_0003528	hsa_miR_1253	HAP1
hsa_circ_0003528	hsa_miR_1253	LPXN
hsa_circ_0003528	hsa_miR_1253	PAX6
hsa_circ_0003528	hsa_miR_1253	GRIN2C
hsa_circ_0003528	hsa_miR_1253	ANKS1A
hsa_circ_0003528	hsa_miR_1253	CDSN

hsa_circ_0003528	hsa_miR_1253	EZH2
hsa_circ_0003528	hsa_miR_1253	ZNF560
hsa_circ_0003528	hsa_miR_1253	GRIK4
hsa_circ_0003528	hsa_miR_1253	RBMS2
hsa_circ_0003528	hsa_miR_1253	PCSK2
hsa_circ_0003528	hsa_miR_1253	CCBE1
hsa_circ_0003528	hsa_miR_1253	DMBX1
hsa_circ_0003528	hsa_miR_1253	LYPD6
hsa_circ_0003528	hsa_miR_1253	EFR3B
hsa_circ_0003528	hsa_miR_1253	CLIC6
hsa_circ_0003528	hsa_miR_1253	ONECUT2
hsa_circ_0003528	hsa_miR_1253	WDR76
hsa_circ_0003528	hsa_miR_1253	SOX4
hsa_circ_0003528	hsa_miR_1253	ARHGAP11A
hsa_circ_0003528	hsa_miR_1253	MCF2
hsa_circ_0003528	hsa_miR_1253	SYT5
hsa_circ_0003528	hsa_miR_1253	KCNT2
hsa_circ_0003528	hsa_miR_1253	LMNB1
hsa_circ_0003528	hsa_miR_1253	PXMP4
hsa_circ_0003528	hsa_miR_1253	FXYD6
hsa_circ_0003528	hsa_miR_1253	LYPD1
hsa_circ_0003528	hsa_miR_1253	POTEF
hsa_circ_0003528	hsa_miR_1253	PAX3
hsa_circ_0003528	hsa_miR_1253	GSTM5
hsa_circ_0003528	hsa_miR_1253	RAB3B
hsa_circ_0003528	hsa_miR_1253	SCN5A
hsa_circ_0003528	hsa_miR_1253	HPX
hsa_circ_0003528	hsa_miR_1253	HOXA4
hsa_circ_0003528	hsa_miR_1253	CXCL6
hsa_circ_0003528	hsa_miR_1253	ANKRD34A
hsa_circ_0003528	hsa_miR_1253	ARHGEF39
hsa_circ_0003528	hsa_miR_1253	ERBB4
hsa_circ_0003528	hsa_miR_1253	CCT3
hsa_circ_0003528	hsa_miR_1253	DCC
hsa_circ_0003528	hsa_miR_1253	FRY
hsa_circ_0003528	hsa_miR_1253	SALL3
hsa_circ_0003528	hsa_miR_1253	ARHGAP39
hsa_circ_0003528	hsa_miR_1253	MMP20
hsa_circ_0003528	hsa_miR_1253	SLC35F2
hsa_circ_0003528	hsa_miR_1253	PHGR1
hsa_circ_0003528	hsa_miR_1253	KRT13
hsa_circ_0003528	hsa_miR_1253	TSPYL6
hsa_circ_0003528	hsa_miR_1253	LINGO1
hsa_circ_0003528	hsa_miR_1253	GFRA1
hsa_circ_0003528	hsa_miR_1253	SIX3
hsa_circ_0003528	hsa_miR_1253	NFASC
hsa_circ_0003528	hsa_miR_1253	AMOTL1
hsa_circ_0003528	hsa_miR_1253	SLC16A1
hsa_circ_0003528	hsa_miR_1253	ANKRD29
hsa_circ_0003528	hsa_miR_1253	PYCR1
hsa_circ_0003528	hsa_miR_1253	ST8SIA6
hsa_circ_0003528	hsa_miR_1253	FCAR
hsa_circ_0003528	hsa_miR_1253	GUCY1A2
hsa_circ_0003528	hsa_miR_1253	KRT80
hsa_circ_0003528	hsa_miR_1253	THRB
hsa_circ_0003528	hsa_miR_1253	CDHR3
hsa_circ_0003528	hsa_miR_1253	REEP1
hsa_circ_0003528	hsa_miR_1253	CCT5

hsa_circ_0003528	hsa_miR_1253	HORMAD1
hsa_circ_0003528	hsa_miR_1253	TMSB15A
hsa_circ_0003528	hsa_miR_1253	RANBP3L
hsa_circ_0003528	hsa_miR_1253	LRRC66
hsa_circ_0003528	hsa_miR_1253	MMRN2
hsa_circ_0003528	hsa_miR_1253	DEPDC1
hsa_circ_0003528	hsa_miR_1253	SERPINB13
hsa_circ_0003528	hsa_miR_1253	TMEM47
hsa_circ_0003528	hsa_miR_1253	CYP7A1
hsa_circ_0003528	hsa_miR_1253	SLC16A7
hsa_circ_0003528	hsa_miR_1253	CADM1
hsa_circ_0003528	hsa_miR_1253	APLN
hsa_circ_0003528	hsa_miR_1253	DGKI
hsa_circ_0003528	hsa_miR_1253	PLA2G5
hsa_circ_0003528	hsa_miR_1253	PTPRM
hsa_circ_0003528	hsa_miR_1253	ANLN
hsa_circ_0003528	hsa_miR_1253	KCNE1
hsa_circ_0003528	hsa_miR_1253	RNF212
hsa_circ_0003528	hsa_miR_1253	CAPN5
hsa_circ_0003528	hsa_miR_1253	PEX5L
hsa_circ_0003528	hsa_miR_1253	EGR3
hsa_circ_0003528	hsa_miR_1253	OR51E1
hsa_circ_0003528	hsa_miR_1253	PBLD
hsa_circ_0003528	hsa_miR_1253	ELAVL4
hsa_circ_0003528	hsa_miR_1253	MYRF
hsa_circ_0003528	hsa_miR_1253	MPPED1
hsa_circ_0003528	hsa_miR_1253	AQP11
hsa_circ_0003528	hsa_miR_1253	TCF4
hsa_circ_0003528	hsa_miR_1253	COLEC12
hsa_circ_0003528	hsa_miR_1253	DNMT3B
hsa_circ_0003528	hsa_miR_1253	ARHGEF10
hsa_circ_0003528	hsa_miR_1253	FKBP4
hsa_circ_0003528	hsa_miR_1253	DSG3
hsa_circ_0003528	hsa_miR_1253	AWAT2
hsa_circ_0003528	hsa_miR_1253	RIMBP2
hsa_circ_0003528	hsa_miR_1253	SASS6
hsa_circ_0003528	hsa_miR_1253	SPIRE2
hsa_circ_0003528	hsa_miR_1253	SLC9A7
hsa_circ_0003528	hsa_miR_1253	PDE1C
hsa_circ_0003528	hsa_miR_1253	JDP2
hsa_circ_0003528	hsa_miR_1253	SLC25A21
hsa_circ_0003528	hsa_miR_1253	LGI2
hsa_circ_0003528	hsa_miR_1253	GPR82
hsa_circ_0003528	hsa_miR_1253	PCDH9
hsa_circ_0003528	hsa_miR_1253	ST8SIA3
hsa_circ_0003528	hsa_miR_1253	GATA6
hsa_circ_0003528	hsa_miR_1253	DOK6
hsa_circ_0003528	hsa_miR_1253	LYPD3
hsa_circ_0003528	hsa_miR_1253	TM6SF1
hsa_circ_0003528	hsa_miR_1253	EYA4
hsa_circ_0003528	hsa_miR_1253	C1orf226
hsa_circ_0003528	hsa_miR_1253	TMEM178A
hsa_circ_0003528	hsa_miR_1253	KCNH1
hsa_circ_0003528	hsa_miR_1253	CPD
hsa_circ_0003528	hsa_miR_1253	NDRG4
hsa_circ_0003528	hsa_miR_1253	RPTN
hsa_circ_0003528	hsa_miR_1253	AMER2
hsa_circ_0003528	hsa_miR_1253	FAM135B

hsa_circ_0003528	hsa_miR_370	NR4A3
hsa_circ_0003528	hsa_miR_370	PMAIP1
hsa_circ_0003528	hsa_miR_370	PLEKHG4B
hsa_circ_0003528	hsa_miR_370	TPO
hsa_circ_0003528	hsa_miR_370	CST2
hsa_circ_0003528	hsa_miR_370	NKAPL
hsa_circ_0003528	hsa_miR_370	ERVV-2
hsa_circ_0003528	hsa_miR_370	PRTFDC1
hsa_circ_0003528	hsa_miR_370	CASKIN1
hsa_circ_0003528	hsa_miR_370	F2RL2
hsa_circ_0003528	hsa_miR_370	HPS5
hsa_circ_0003528	hsa_miR_370	TNS1
hsa_circ_0003528	hsa_miR_370	APOL3
hsa_circ_0003528	hsa_miR_370	GLYATL1
hsa_circ_0003528	hsa_miR_370	ZNF556
hsa_circ_0003528	hsa_miR_370	SPTSSB
hsa_circ_0003528	hsa_miR_370	N4BP2L1
hsa_circ_0003528	hsa_miR_370	FBLN5
hsa_circ_0003528	hsa_miR_370	RAB15
hsa_circ_0003528	hsa_miR_370	FCGR3A
hsa_circ_0003528	hsa_miR_370	FCGR3B
hsa_circ_0003528	hsa_miR_370	KCNK3
hsa_circ_0003528	hsa_miR_370	OSCAR
hsa_circ_0003528	hsa_miR_370	EGLN3
hsa_circ_0003528	hsa_miR_370	ATAT1
hsa_circ_0003528	hsa_miR_370	WNT10B
hsa_circ_0003528	hsa_miR_370	HMGA2
hsa_circ_0003528	hsa_miR_370	PRND
hsa_circ_0003528	hsa_miR_370	CCNE2
hsa_circ_0003528	hsa_miR_370	RUNX1T1
hsa_circ_0003528	hsa_miR_370	SPDYE1
hsa_circ_0003528	hsa_miR_370	CNPY1
hsa_circ_0003528	hsa_miR_370	RBBP8NL
hsa_circ_0003528	hsa_miR_370	LAMC3
hsa_circ_0003528	hsa_miR_370	CRLF1
hsa_circ_0003528	hsa_miR_370	FCRL2
hsa_circ_0003528	hsa_miR_370	CASQ2
hsa_circ_0003528	hsa_miR_370	NKAIN1
hsa_circ_0003528	hsa_miR_370	C17orf58
hsa_circ_0003528	hsa_miR_370	CES3
hsa_circ_0003528	hsa_miR_370	PAQR4
hsa_circ_0003528	hsa_miR_370	SEZ6
hsa_circ_0003528	hsa_miR_370	KCNK10
hsa_circ_0003528	hsa_miR_370	SLC9A4
hsa_circ_0003528	hsa_miR_370	SLC25A10
hsa_circ_0003528	hsa_miR_370	PPM1F
hsa_circ_0003528	hsa_miR_370	CA12
hsa_circ_0003528	hsa_miR_495	CADM1
hsa_circ_0003528	hsa_miR_495	DDIT4
hsa_circ_0003528	hsa_miR_495	BDNF
hsa_circ_0003528	hsa_miR_495	CELF2
hsa_circ_0003528	hsa_miR_495	THBS2
hsa_circ_0003528	hsa_miR_495	NTNG1
hsa_circ_0003528	hsa_miR_495	ATP2B2
hsa_circ_0003528	hsa_miR_495	TCF4
hsa_circ_0003528	hsa_miR_495	ETS1
hsa_circ_0003528	hsa_miR_495	PAQR9
hsa_circ_0003528	hsa_miR_495	CYP26B1

hsa_circ_0003528	hsa_miR_1248	NXPH1
hsa_circ_0003528	hsa_miR_1248	AHSG
hsa_circ_0003528	hsa_miR_1248	BCL6B
hsa_circ_0003528	hsa_miR_1248	ETS1
hsa_circ_0003528	hsa_miR_1248	RIMS2
hsa_circ_0003528	hsa_miR_1248	MCIDAS
hsa_circ_0003528	hsa_miR_1248	NEGR1
hsa_circ_0003528	hsa_miR_1248	ADAM28
hsa_circ_0003528	hsa_miR_1248	PRICKLE2
hsa_circ_0003528	hsa_miR_1248	CHRM2
hsa_circ_0003528	hsa_miR_1248	DLC1
hsa_circ_0003528	hsa_miR_1248	HECW1
hsa_circ_0003528	hsa_miR_1248	FBXO32
hsa_circ_0003528	hsa_miR_1248	ONECUT2
hsa_circ_0003528	hsa_miR_1248	RORA
hsa_circ_0003528	hsa_miR_1248	RUNX2
hsa_circ_0003528	hsa_miR_1248	IL17RD
hsa_circ_0003528	hsa_miR_1248	LRRC8C
hsa_circ_0003528	hsa_miR_1248	TFAP2B
hsa_circ_0003528	hsa_miR_1248	F5
hsa_circ_0003528	hsa_miR_1248	HYAL4
hsa_circ_0003528	hsa_miR_1248	ADAMTS1
hsa_circ_0003528	hsa_miR_1248	LRAT
hsa_circ_0003528	hsa_miR_1248	FA2H
hsa_circ_0003528	hsa_miR_1248	ACAN
hsa_circ_0003528	hsa_miR_1248	GRIA1
hsa_circ_0003528	hsa_miR_1248	SFRP4
hsa_circ_0003528	hsa_miR_1248	MS4A2
hsa_circ_0003528	hsa_miR_1248	KIAA0408
hsa_circ_0003528	hsa_miR_1248	FAM13C
hsa_circ_0003528	hsa_miR_1248	ADAMTS16
hsa_circ_0003528	hsa_miR_1248	ABCA10
hsa_circ_0003528	hsa_miR_1248	NOVA1
hsa_circ_0003528	hsa_miR_1248	COL24A1
hsa_circ_0003528	hsa_miR_1248	TYMS
hsa_circ_0003528	hsa_miR_1248	TXNRD1
hsa_circ_0003528	hsa_miR_1248	KLF6
hsa_circ_0003528	hsa_miR_1248	CBFA2T3
hsa_circ_0003528	hsa_miR_1248	GALNT4
hsa_circ_0003528	hsa_miR_1248	GPR26
hsa_circ_0003528	hsa_miR_1248	PADI3
hsa_circ_0003528	hsa_miR_1248	CNTNAP2
hsa_circ_0003528	hsa_miR_1248	AQP10
hsa_circ_0003528	hsa_miR_1248	SNTG1
hsa_circ_0003528	hsa_miR_1248	HHLA2
hsa_circ_0003528	hsa_miR_1248	ALX4
hsa_circ_0003528	hsa_miR_1248	ADAM7
hsa_circ_0003528	hsa_miR_1248	CDKN2B
hsa_circ_0003528	hsa_miR_1248	MC2R
hsa_circ_0003528	hsa_miR_1248	DLX1
hsa_circ_0003528	hsa_miR_1248	CREG2
hsa_circ_0003528	hsa_miR_1248	FGF2
hsa_circ_0003528	hsa_miR_1248	ERBB4
hsa_circ_0003528	hsa_miR_1248	ACSM2A
hsa_circ_0003528	hsa_miR_1248	EPHA5
hsa_circ_0003528	hsa_miR_1248	PRKCE
hsa_circ_0003528	hsa_miR_1248	LMNB1
hsa_circ_0003528	hsa_miR_1248	ELAVL2

hsa_circ_0003528	hsa_miR_1248	LIN28A
hsa_circ_0003528	hsa_miR_1248	NTNG1
hsa_circ_0003528	hsa_miR_1248	SPINT2
hsa_circ_0003528	hsa_miR_1248	CRELD2
hsa_circ_0003528	hsa_miR_1248	HTR2C
hsa_circ_0003528	hsa_miR_1248	CCND2
hsa_circ_0003528	hsa_miR_1248	COL6A3
hsa_circ_0003528	hsa_miR_1248	CACNA1E
hsa_circ_0003528	hsa_miR_1248	ZNF648
hsa_circ_0003528	hsa_miR_1248	HLTF
hsa_circ_0003528	hsa_miR_1248	ZIC4
hsa_circ_0003528	hsa_miR_1248	SLIT3
hsa_circ_0003528	hsa_miR_1248	THEG
hsa_circ_0003528	hsa_miR_1248	SLC39A11
hsa_circ_0003528	hsa_miR_1248	ATP2A1
hsa_circ_0003528	hsa_miR_1248	KDELR2
hsa_circ_0003528	hsa_miR_1248	SLA
hsa_circ_0003528	hsa_miR_1248	LRRC2
hsa_circ_0003528	hsa_miR_1248	GPR88
hsa_circ_0003528	hsa_miR_1248	GFRA1
hsa_circ_0003528	hsa_miR_1248	CACNG8
hsa_circ_0003528	hsa_miR_1248	KIAA1549L
hsa_circ_0003528	hsa_miR_1248	ZNF705A
hsa_circ_0003528	hsa_miR_1248	C18orf63
hsa_circ_0003528	hsa_miR_1248	BMPR2
hsa_circ_0003528	hsa_miR_1248	EGR3
hsa_circ_0003528	hsa_miR_1248	LHFPL3
hsa_circ_0003528	hsa_miR_1248	ATF3
hsa_circ_0003528	hsa_miR_1248	TBX18
hsa_circ_0003528	hsa_miR_1248	XPR1
hsa_circ_0003528	hsa_miR_1248	CTXN3
hsa_circ_0003528	hsa_miR_1248	DIO2
hsa_circ_0003528	hsa_miR_1248	BTG2
hsa_circ_0003528	hsa_miR_1248	TNS1
hsa_circ_0003528	hsa_miR_1248	NFATC1
hsa_circ_0003528	hsa_miR_1248	CCDC137
hsa_circ_0003528	hsa_miR_1248	CTLA4
hsa_circ_0003528	hsa_miR_1248	TSKU
hsa_circ_0003528	hsa_miR_1248	RAB15
hsa_circ_0003528	hsa_miR_1248	C1orf21
hsa_circ_0003528	hsa_miR_1248	VAX2
hsa_circ_0003528	hsa_miR_1248	EPHB2
hsa_circ_0003528	hsa_miR_1248	ADAMTS15
hsa_circ_0003528	hsa_miR_1248	DSCC1
hsa_circ_0003528	hsa_miR_1248	CBR1
hsa_circ_0003528	hsa_miR_1248	LGR4
hsa_circ_0003528	hsa_miR_1248	OLR1
hsa_circ_0003528	hsa_miR_1248	DMP1
hsa_circ_0003528	hsa_miR_1248	LEPR
hsa_circ_0003528	hsa_miR_1248	SLC26A3
hsa_circ_0003528	hsa_miR_1248	CXCL6
hsa_circ_0003528	hsa_miR_1248	ZNF367
hsa_circ_0003528	hsa_miR_1248	CYBB
hsa_circ_0003528	hsa_miR_1248	SVOP
hsa_circ_0003528	hsa_miR_1248	KNTC1
hsa_circ_0003528	hsa_miR_1248	STARD13
hsa_circ_0003528	hsa_miR_1248	TMEM178B
hsa_circ_0003528	hsa_miR_1248	KLF10

hsa_circ_0003528	hsa_miR_1248	PPIL1
hsa_circ_0003528	hsa_miR_1248	ARHGAP36
hsa_circ_0003528	hsa_miR_1248	EN2
hsa_circ_0003528	hsa_miR_1248	FIBIN
hsa_circ_0003528	hsa_miR_1248	SOX4
hsa_circ_0003528	hsa_miR_1248	PIK3R5
hsa_circ_0003528	hsa_miR_1248	COL1A2
hsa_circ_0003528	hsa_miR_1248	SMAD7
hsa_circ_0003528	hsa_miR_1248	AGMO
hsa_circ_0003528	hsa_miR_1248	ANP32E
hsa_circ_0003528	hsa_miR_1248	ZNF331
hsa_circ_0003528	hsa_miR_1248	ZBTB20
hsa_circ_0003528	hsa_miR_1248	ADARB1
hsa_circ_0003528	hsa_miR_1248	SYNPO2
hsa_circ_0003528	hsa_miR_1248	RBFOX1
hsa_circ_0003528	hsa_miR_1248	AGTR1
hsa_circ_0003528	hsa_miR_1248	SLC17A4
hsa_circ_0003528	hsa_miR_1248	RAPGEF4
hsa_circ_0003528	hsa_miR_1248	MAP10
hsa_circ_0003528	hsa_miR_1248	ABLIM3
hsa_circ_0003528	hsa_miR_1248	C1orf115
hsa_circ_0003528	hsa_miR_1248	FOXF2
hsa_circ_0003528	hsa_miR_1248	RNF157
hsa_circ_0003528	hsa_miR_1248	CALCB
hsa_circ_0003528	hsa_miR_1248	CACNA1B
hsa_circ_0003528	hsa_miR_1248	TMEM182
hsa_circ_0003528	hsa_miR_1248	QPRT
hsa_circ_0003528	hsa_miR_1248	C6orf141
hsa_circ_0003528	hsa_miR_1248	WNK3
hsa_circ_0003528	hsa_miR_1248	NHS
hsa_circ_0003528	hsa_miR_1248	CD300E
hsa_circ_0003528	hsa_miR_1248	LSAMP
hsa_circ_0003528	hsa_miR_1248	MAP2K6
hsa_circ_0003528	hsa_miR_1248	VWC2
hsa_circ_0003528	hsa_miR_1248	LYPD6
hsa_circ_0003528	hsa_miR_1248	KIF4A
hsa_circ_0003528	hsa_miR_1248	FGFR2
hsa_circ_0003528	hsa_miR_1248	PAX3
hsa_circ_0003528	hsa_miR_1248	KIF4B
hsa_circ_0003528	hsa_miR_1248	MUC21
hsa_circ_0003528	hsa_miR_324-5p	MGAT3
hsa_circ_0003528	hsa_miR_324-5p	PCYT1B
hsa_circ_0003528	hsa_miR_324-5p	CAMKV
hsa_circ_0003528	hsa_miR_324-5p	DCX
hsa_circ_0003528	hsa_miR_324-5p	LMX1A
hsa_circ_0003528	hsa_miR_324-5p	APOLD1
hsa_circ_0003528	hsa_miR_324-5p	ARHGAP36
hsa_circ_0003528	hsa_miR_324-5p	PTPRD
hsa_circ_0003528	hsa_miR_324-5p	MMP19
hsa_circ_0003528	hsa_miR_324-5p	EYA4
hsa_circ_0003528	hsa_miR_548I	S1PR1
hsa_circ_0003528	hsa_miR_330-3p	GNRHR
hsa_circ_0003528	hsa_miR_330-3p	RCAN1
hsa_circ_0003528	hsa_miR_330-3p	PTGFR
hsa_circ_0003528	hsa_miR_330-3p	SOSTDC1
hsa_circ_0003528	hsa_miR_330-3p	TAC3
hsa_circ_0003528	hsa_miR_330-3p	RSPO2
hsa_circ_0003528	hsa_miR_330-3p	KLF10

hsa_circ_0003528	hsa_miR_330-3p	ADAM12
hsa_circ_0003528	hsa_miR_330-3p	CLDN18
hsa_circ_0003528	hsa_miR_330-3p	DLX1
hsa_circ_0003528	hsa_miR_330-3p	UBL3
hsa_circ_0003528	hsa_miR_330-3p	ZNF367
hsa_circ_0003528	hsa_miR_330-3p	RAI2
hsa_circ_0003528	hsa_miR_330-3p	AGTR2
hsa_circ_0003528	hsa_miR_330-3p	GPR37
hsa_circ_0003528	hsa_miR_330-3p	ZNF423
hsa_circ_0003528	hsa_miR_330-3p	PRTFDC1
hsa_circ_0003528	hsa_miR_330-3p	TXNDC17
hsa_circ_0003528	hsa_miR_330-3p	DLX6
hsa_circ_0003528	hsa_miR_330-3p	S100B
hsa_circ_0003528	hsa_miR_330-3p	C1orf115
hsa_circ_0003528	hsa_miR_330-3p	ITM2C
hsa_circ_0003528	hsa_miR_330-3p	TDRKH
hsa_circ_0003528	hsa_miR_330-3p	USP2
hsa_circ_0003528	hsa_miR_330-3p	ALAS2
hsa_circ_0003528	hsa_miR_330-3p	KLHL32
hsa_circ_0003528	hsa_miR_330-3p	NDC1
hsa_circ_0003528	hsa_miR_330-3p	MYPN
hsa_circ_0003528	hsa_miR_330-3p	SHOX2
hsa_circ_0003528	hsa_miR_330-3p	CDK1
hsa_circ_0003528	hsa_miR_330-3p	KCNC2
hsa_circ_0003528	hsa_miR_330-3p	S1PR1
hsa_circ_0003528	hsa_miR_330-3p	ONECUT2
hsa_circ_0003528	hsa_miR_330-3p	ELAVL2
hsa_circ_0003528	hsa_miR_330-3p	TBX5
hsa_circ_0003528	hsa_miR_330-3p	DTL
hsa_circ_0003528	hsa_miR_330-3p	SCG3
hsa_circ_0003528	hsa_miR_330-3p	SALL4
hsa_circ_0003528	hsa_miR_330-3p	SPRYD7
hsa_circ_0003528	hsa_miR_330-3p	FRAS1
hsa_circ_0003528	hsa_miR_330-3p	PGAM5
hsa_circ_0003528	hsa_miR_330-3p	TFAP2B
hsa_circ_0003528	hsa_miR_330-3p	SMAD7
hsa_circ_0003528	hsa_miR_330-3p	MGAM
hsa_circ_0003528	hsa_miR_330-3p	TPBG
hsa_circ_0003528	hsa_miR_330-3p	ARHGEF10
hsa_circ_0003528	hsa_miR_330-3p	JAM2
hsa_circ_0003528	hsa_miR_330-3p	SLC24A2
hsa_circ_0003528	hsa_miR_330-3p	MYRF
hsa_circ_0003528	hsa_miR_330-3p	ERBB4
hsa_circ_0003528	hsa_miR_330-3p	LAPTM5
hsa_circ_0003528	hsa_miR_330-3p	MICU3
hsa_circ_0003528	hsa_miR_330-3p	GBX2
hsa_circ_0003528	hsa_miR_330-3p	GDF6
hsa_circ_0003528	hsa_miR_330-3p	HOXC8
hsa_circ_0003528	hsa_miR_330-3p	SLC28A3
hsa_circ_0003528	hsa_miR_330-3p	GALNT7
hsa_circ_0003528	hsa_miR_330-3p	PROX1
hsa_circ_0003528	hsa_miR_330-3p	TSHR
hsa_circ_0003528	hsa_miR_330-3p	PPARGC1B
hsa_circ_0003528	hsa_miR_330-3p	COL6A5
hsa_circ_0003528	hsa_miR_330-3p	PTPRM
hsa_circ_0003528	hsa_miR_330-3p	RFWD3
hsa_circ_0003528	hsa_miR_330-3p	BFSP2
hsa_circ_0003528	hsa_miR_330-3p	DIRAS2

hsa_circ_0003528	hsa_miR_330-3p	KAT2B
hsa_circ_0003528	hsa_miR_330-3p	EGR4
hsa_circ_0003528	hsa_miR_330-3p	DRP2
hsa_circ_0003528	hsa_miR_330-3p	STXBP5L
hsa_circ_0003528	hsa_miR_330-3p	ST8SIA3
hsa_circ_0003528	hsa_miR_330-3p	KANK2
hsa_circ_0003528	hsa_miR_330-3p	NRG3
hsa_circ_0003528	hsa_miR_330-3p	LRP8
hsa_circ_0003528	hsa_miR_330-3p	ADCY9
hsa_circ_0003528	hsa_miR_330-3p	L1CAM
hsa_circ_0003528	hsa_miR_330-3p	C1orf21
hsa_circ_0003528	hsa_miR_330-3p	SEMA3A
hsa_circ_0003528	hsa_miR_330-3p	TGFBR3
hsa_circ_0003528	hsa_miR_330-3p	E2F1
hsa_circ_0003528	hsa_miR_330-3p	TNFAIP3
hsa_circ_0003528	hsa_miR_330-3p	NDNF
hsa_circ_0003528	hsa_miR_330-3p	SCN1A
hsa_circ_0003528	hsa_miR_330-3p	PCYT1B
hsa_circ_0003528	hsa_miR_330-3p	NAT8L
hsa_circ_0003528	hsa_miR_330-3p	PRKG1
hsa_circ_0003528	hsa_miR_330-3p	BMPR2
hsa_circ_0003528	hsa_miR_330-3p	NKAIN1
hsa_circ_0003528	hsa_miR_330-3p	ADCYAP1R1
hsa_circ_0003528	hsa_miR_330-3p	SYT5
hsa_circ_0003528	hsa_miR_330-3p	GJA3
hsa_circ_0003528	hsa_miR_330-3p	ADRA2A
hsa_circ_0003528	hsa_miR_330-3p	ATP2B2
hsa_circ_0003528	hsa_miR_330-3p	MYH10
hsa_circ_0003528	hsa_miR_330-3p	NPR3
hsa_circ_0003528	hsa_miR_330-3p	ANKRD33B
hsa_circ_0003528	hsa_miR_330-3p	FBLN5
hsa_circ_0003528	hsa_miR_330-3p	MAPK10
hsa_circ_0003528	hsa_miR_330-3p	JPH3
hsa_circ_0003528	hsa_miR_330-3p	CA12
hsa_circ_0003528	hsa_miR_330-3p	GPM6A
hsa_circ_0003528	hsa_miR_330-3p	PPM1H
hsa_circ_0003528	hsa_miR_330-3p	TYRP1
hsa_circ_0003528	hsa_miR_330-3p	PCDHA7
hsa_circ_0003528	hsa_miR_330-3p	PCDHA12
hsa_circ_0003528	hsa_miR_330-3p	PCDHA5
hsa_circ_0003528	hsa_miR_330-3p	PCDHA11
hsa_circ_0003528	hsa_miR_330-3p	PCDHA9
hsa_circ_0003528	hsa_miR_330-3p	PCDHA6
hsa_circ_0003528	hsa_miR_330-3p	PCDHA1
hsa_circ_0003528	hsa_miR_330-3p	PCDHA4
hsa_circ_0003528	hsa_miR_330-3p	ZBTB20
hsa_circ_0008274	hsa_miR_498	KLF6
hsa_circ_0008274	hsa_miR_498	ODC1
hsa_circ_0008274	hsa_miR_498	STK39
hsa_circ_0008274	hsa_miR_498	GCOM1
hsa_circ_0008274	hsa_miR_498	RSPH4A
hsa_circ_0008274	hsa_miR_498	DCLK1
hsa_circ_0008274	hsa_miR_498	DBF4
hsa_circ_0008274	hsa_miR_498	DCBLD2
hsa_circ_0008274	hsa_miR_498	POLQ
hsa_circ_0008274	hsa_miR_498	TRPC3
hsa_circ_0008274	hsa_miR_498	RANBP3L
hsa_circ_0008274	hsa_miR_498	SEMA6D

hsa_circ_0008274	hsa_miR_498	TRIM71
hsa_circ_0008274	hsa_miR_498	KCNT2
hsa_circ_0008274	hsa_miR_498	OLIG3
hsa_circ_0008274	hsa_miR_498	GJA1
hsa_circ_0008274	hsa_miR_498	SIX4
hsa_circ_0008274	hsa_miR_498	FERMT2
hsa_circ_0008274	hsa_miR_498	DAB2
hsa_circ_0008274	hsa_miR_498	SKP2
hsa_circ_0008274	hsa_miR_498	ZEB2
hsa_circ_0008274	hsa_miR_498	SLC2A3
hsa_circ_0008274	hsa_miR_498	TET1
hsa_circ_0008274	hsa_miR_498	C5orf64
hsa_circ_0008274	hsa_miR_498	CILP
hsa_circ_0008274	hsa_miR_498	DUSP4
hsa_circ_0008274	hsa_miR_498	FLVCR1
hsa_circ_0008274	hsa_miR_498	LIN28B
hsa_circ_0008274	hsa_miR_498	DSN1
hsa_circ_0008274	hsa_miR_498	GABRG2
hsa_circ_0008274	hsa_miR_498	SYNPO2
hsa_circ_0008274	hsa_miR_498	GALNT7
hsa_circ_0008274	hsa_miR_498	FA2H
hsa_circ_0008274	hsa_miR_498	DDX4
hsa_circ_0008274	hsa_miR_498	FRMD3
hsa_circ_0008274	hsa_miR_498	NOSTRIN
hsa_circ_0008274	hsa_miR_498	LRRC8C
hsa_circ_0008274	hsa_miR_498	PLD5
hsa_circ_0008274	hsa_miR_498	MYPN
hsa_circ_0008274	hsa_miR_498	KCNN3
hsa_circ_0008274	hsa_miR_498	ESRP1
hsa_circ_0008274	hsa_miR_498	SNRK
hsa_circ_0008274	hsa_miR_498	TLL1
hsa_circ_0008274	hsa_miR_498	MATN3
hsa_circ_0008274	hsa_miR_498	INSC
hsa_circ_0008274	hsa_miR_498	ONECUT2
hsa_circ_0008274	hsa_miR_498	STXBP5L
hsa_circ_0008274	hsa_miR_498	GABRA4
hsa_circ_0008274	hsa_miR_498	ELAVL4
hsa_circ_0008274	hsa_miR_498	TMTC1
hsa_circ_0008274	hsa_miR_498	CHODL
hsa_circ_0008274	hsa_miR_498	C2CD4A
hsa_circ_0008274	hsa_miR_498	VGLL3
hsa_circ_0008274	hsa_miR_498	PIK3R1
hsa_circ_0008274	hsa_miR_498	CACNA1A
hsa_circ_0008274	hsa_miR_498	MYH10
hsa_circ_0008274	hsa_miR_498	PCK1
hsa_circ_0008274	hsa_miR_498	INHBB
hsa_circ_0008274	hsa_miR_498	ZNF280B
hsa_circ_0008274	hsa_miR_498	LYPD6
hsa_circ_0008274	hsa_miR_498	TRIM2
hsa_circ_0008274	hsa_miR_498	RASIP1
hsa_circ_0008274	hsa_miR_498	MCF2
hsa_circ_0008274	hsa_miR_498	TRIM59
hsa_circ_0008274	hsa_miR_498	ZNF714
hsa_circ_0008274	hsa_miR_498	SMAD9
hsa_circ_0008274	hsa_miR_498	EYA4
hsa_circ_0008274	hsa_miR_498	SCIN
hsa_circ_0008274	hsa_miR_498	DCDC2
hsa_circ_0008274	hsa_miR_498	WDR72

hsa_circ_0008274	hsa_miR_498	CPNE4
hsa_circ_0008274	hsa_miR_498	HMGA2
hsa_circ_0008274	hsa_miR_498	TTPA
hsa_circ_0008274	hsa_miR_498	EGLN3
hsa_circ_0008274	hsa_miR_498	COL1A1
hsa_circ_0008274	hsa_miR_498	SCN2A
hsa_circ_0008274	hsa_miR_498	DLX5
hsa_circ_0008274	hsa_miR_498	RPL22L1
hsa_circ_0008274	hsa_miR_498	CA6
hsa_circ_0008274	hsa_miR_498	AK5
hsa_circ_0008274	hsa_miR_498	OLR1
hsa_circ_0008274	hsa_miR_498	GRIN2A
hsa_circ_0008274	hsa_miR_498	SLC7A11
hsa_circ_0008274	hsa_miR_498	TEX35
hsa_circ_0008274	hsa_miR_498	SEMA3A
hsa_circ_0008274	hsa_miR_498	IYD
hsa_circ_0008274	hsa_miR_498	CCNE1
hsa_circ_0008274	hsa_miR_498	KRTAP2-3
hsa_circ_0008274	hsa_miR_498	LINGO1
hsa_circ_0008274	hsa_miR_498	GPR26
hsa_circ_0008274	hsa_miR_498	TFAP2C
hsa_circ_0008274	hsa_miR_498	COL11A1
hsa_circ_0008274	hsa_miR_498	S100A16
hsa_circ_0008274	hsa_miR_498	TNNT1
hsa_circ_0008274	hsa_miR_498	LGSN
hsa_circ_0008274	hsa_miR_498	C1orf56
hsa_circ_0008274	hsa_miR_498	LIFR
hsa_circ_0008274	hsa_miR_498	ZFHX4
hsa_circ_0008274	hsa_miR_498	TIGD3
hsa_circ_0008274	hsa_miR_498	ATCAY
hsa_circ_0008274	hsa_miR_498	CBX4
hsa_circ_0008274	hsa_miR_498	ARL13B
hsa_circ_0008274	hsa_miR_498	GRIA2
hsa_circ_0008274	hsa_miR_498	SGK1
hsa_circ_0008274	hsa_miR_498	FGD6
hsa_circ_0008274	hsa_miR_498	KCNK10
hsa_circ_0008274	hsa_miR_498	ZNF681
hsa_circ_0008274	hsa_miR_498	TRDN
hsa_circ_0008274	hsa_miR_498	IL19
hsa_circ_0008274	hsa_miR_498	PRKCQ
hsa_circ_0008274	hsa_miR_498	BTK
hsa_circ_0008274	hsa_miR_498	CPLX2
hsa_circ_0008274	hsa_miR_498	MPP6
hsa_circ_0008274	hsa_miR_498	PHACTR2
hsa_circ_0008274	hsa_miR_498	LOXL2
hsa_circ_0008274	hsa_miR_498	ZNF385B
hsa_circ_0008274	hsa_miR_498	ALDH18A1
hsa_circ_0008274	hsa_miR_498	SFXN4
hsa_circ_0008274	hsa_miR_498	ATP1A2
hsa_circ_0008274	hsa_miR_498	EPCAM
hsa_circ_0008274	hsa_miR_498	CD93
hsa_circ_0008274	hsa_miR_498	SVOP
hsa_circ_0008274	hsa_miR_498	RERG
hsa_circ_0008274	hsa_miR_498	TTLL2
hsa_circ_0008274	hsa_miR_498	TMEM196
hsa_circ_0008274	hsa_miR_498	FAM135B
hsa_circ_0008274	hsa_miR_498	MEX3A
hsa_circ_0008274	hsa_miR_498	ST8SIA2

hsa_circ_0008274	hsa_miR_498	SOCS3
hsa_circ_0008274	hsa_miR_498	RBM24
hsa_circ_0008274	hsa_miR_498	ENC1
hsa_circ_0008274	hsa_miR_498	TNS1
hsa_circ_0008274	hsa_miR_498	NLN
hsa_circ_0008274	hsa_miR_498	GRHL3
hsa_circ_0008274	hsa_miR_498	SLC16A6
hsa_circ_0008274	hsa_miR_498	PDIA6
hsa_circ_0008274	hsa_miR_498	SYT14
hsa_circ_0008274	hsa_miR_498	KLF10
hsa_circ_0008274	hsa_miR_498	EMB
hsa_circ_0008274	hsa_miR_498	SLCO5A1
hsa_circ_0008274	hsa_miR_498	GATA1
hsa_circ_0008274	hsa_miR_498	SLCO1A2
hsa_circ_0008274	hsa_miR_498	ST8SIA3
hsa_circ_0008274	hsa_miR_498	MEX3B
hsa_circ_0008274	hsa_miR_498	ITGA2
hsa_circ_0008274	hsa_miR_498	TMPRSS11A
hsa_circ_0008274	hsa_miR_498	SLC26A4
hsa_circ_0008274	hsa_miR_498	SLC24A4
hsa_circ_0008274	hsa_miR_498	EPB41L3
hsa_circ_0008274	hsa_miR_498	GAL3ST3
hsa_circ_0008274	hsa_miR_498	COBL
hsa_circ_0008274	hsa_miR_498	FUT9
hsa_circ_0008274	hsa_miR_498	HOXA9
hsa_circ_0008274	hsa_miR_498	MYO1F
hsa_circ_0008274	hsa_miR_498	TBX5
hsa_circ_0008274	hsa_miR_498	DNAJB4
hsa_circ_0008274	hsa_miR_498	VTCN1
hsa_circ_0008274	hsa_miR_498	KCNC2
hsa_circ_0008274	hsa_miR_498	SLC9A2
hsa_circ_0008274	hsa_miR_498	ADAM12
hsa_circ_0008274	hsa_miR_498	SPAG6
hsa_circ_0008274	hsa_miR_498	PRH2
hsa_circ_0008274	hsa_miR_498	SPN
hsa_circ_0008274	hsa_miR_498	MNX1
hsa_circ_0008274	hsa_miR_498	FRMD4A
hsa_circ_0008274	hsa_miR_498	MSMO1
hsa_circ_0008274	hsa_miR_498	SGMS2
hsa_circ_0008274	hsa_miR_498	WASF1
hsa_circ_0008274	hsa_miR_498	OPCML
hsa_circ_0008274	hsa_miR_498	CHAC1
hsa_circ_0008274	hsa_miR_498	ADAM28
hsa_circ_0008274	hsa_miR_498	TFPI2
hsa_circ_0008274	hsa_miR_498	IQUB
hsa_circ_0008274	hsa_miR_498	NOVA1
hsa_circ_0008274	hsa_miR_498	SLC3A1
hsa_circ_0008274	hsa_miR_498	KLF9
hsa_circ_0008274	hsa_miR_498	C7
hsa_circ_0008274	hsa_miR_498	PGM2L1
hsa_circ_0008274	hsa_miR_498	GDF10
hsa_circ_0008274	hsa_miR_656	WIF1
hsa_circ_0008274	hsa_miR_656	PKHD1
hsa_circ_0008274	hsa_miR_656	KLF4
hsa_circ_0008274	hsa_miR_656	ARHGAP6
hsa_circ_0008274	hsa_miR_656	PRKG1
hsa_circ_0008274	hsa_miR_656	PI15
hsa_circ_0008274	hsa_miR_656	KL

hsa_circ_0008274	hsa_miR_656	MPP6
hsa_circ_0008274	hsa_miR_656	ARL13B
hsa_circ_0008274	hsa_miR_656	STEAP2
hsa_circ_0008274	hsa_miR_656	EIF4E3
hsa_circ_0008274	hsa_miR_656	CADM4
hsa_circ_0008274	hsa_miR_656	RORA
hsa_circ_0008274	hsa_miR_656	ARHGEF6
hsa_circ_0008274	hsa_miR_656	KCNH5
hsa_circ_0008274	hsa_miR_656	LRRC31
hsa_circ_0008274	hsa_miR_656	TENM1
hsa_circ_0008274	hsa_miR_656	RGCC
hsa_circ_0008274	hsa_miR_656	EREG
hsa_circ_0008274	hsa_miR_656	ZFP36
hsa_circ_0008274	hsa_miR_656	NCAPG2
hsa_circ_0008274	hsa_miR_656	GRHL1
hsa_circ_0008274	hsa_miR_656	NR4A1
hsa_circ_0008274	hsa_miR_656	TTYH3
hsa_circ_0008274	hsa_miR_656	HS3ST3A1
hsa_circ_0008274	hsa_miR_656	INSC
hsa_circ_0008274	hsa_miR_656	LSAMP
hsa_circ_0008274	hsa_miR_656	GFPT1
hsa_circ_0008274	hsa_miR_656	JUNB
hsa_circ_0008274	hsa_miR_656	EGR3
hsa_circ_0008274	hsa_miR_656	DDX4
hsa_circ_0008274	hsa_miR_656	NEIL3
hsa_circ_0008274	hsa_miR_656	HHIP
hsa_circ_0008274	hsa_miR_656	ITGB8
hsa_circ_0008274	hsa_miR_656	TRIP13
hsa_circ_0008274	hsa_miR_656	SPIRE2
hsa_circ_0008274	hsa_miR_656	LMOD1
hsa_circ_0008274	hsa_miR_656	TBX18
hsa_circ_0008274	hsa_miR_656	TAS2R20
hsa_circ_0008274	hsa_miR_656	BDNF
hsa_circ_0008274	hsa_miR_656	ZNF714
hsa_circ_0008274	hsa_miR_656	TRPA1
hsa_circ_0008274	hsa_miR_656	FRY
hsa_circ_0008274	hsa_miR_656	ANKRD18A
hsa_circ_0008274	hsa_miR_656	EYA4
hsa_circ_0008274	hsa_miR_656	PLD5
hsa_circ_0008274	hsa_miR_656	CALCRL
hsa_circ_0008274	hsa_miR_656	PCDH7
hsa_circ_0008274	hsa_miR_656	DUSP1
hsa_circ_0008274	hsa_miR_656	TMEM178B
hsa_circ_0008274	hsa_miR_656	ARMC3
hsa_circ_0008274	hsa_miR_656	GUCY1A2
hsa_circ_0008274	hsa_miR_656	CYP8B1
hsa_circ_0008274	hsa_miR_656	NCKAP5
hsa_circ_0008274	hsa_miR_656	CAV2
hsa_circ_0008274	hsa_miR_656	TIMP3
hsa_circ_0008274	hsa_miR_656	TNFAIP3
hsa_circ_0008274	hsa_miR_656	CYP26B1
hsa_circ_0008274	hsa_miR_656	KRT5
hsa_circ_0008274	hsa_miR_656	SCN8A
hsa_circ_0008274	hsa_miR_656	NEGR1
hsa_circ_0008274	hsa_miR_656	SRCIN1
hsa_circ_0008274	hsa_miR_656	SATB2
hsa_circ_0008274	hsa_miR_656	VAMP2
hsa_circ_0008274	hsa_miR_656	TCP10L2

hsa_circ_0008274	hsa_miR_656	EDNRB
hsa_circ_0008274	hsa_miR_656	FUT9
hsa_circ_0008274	hsa_miR_656	SASS6
hsa_circ_0008274	hsa_miR_656	C8orf34
hsa_circ_0008274	hsa_miR_656	CCND2
hsa_circ_0008274	hsa_miR_656	ELAVL4
hsa_circ_0008274	hsa_miR_656	KAT2B
hsa_circ_0008274	hsa_miR_656	TMTC1
hsa_circ_0008274	hsa_miR_656	STK32A
hsa_circ_0008274	hsa_miR_656	PTPRM
hsa_circ_0008274	hsa_miR_656	KLF2
hsa_circ_0008274	hsa_miR_656	KCNN3
hsa_circ_0008274	hsa_miR_656	LRP1B
hsa_circ_0008274	hsa_miR_656	TNS1
hsa_circ_0008274	hsa_miR_656	ZEB1
hsa_circ_0008274	hsa_miR_656	CEACAM1
hsa_circ_0008274	hsa_miR_656	ST6GALNAC3
hsa_circ_0008274	hsa_miR_656	MSR1
hsa_circ_0008274	hsa_miR_656	NPR3
hsa_circ_0008274	hsa_miR_656	KYNU
hsa_circ_0008274	hsa_miR_656	SLC16A7
hsa_circ_0008274	hsa_miR_656	TNFRSF11A
hsa_circ_0008274	hsa_miR_656	RGR
hsa_circ_0008274	hsa_miR_656	ZNF681
hsa_circ_0008274	hsa_miR_656	MUC17
hsa_circ_0008274	hsa_miR_656	TBX5
hsa_circ_0008274	hsa_miR_656	ARHGAP44
hsa_circ_0008274	hsa_miR_656	PMP22
hsa_circ_0008274	hsa_miR_656	GABRA5
hsa_circ_0008274	hsa_miR_656	ONECUT2
hsa_circ_0008274	hsa_miR_656	SLIT2
hsa_circ_0008274	hsa_miR_656	IL20RA
hsa_circ_0008274	hsa_miR_656	HNF4G
hsa_circ_0008274	hsa_miR_656	MKRN3
hsa_circ_0008274	hsa_miR_656	OIP5
hsa_circ_0008274	hsa_miR_656	UGT2B28
hsa_circ_0008274	hsa_miR_656	ZNF117
hsa_circ_0008274	hsa_miR_656	PRKAA2
hsa_circ_0008274	hsa_miR_656	MATN3
hsa_circ_0008274	hsa_miR_656	STARD13
hsa_circ_0008274	hsa_miR_656	ZNF485
hsa_circ_0008274	hsa_miR_656	CCDC141
hsa_circ_0008274	hsa_miR_656	COLGALT2
hsa_circ_0008274	hsa_miR_656	PCSK1
hsa_circ_0008274	hsa_miR_656	DCAF13
hsa_circ_0008274	hsa_miR_656	FAM136A
hsa_circ_0008274	hsa_miR_656	DARS2
hsa_circ_0008274	hsa_miR_656	CTLA4
hsa_circ_0008274	hsa_miR_656	PBLD
hsa_circ_0008274	hsa_miR_656	TLR4
hsa_circ_0008274	hsa_miR_656	CNR1
hsa_circ_0008274	hsa_miR_656	TGFBR3
hsa_circ_0008274	hsa_miR_656	EMILIN2
hsa_circ_0008274	hsa_miR_656	SPHKAP
hsa_circ_0008274	hsa_miR_656	KLF6
hsa_circ_0008274	hsa_miR_656	CENPK
hsa_circ_0008274	hsa_miR_656	SPATA18
hsa_circ_0008274	hsa_miR_656	RASSF6

hsa_circ_0008274	hsa_miR_656	UGT2B15
hsa_circ_0008274	hsa_miR_656	TFAP2D
hsa_circ_0008274	hsa_miR_656	PAQR9
hsa_circ_0008274	hsa_miR_656	TNC
hsa_circ_0008274	hsa_miR_656	FRMD4B
hsa_circ_0008274	hsa_miR_656	HECW2
hsa_circ_0008274	hsa_miR_656	PLSCR4
hsa_circ_0008274	hsa_miR_656	LRRC15
hsa_circ_0008274	hsa_miR_656	GABRR2
hsa_circ_0008274	hsa_miR_656	SUV39H2
hsa_circ_0008274	hsa_miR_656	ZEB2
hsa_circ_0008274	hsa_miR_656	GRIK2
hsa_circ_0008274	hsa_miR_656	ANGPTL1
hsa_circ_0008274	hsa_miR_656	CAB39L
hsa_circ_0008274	hsa_miR_656	CHML
hsa_circ_0008274	hsa_miR_656	ITGA2
hsa_circ_0008274	hsa_miR_656	VNN1
hsa_circ_0008274	hsa_miR_656	CCL28
hsa_circ_0008274	hsa_miR_656	PRDM12
hsa_circ_0008274	hsa_miR_656	SNTG1
hsa_circ_0008274	hsa_miR_656	C12orf40
hsa_circ_0008274	hsa_miR_656	CTH
hsa_circ_0008274	hsa_miR_656	FAM177B
hsa_circ_0008274	hsa_miR_656	WWC2
hsa_circ_0008274	hsa_miR_656	GDF10
hsa_circ_0008274	hsa_miR_656	ANGPTL5
hsa_circ_0008274	hsa_miR_656	ANGPT1
hsa_circ_0008274	hsa_miR_656	ZBTB20
hsa_circ_0008274	hsa_miR_656	TMEFF1
hsa_circ_0008274	hsa_miR_656	NOVA1
hsa_circ_0008274	hsa_miR_656	PLCXD3
hsa_circ_0008274	hsa_miR_656	SIX4
hsa_circ_0008274	hsa_miR_656	NFATC1
hsa_circ_0008274	hsa_miR_656	CHRNA5
hsa_circ_0008274	hsa_miR_656	PRLR
hsa_circ_0008274	hsa_miR_656	CRHR1
hsa_circ_0008274	hsa_miR_656	PHACTR2
hsa_circ_0008274	hsa_miR_656	COL11A1
hsa_circ_0008274	hsa_miR_656	GRIN2A
hsa_circ_0008274	hsa_miR_656	ARHGAP29
hsa_circ_0008274	hsa_miR_656	SMAD6
hsa_circ_0008274	hsa_miR_656	SERPINA7
hsa_circ_0008274	hsa_miR_656	RIMS2
hsa_circ_0008274	hsa_miR_656	SLC9A2
hsa_circ_0008274	hsa_miR_656	CYP2B6
hsa_circ_0008274	hsa_miR_656	RALGPS2
hsa_circ_0008274	hsa_miR_656	KLRD1
hsa_circ_0008274	hsa_miR_656	FGF5
hsa_circ_0008274	hsa_miR_656	ETV1
hsa_circ_0008274	hsa_miR_656	SPRYD7
hsa_circ_0008274	hsa_miR_656	SRD5A3
hsa_circ_0008274	hsa_miR_656	CALCR
hsa_circ_0008274	hsa_miR_656	EXD1
hsa_circ_0008274	hsa_miR_656	MAPK10
hsa_circ_0008274	hsa_miR_656	CCSER1
hsa_circ_0008274	hsa_miR_656	MSI2
hsa_circ_0008274	hsa_miR_656	DGKI
hsa_circ_0008274	hsa_miR_656	VWC2

hsa_circ_0008274	hsa_miR_656	WNT7A
hsa_circ_0008274	hsa_miR_656	UGT2B10
hsa_circ_0008274	hsa_miR_656	TRPC3
hsa_circ_0008274	hsa_miR_656	AFF3
hsa_circ_0008274	hsa_miR_656	NHS
hsa_circ_0008274	hsa_miR_656	CCDC170
hsa_circ_0008274	hsa_miR_656	TNFSF4
hsa_circ_0008274	hsa_miR_656	C1QTNF2
hsa_circ_0008274	hsa_miR_656	LMO7
hsa_circ_0008274	hsa_miR_656	GCSAML
hsa_circ_0008274	hsa_miR_656	PGM2L1
hsa_circ_0008274	hsa_miR_656	NUP62CL
hsa_circ_0008274	hsa_miR_656	LGSN
hsa_circ_0008274	hsa_miR_656	PNLIPRP3
hsa_circ_0008274	hsa_miR_656	SLC6A15
hsa_circ_0008274	hsa_miR_656	DMRT3
hsa_circ_0008274	hsa_miR_656	SCN3A
hsa_circ_0008274	hsa_miR_656	GRIA2
hsa_circ_0008274	hsa_miR_656	CLVS2
hsa_circ_0008274	hsa_miR_656	FRMD3
hsa_circ_0008274	hsa_miR_656	TCF4
hsa_circ_0008274	hsa_miR_656	SASH1
hsa_circ_0008274	hsa_miR_656	DCX
hsa_circ_0008274	hsa_miR_656	PRDM5
hsa_circ_0008274	hsa_miR_656	SLC7A11
hsa_circ_0008274	hsa_miR_656	PREX2
hsa_circ_0008274	hsa_miR_656	TEK
hsa_circ_0008274	hsa_miR_656	MMP8
hsa_circ_0008274	hsa_miR_656	OVOL1
hsa_circ_0008274	hsa_miR_656	TACC1
hsa_circ_0008274	hsa_miR_656	GAS7
hsa_circ_0008274	hsa_miR_656	KIAA1549L
hsa_circ_0008274	hsa_miR_656	ZNF107
hsa_circ_0008274	hsa_miR_656	ZIC4
hsa_circ_0008274	hsa_miR_656	SHANK3
hsa_circ_0008274	hsa_miR_656	CRIM1
hsa_circ_0008274	hsa_miR_656	CD163
hsa_circ_0008274	hsa_miR_656	TET1
hsa_circ_0008274	hsa_miR_656	IL21
hsa_circ_0008274	hsa_miR_656	KCNAB1
hsa_circ_0008274	hsa_miR_656	CACNB4
hsa_circ_0008274	hsa_miR_656	FSHB
hsa_circ_0008274	hsa_miR_656	MEI4
hsa_circ_0008274	hsa_miR_656	PRR11
hsa_circ_0008274	hsa_miR_656	ANGPTL3
hsa_circ_0008274	hsa_miR_656	CD36
hsa_circ_0008274	hsa_miR_656	TMEM229A
hsa_circ_0008274	hsa_miR_656	MMACHC
hsa_circ_0008274	hsa_miR_656	HCN1
hsa_circ_0008274	hsa_miR_656	GCLM
hsa_circ_0008274	hsa_miR_656	FSTL5
hsa_circ_0008274	hsa_miR_656	TPD52
hsa_circ_0008274	hsa_miR_656	HOXC4
hsa_circ_0008274	hsa_miR_656	PIRT
hsa_circ_0008274	hsa_miR_656	SLC16A14
hsa_circ_0008274	hsa_miR_656	VCAN
hsa_circ_0008274	hsa_miR_656	C1QTNF3
hsa_circ_0008274	hsa_miR_656	FGD6

hsa_circ_0008274	hsa_miR_656	OLIG2
hsa_circ_0008274	hsa_miR_656	TMEM47
hsa_circ_0008274	hsa_miR_656	SLCO1B3
hsa_circ_0008274	hsa_miR_656	SEC11C
hsa_circ_0008274	hsa_miR_656	KCNJ10
hsa_circ_0008274	hsa_miR_656	CERS6
hsa_circ_0008274	hsa_miR_656	CDC14A
hsa_circ_0008274	hsa_miR_656	LMNB2
hsa_circ_0008274	hsa_miR_656	GABRA4
hsa_circ_0008274	hsa_miR_656	LCOR
hsa_circ_0008274	hsa_miR_656	SLC16A1
hsa_circ_0008274	hsa_miR_656	DPYSL2
hsa_circ_0008274	hsa_miR_656	TRDN
hsa_circ_0008274	hsa_miR_656	PEX5L
hsa_circ_0008274	hsa_miR_656	CNKSR2
hsa_circ_0008274	hsa_miR_656	COL25A1
hsa_circ_0008274	hsa_miR_656	SH3BP5
hsa_circ_0008274	hsa_miR_656	PLEKHH2
hsa_circ_0008274	hsa_miR_656	HBEGF
hsa_circ_0008274	hsa_miR_656	SLC41A2
hsa_circ_0008274	hsa_miR_656	SOX21
hsa_circ_0008274	hsa_miR_656	AKAP7
hsa_circ_0008274	hsa_miR_656	SCG3
hsa_circ_0008274	hsa_miR_656	TTPA
hsa_circ_0008274	hsa_miR_656	GDA
hsa_circ_0008274	hsa_miR_656	GRTP1
hsa_circ_0008274	hsa_miR_656	BCO2
hsa_circ_0008274	hsa_miR_656	TLL2
hsa_circ_0008274	hsa_miR_656	IFNG
hsa_circ_0008274	hsa_miR_656	MDK
hsa_circ_0008274	hsa_miR_656	E2F3
hsa_circ_0008274	hsa_miR_656	SYT1
hsa_circ_0008274	hsa_miR_656	SFRP2
hsa_circ_0008274	hsa_miR_656	TMEM74
hsa_circ_0008274	hsa_miR_656	MAP10
hsa_circ_0008274	hsa_miR_656	CYBB
hsa_circ_0008274	hsa_miR_656	TTC29
hsa_circ_0008274	hsa_miR_656	GREB1
hsa_circ_0008274	hsa_miR_656	TCF24
hsa_circ_0008274	hsa_miR_656	MYT1
hsa_circ_0008274	hsa_miR_656	ZNF676
hsa_circ_0008274	hsa_miR_656	DOCK4
hsa_circ_0008274	hsa_miR_656	CCNA1
hsa_circ_0008274	hsa_miR_140-3p	PCDH17
hsa_circ_0008274	hsa_miR_140-3p	GPR158
hsa_circ_0008274	hsa_miR_140-3p	TTYH3
hsa_circ_0008274	hsa_miR_140-3p	NDST1
hsa_circ_0008274	hsa_miR_140-3p	SOBP
hsa_circ_0008274	hsa_miR_140-3p	PCDH8
hsa_circ_0008274	hsa_miR_140-3p	DNM1
hsa_circ_0008274	hsa_miR_140-3p	PEAK1
hsa_circ_0008274	hsa_miR_140-3p	CHL1
hsa_circ_0008274	hsa_miR_140-3p	MVB12B
hsa_circ_0008274	hsa_miR_140-3p	ADCY9
hsa_circ_0008274	hsa_miR_140-3p	RASGEF1B
hsa_circ_0008274	hsa_miR_140-3p	HPCAL1
hsa_circ_0008274	hsa_miR_140-3p	NDC1
hsa_circ_0008274	hsa_miR_140-3p	NOVA1

hsa_circ_0008274	hsa_miR_140-3p	ROBO2
hsa_circ_0008274	hsa_miR_140-3p	SLC5A3
hsa_circ_0008274	hsa_miR_140-3p	OTX2
hsa_circ_0008274	hsa_miR_140-3p	E2F7
hsa_circ_0008274	hsa_miR_140-3p	B3GALNT1
hsa_circ_0008274	hsa_miR_140-3p	G6PC2
hsa_circ_0008274	hsa_miR_140-3p	TCF4
hsa_circ_0008274	hsa_miR_140-3p	SYNC
hsa_circ_0008274	hsa_miR_140-3p	ELAVL2
hsa_circ_0008274	hsa_miR_140-3p	PAX7
hsa_circ_0008274	hsa_miR_140-3p	SIRPA
hsa_circ_0008274	hsa_miR_140-3p	KCNB1
hsa_circ_0008274	hsa_miR_140-3p	BCL9
hsa_circ_0008274	hsa_miR_140-3p	HOXA9
hsa_circ_0008274	hsa_miR_140-3p	PAPSS2
hsa_circ_0008274	hsa_miR_140-3p	VGLL2
hsa_circ_0008274	hsa_miR_140-3p	PRKG1
hsa_circ_0008274	hsa_miR_140-3p	EGLN3
hsa_circ_0008274	hsa_miR_140-3p	RASSF6
hsa_circ_0008274	hsa_miR_140-3p	MAP2K6
hsa_circ_0008274	hsa_miR_140-3p	HS3ST5
hsa_circ_0008274	hsa_miR_140-3p	TACC1
hsa_circ_0008274	hsa_miR_140-3p	MMP16
hsa_circ_0008274	hsa_miR_140-3p	ONECUT2
hsa_circ_0008274	hsa_miR_140-3p	NKAIN2
hsa_circ_0008274	hsa_miR_140-3p	AK4
hsa_circ_0008274	hsa_miR_140-3p	KHDRBS2
hsa_circ_0008274	hsa_miR_140-3p	LATS2
hsa_circ_0008274	hsa_miR_140-3p	KIF5A
hsa_circ_0008274	hsa_miR_140-3p	MOB3B
hsa_circ_0008274	hsa_miR_140-3p	FXYD6
hsa_circ_0008274	hsa_miR_140-3p	CABLES2
hsa_circ_0008274	hsa_miR_140-3p	KLF4
hsa_circ_0029426	hsa_miR_942	LRP8
hsa_circ_0029426	hsa_miR_942	KNG1
hsa_circ_0029426	hsa_miR_942	RANBP3L
hsa_circ_0029426	hsa_miR_942	THRB
hsa_circ_0029426	hsa_miR_942	HSPD1
hsa_circ_0029426	hsa_miR_942	CCBE1
hsa_circ_0029426	hsa_miR_942	WASF3
hsa_circ_0029426	hsa_miR_942	C1orf56
hsa_circ_0029426	hsa_miR_942	CHEK1
hsa_circ_0029426	hsa_miR_942	CACNB4
hsa_circ_0029426	hsa_miR_942	CLVS2
hsa_circ_0029426	hsa_miR_942	VGLL3
hsa_circ_0029426	hsa_miR_942	TMEM178B
hsa_circ_0029426	hsa_miR_942	RAD54B
hsa_circ_0029426	hsa_miR_942	FUT8
hsa_circ_0029426	hsa_miR_942	B3GALNT1
hsa_circ_0029426	hsa_miR_942	DEPDC4
hsa_circ_0029426	hsa_miR_942	SMKR1
hsa_circ_0029426	hsa_miR_942	TAT
hsa_circ_0029426	hsa_miR_942	KIAA1549L
hsa_circ_0029426	hsa_miR_942	P2RY12
hsa_circ_0029426	hsa_miR_942	LSAMP
hsa_circ_0029426	hsa_miR_942	CCL14
hsa_circ_0029426	hsa_miR_942	TNS1
hsa_circ_0029426	hsa_miR_942	PACRG

hsa_circ_0029426	hsa_miR_942	PPM1F
hsa_circ_0029426	hsa_miR_942	PIF1
hsa_circ_0029426	hsa_miR_942	TGFA
hsa_circ_0029426	hsa_miR_942	CACNA1E
hsa_circ_0029426	hsa_miR_942	GDNF
hsa_circ_0029426	hsa_miR_942	DDX4
hsa_circ_0029426	hsa_miR_942	CD83
hsa_circ_0029426	hsa_miR_942	GINS4
hsa_circ_0029426	hsa_miR_942	C8A
hsa_circ_0029426	hsa_miR_942	PRKG1
hsa_circ_0029426	hsa_miR_942	NCKAP5
hsa_circ_0029426	hsa_miR_942	ANKRD34B
hsa_circ_0029426	hsa_miR_942	EMP2
hsa_circ_0029426	hsa_miR_942	DCAF4L2
hsa_circ_0029426	hsa_miR_942	AVL9
hsa_circ_0029426	hsa_miR_942	RAB3B
hsa_circ_0029426	hsa_miR_942	NECAB1
hsa_circ_0029426	hsa_miR_942	KCNB1
hsa_circ_0029426	hsa_miR_942	HPRT1
hsa_circ_0029426	hsa_miR_942	PKP2
hsa_circ_0029426	hsa_miR_942	HOXA10
hsa_circ_0029426	hsa_miR_942	WDHD1
hsa_circ_0029426	hsa_miR_942	MEX3A
hsa_circ_0029426	hsa_miR_942	SLC17A2
hsa_circ_0029426	hsa_miR_942	SGPL1
hsa_circ_0029426	hsa_miR_942	LRRC15
hsa_circ_0029426	hsa_miR_942	BEST4
hsa_circ_0029426	hsa_miR_942	OPCML
hsa_circ_0029426	hsa_miR_942	OTC
hsa_circ_0029426	hsa_miR_942	CSMD2
hsa_circ_0029426	hsa_miR_942	CKAP2
hsa_circ_0029426	hsa_miR_942	NEDD9
hsa_circ_0029426	hsa_miR_942	PLCL1
hsa_circ_0029426	hsa_miR_942	PDK1
hsa_circ_0029426	hsa_miR_942	EPHA7
hsa_circ_0029426	hsa_miR_942	GFPT1
hsa_circ_0029426	hsa_miR_942	COL6A6
hsa_circ_0029426	hsa_miR_942	PDK4
hsa_circ_0029426	hsa_miR_942	HMBS
hsa_circ_0029426	hsa_miR_942	NFASC
hsa_circ_0029426	hsa_miR_942	SPDYA
hsa_circ_0029426	hsa_miR_942	ROBO4
hsa_circ_0029426	hsa_miR_942	CLDN12
hsa_circ_0029426	hsa_miR_942	HS3ST5
hsa_circ_0029426	hsa_miR_942	FGFR2
hsa_circ_0029426	hsa_miR_942	PARPBP
hsa_circ_0029426	hsa_miR_942	ZNF710
hsa_circ_0029426	hsa_miR_942	LRRC10B
hsa_circ_0029426	hsa_miR_942	GUCY1A2
hsa_circ_0029426	hsa_miR_942	PCDH17
hsa_circ_0029426	hsa_miR_942	ONECUT2
hsa_circ_0029426	hsa_miR_942	HTR4
hsa_circ_0029426	hsa_miR_942	EDARADD
hsa_circ_0029426	hsa_miR_942	PHACTR2
hsa_circ_0029426	hsa_miR_942	STARD8
hsa_circ_0029426	hsa_miR_942	FCRL4
hsa_circ_0029426	hsa_miR_942	PDE1C
hsa_circ_0029426	hsa_miR_942	OR51E1

hsa_circ_0029426	hsa_miR_942	CACNG8
hsa_circ_0029426	hsa_miR_942	NFIX
hsa_circ_0029426	hsa_miR_942	NTM
hsa_circ_0029426	hsa_miR_942	BACE2
hsa_circ_0029426	hsa_miR_942	HS3ST3A1
hsa_circ_0029426	hsa_miR_942	RASSF6
hsa_circ_0029426	hsa_miR_942	ZEB2
hsa_circ_0029426	hsa_miR_942	ARRB1
hsa_circ_0029426	hsa_miR_942	CHRDL1
hsa_circ_0029426	hsa_miR_942	LIN28B
hsa_circ_0029426	hsa_miR_942	SEMA5A
hsa_circ_0029426	hsa_miR_942	FAXC
hsa_circ_0029426	hsa_miR_942	MANEAL
hsa_circ_0029426	hsa_miR_942	MSRB3
hsa_circ_0029426	hsa_miR_942	KCNC1
hsa_circ_0029426	hsa_miR_942	GATA6
hsa_circ_0029426	hsa_miR_942	TSPAN5
hsa_circ_0029426	hsa_miR_942	SDK1
hsa_circ_0029426	hsa_miR_942	F2RL3
hsa_circ_0029426	hsa_miR_942	ETS1
hsa_circ_0029426	hsa_miR_942	TEX13B
hsa_circ_0029426	hsa_miR_942	TEK
hsa_circ_0029426	hsa_miR_942	SLC1A1
hsa_circ_0029426	hsa_miR_942	SCAI
hsa_circ_0029426	hsa_miR_942	KDM5B
hsa_circ_0029426	hsa_miR_942	C1orf115
hsa_circ_0029426	hsa_miR_942	PLA2G4F
hsa_circ_0029426	hsa_miR_942	ZNF106
hsa_circ_0029426	hsa_miR_942	MEF2B
hsa_circ_0029426	hsa_miR_942	DPP4
hsa_circ_0029426	hsa_miR_942	TMPRSS11D
hsa_circ_0029426	hsa_miR_942	SLC6A11
hsa_circ_0029426	hsa_miR_942	USP2
hsa_circ_0029426	hsa_miR_942	GRID1
hsa_circ_0029426	hsa_miR_942	ENDOU
hsa_circ_0029426	hsa_miR_942	HECW1
hsa_circ_0029426	hsa_miR_942	TLL2
hsa_circ_0029426	hsa_miR_942	CBR1
hsa_circ_0029426	hsa_miR_942	EIF4E3
hsa_circ_0029426	hsa_miR_942	CCL25
hsa_circ_0029426	hsa_miR_942	KRT4
hsa_circ_0029426	hsa_miR_942	CNGB1
hsa_circ_0029426	hsa_miR_942	ZNF117
hsa_circ_0029426	hsa_miR_942	ASCL1
hsa_circ_0029426	hsa_miR_942	NR2E1
hsa_circ_0029426	hsa_miR_942	SLC24A2
hsa_circ_0029426	hsa_miR_942	STOML3
hsa_circ_0029426	hsa_miR_942	COL11A2
hsa_circ_0029426	hsa_miR_942	RNF157
hsa_circ_0029426	hsa_miR_942	SECISBP2L
hsa_circ_0029426	hsa_miR_942	RDH10
hsa_circ_0029426	hsa_miR_942	PAPPA
hsa_circ_0029426	hsa_miR_942	NMNAT2
hsa_circ_0029426	hsa_miR_942	CLEC6A
hsa_circ_0029426	hsa_miR_942	PPP2R2C
hsa_circ_0029426	hsa_miR_942	PUS7
hsa_circ_0029426	hsa_miR_942	GPR26
hsa_circ_0029426	hsa_miR_942	KANK4

hsa_circ_0029426	hsa_miR_942	HK3
hsa_circ_0029426	hsa_miR_942	GPR158
hsa_circ_0029426	hsa_miR_942	SLIT3
hsa_circ_0029426	hsa_miR_942	LPGAT1
hsa_circ_0029426	hsa_miR_942	SLC5A5
hsa_circ_0029426	hsa_miR_942	EGLN3
hsa_circ_0029426	hsa_miR_942	LRRC20
hsa_circ_0029426	hsa_miR_942	THBD
hsa_circ_0029426	hsa_miR_942	IL7R
hsa_circ_0029426	hsa_miR_942	MAPK10
hsa_circ_0029426	hsa_miR_942	SH3BP5
hsa_circ_0029426	hsa_miR_942	OTOGL
hsa_circ_0029426	hsa_miR_942	BOLA1
hsa_circ_0029426	hsa_miR_942	PAX7
hsa_circ_0029426	hsa_miR_942	NPR3
hsa_circ_0029426	hsa_miR_942	RNF43
hsa_circ_0029426	hsa_miR_942	TOM1L2
hsa_circ_0029426	hsa_miR_942	ZNF217
hsa_circ_0029426	hsa_miR_942	RNFT2
hsa_circ_0029426	hsa_miR_942	CBS
hsa_circ_0029426	hsa_miR_942	CPLX1
hsa_circ_0029426	hsa_miR_942	PRLR
hsa_circ_0029426	hsa_miR_942	BAMBI
hsa_circ_0029426	hsa_miR_942	SYNPO2
hsa_circ_0029426	hsa_miR_942	TMEM63C
hsa_circ_0029426	hsa_miR_942	CCL16
hsa_circ_0029426	hsa_miR_942	UNC5D
hsa_circ_0029426	hsa_miR_942	DCAF8L1
hsa_circ_0029426	hsa_miR_942	ALX4
hsa_circ_0029426	hsa_miR_942	DCX
hsa_circ_0029426	hsa_miR_942	MAPK8IP3
hsa_circ_0029426	hsa_miR_942	SLC8A3
hsa_circ_0029426	hsa_miR_942	PDPN
hsa_circ_0029426	hsa_miR_942	NRN1
hsa_circ_0029426	hsa_miR_942	OTX2
hsa_circ_0029426	hsa_miR_942	ZNF536
hsa_circ_0029426	hsa_miR_942	NPNT
hsa_circ_0029426	hsa_miR_942	FAM71F1
hsa_circ_0029426	hsa_miR_942	TSLP
hsa_circ_0029426	hsa_miR_942	LGSN
hsa_circ_0029426	hsa_miR_942	NPAS2
hsa_circ_0029426	hsa_miR_942	SLC11A1
hsa_circ_0029426	hsa_miR_942	PI15
hsa_circ_0029426	hsa_miR_942	A2ML1
hsa_circ_0029426	hsa_miR_942	ERG
hsa_circ_0029426	hsa_miR_942	STXBP5L
hsa_circ_0029426	hsa_miR_942	DGKI
hsa_circ_0029426	hsa_miR_942	GREM1
hsa_circ_0029426	hsa_miR_942	AMOTL1
hsa_circ_0029426	hsa_miR_942	VGLL2
hsa_circ_0029426	hsa_miR_942	UGT3A1
hsa_circ_0029426	hsa_miR_942	CRY2
hsa_circ_0029426	hsa_miR_942	TNXB
hsa_circ_0029426	hsa_miR_942	A1CF
hsa_circ_0029426	hsa_miR_942	MEOX1
hsa_circ_0029426	hsa_miR_942	MRAS
hsa_circ_0029426	hsa_miR_942	SLCO1A2
hsa_circ_0029426	hsa_miR_942	TMEM92

hsa_circ_0029426	hsa_miR_942	NEUROD1
hsa_circ_0029426	hsa_miR_942	APLN
hsa_circ_0029426	hsa_miR_942	LDLR
hsa_circ_0029426	hsa_miR_942	ERBB4
hsa_circ_0029426	hsa_miR_942	GPX8
hsa_circ_0029426	hsa_miR_942	LRRN3
hsa_circ_0029426	hsa_miR_942	ACADL
hsa_circ_0029426	hsa_miR_942	KCNA4
hsa_circ_0029426	hsa_miR_942	CEACAM16
hsa_circ_0029426	hsa_miR_942	KCNJ10
hsa_circ_0029426	hsa_miR_942	RIC3
hsa_circ_0029426	hsa_miR_942	SPOCK2
hsa_circ_0029426	hsa_miR_942	ZNF366
hsa_circ_0029426	hsa_miR_942	OTUD1
hsa_circ_0029426	hsa_miR_942	PIK3R1
hsa_circ_0029426	hsa_miR_942	SYT7
hsa_circ_0029426	hsa_miR_942	TLL1
hsa_circ_0029426	hsa_miR_942	TRAF4
hsa_circ_0029426	hsa_miR_942	VASH1
hsa_circ_0029426	hsa_miR_942	PLEKHA8
hsa_circ_0029426	hsa_miR_942	HBEGF
hsa_circ_0029426	hsa_miR_942	MDGA2
hsa_circ_0029426	hsa_miR_942	HOXC13
hsa_circ_0029426	hsa_miR_942	SLC9A7
hsa_circ_0029426	hsa_miR_942	GFRA1
hsa_circ_0029426	hsa_miR_942	LRRC18
hsa_circ_0029426	hsa_miR_942	TPPP
hsa_circ_0029426	hsa_miR_942	ZNF322
hsa_circ_0029426	hsa_miR_942	SLC22A18AS
hsa_circ_0029426	hsa_miR_942	ZC3H12B
hsa_circ_0029426	hsa_miR_942	MAGEL2
hsa_circ_0029426	hsa_miR_942	LMOD1
hsa_circ_0029426	hsa_miR_942	PKP1
hsa_circ_0043256	hsa_miR_571	REG1A
hsa_circ_0043256	hsa_miR_571	LAMA1
hsa_circ_0043256	hsa_miR_571	KCNH7
hsa_circ_0043256	hsa_miR_571	CYP24A1
hsa_circ_0043256	hsa_miR_571	CDRT1
hsa_circ_0043256	hsa_miR_571	SLC1A6
hsa_circ_0043256	hsa_miR_571	KDM5B
hsa_circ_0043256	hsa_miR_571	FAM163A
hsa_circ_0043256	hsa_miR_571	KCNIP1
hsa_circ_0043256	hsa_miR_571	RFPL3
hsa_circ_0043256	hsa_miR_571	NECAB1
hsa_circ_0043256	hsa_miR_571	LINGO1
hsa_circ_0043256	hsa_miR_571	LMX1A
hsa_circ_0043256	hsa_miR_571	XDH
hsa_circ_0043256	hsa_miR_571	HECW1
hsa_circ_0043256	hsa_miR_571	KCNN3
hsa_circ_0043256	hsa_miR_571	SLC6A4
hsa_circ_0043256	hsa_miR_571	FLVCR2
hsa_circ_0043256	hsa_miR_571	PHKA1
hsa_circ_0043256	hsa_miR_571	RASSF8
hsa_circ_0043256	hsa_miR_571	SYNPO2
hsa_circ_0043256	hsa_miR_571	ZNF781
hsa_circ_0043256	hsa_miR_571	PKHD1
hsa_circ_0043256	hsa_miR_571	FAM189A1
hsa_circ_0043256	hsa_miR_571	FCGR3A

hsa_circ_0043256	hsa_miR_571	SLC39A11
hsa_circ_0043256	hsa_miR_571	CASP14
hsa_circ_0043256	hsa_miR_571	MYOZ1
hsa_circ_0043256	hsa_miR_571	ADARB2
hsa_circ_0043256	hsa_miR_571	TLR4
hsa_circ_0043256	hsa_miR_571	FCGR3B
hsa_circ_0043256	hsa_miR_571	CREG2
hsa_circ_0043256	hsa_miR_571	CHL1
hsa_circ_0043256	hsa_miR_571	LRRC32
hsa_circ_0043256	hsa_miR_571	SMUG1
hsa_circ_0043256	hsa_miR_571	GRID1
hsa_circ_0043256	hsa_miR_571	REG1B
hsa_circ_0043256	hsa_miR_571	SIX1
hsa_circ_0043256	hsa_miR_571	IBSP
hsa_circ_0043256	hsa_miR_571	ONECUT2
hsa_circ_0043256	hsa_miR_571	MMP19
hsa_circ_0043256	hsa_miR_571	QKI
hsa_circ_0043256	hsa_miR_571	EIF4E3
hsa_circ_0043256	hsa_miR_571	SLC18A2
hsa_circ_0043256	hsa_miR_571	KCNQ3
hsa_circ_0043256	hsa_miR_571	STMN2
hsa_circ_0043256	hsa_miR_571	BCL2L15
hsa_circ_0043256	hsa_miR_571	TMEM215
hsa_circ_0043256	hsa_miR_571	SLC28A3
hsa_circ_0043256	hsa_miR_571	MAOB
hsa_circ_0043256	hsa_miR_571	SLC16A6
hsa_circ_0043256	hsa_miR_571	CX3CL1
hsa_circ_0043256	hsa_miR_571	CCDC169
hsa_circ_0043256	hsa_miR_571	TYROBP
hsa_circ_0043256	hsa_miR_571	SIRPB2
hsa_circ_0043256	hsa_miR_571	DBF4B
hsa_circ_0043256	hsa_miR_571	PIRT
hsa_circ_0043256	hsa_miR_571	SEMA5A
hsa_circ_0043256	hsa_miR_571	ALDH3B2
hsa_circ_0043256	hsa_miR_571	FBXO32
hsa_circ_0043256	hsa_miR_571	SCAI
hsa_circ_0043256	hsa_miR_571	SLC5A9
hsa_circ_0043256	hsa_miR_571	FGF5
hsa_circ_0043256	hsa_miR_571	TMEM184A
hsa_circ_0043256	hsa_miR_571	SCN3B
hsa_circ_0043256	hsa_miR_571	FAM180B
hsa_circ_0043256	hsa_miR_571	TUBA4A
hsa_circ_0043256	hsa_miR_571	ALG3
hsa_circ_0043256	hsa_miR_571	GAS2L2
hsa_circ_0043256	hsa_miR_571	EPHB2
hsa_circ_0043256	hsa_miR_571	FRMPD4
hsa_circ_0043256	hsa_miR_571	GBP4
hsa_circ_0043256	hsa_miR_571	SOCS2
hsa_circ_0043256	hsa_miR_571	DYNAP
hsa_circ_0043256	hsa_miR_571	NETO1
hsa_circ_0043256	hsa_miR_571	AMPH
hsa_circ_0043256	hsa_miR_571	SPOCK1
hsa_circ_0043256	hsa_miR_571	RAB3B
hsa_circ_0043256	hsa_miR_571	CX3CR1
hsa_circ_0043256	hsa_miR_571	PMP22
hsa_circ_0043256	hsa_miR_571	CDC25C
hsa_circ_0043256	hsa_miR_571	FCRL4
hsa_circ_0043256	hsa_miR_571	GNPNAT1

hsa_circ_0043256	hsa_miR_571	CLSPN
hsa_circ_0043256	hsa_miR_571	ILF2
hsa_circ_0043256	hsa_miR_571	PCSK1
hsa_circ_0043256	hsa_miR_512-5p	HLTF
hsa_circ_0043256	hsa_miR_512-5p	STARD8
hsa_circ_0043256	hsa_miR_512-5p	TRHDE
hsa_circ_0043256	hsa_miR_512-5p	RIMS1
hsa_circ_0043256	hsa_miR_512-5p	MPPED1
hsa_circ_0043256	hsa_miR_512-5p	CAMK2B
hsa_circ_0043256	hsa_miR_512-5p	REG1A
hsa_circ_0043256	hsa_miR_512-5p	KCNC1
hsa_circ_0043256	hsa_miR_512-5p	KCNH5
hsa_circ_0043256	hsa_miR_512-5p	DUOX2
hsa_circ_0043256	hsa_miR_512-5p	DCC
hsa_circ_0043256	hsa_miR_512-5p	GPC5
hsa_circ_0043256	hsa_miR_512-5p	CCDC167
hsa_circ_0043256	hsa_miR_512-5p	ACP5
hsa_circ_0043256	hsa_miR_512-5p	LIN7A
hsa_circ_0043256	hsa_miR_512-5p	ZNF107
hsa_circ_0043256	hsa_miR_512-5p	PEAK1
hsa_circ_0043256	hsa_miR_512-5p	GJA1
hsa_circ_0043256	hsa_miR_512-5p	CTSV
hsa_circ_0043256	hsa_miR_512-5p	CENPL
hsa_circ_0043256	hsa_miR_512-5p	ZNF300
hsa_circ_0043256	hsa_miR_512-5p	TCF4
hsa_circ_0043256	hsa_miR_512-5p	LCT
hsa_circ_0043256	hsa_miR_512-5p	PPP1R16B
hsa_circ_0043256	hsa_miR_512-5p	SPRED3
hsa_circ_0043256	hsa_miR_512-5p	MAP10
hsa_circ_0043256	hsa_miR_512-5p	CYP2C18
hsa_circ_0043256	hsa_miR_512-5p	DNAJC12
hsa_circ_0043256	hsa_miR_512-5p	UNC5CL
hsa_circ_0043256	hsa_miR_512-5p	SYNPO2
hsa_circ_0043256	hsa_miR_512-5p	SERTM1
hsa_circ_0043256	hsa_miR_512-5p	CENPF
hsa_circ_0043256	hsa_miR_512-5p	HTR4
hsa_circ_0043256	hsa_miR_512-5p	NEUROD1
hsa_circ_0043256	hsa_miR_512-5p	ZNF474
hsa_circ_0043256	hsa_miR_512-5p	ZNF681
hsa_circ_0043256	hsa_miR_512-5p	PARPBP
hsa_circ_0043256	hsa_miR_512-5p	SEMA6A
hsa_circ_0043256	hsa_miR_512-5p	ZNF257
hsa_circ_0043256	hsa_miR_512-5p	ZNF460
hsa_circ_0043256	hsa_miR_512-5p	TK1
hsa_circ_0043256	hsa_miR_512-5p	TUBAL3
hsa_circ_0043256	hsa_miR_512-5p	AGPAT4
hsa_circ_0043256	hsa_miR_512-5p	FLT4
hsa_circ_0043256	hsa_miR_512-5p	SLCO2A1
hsa_circ_0043256	hsa_miR_512-5p	TMEM179
hsa_circ_0043256	hsa_miR_512-5p	CD300C
hsa_circ_0043256	hsa_miR_512-5p	CAPN5
hsa_circ_0043256	hsa_miR_512-5p	ZNF536
hsa_circ_0043256	hsa_miR_512-5p	PIK3R1
hsa_circ_0043256	hsa_miR_512-5p	CYP19A1
hsa_circ_0043256	hsa_miR_512-5p	AKAP7
hsa_circ_0043256	hsa_miR_512-5p	FAM83C
hsa_circ_0043256	hsa_miR_512-5p	C1orf226
hsa_circ_0043256	hsa_miR_512-5p	PCSK1

hsa_circ_0043256	hsa_miR_512-5p	ST8SIA2
hsa_circ_0043256	hsa_miR_512-5p	SCAI
hsa_circ_0043256	hsa_miR_512-5p	LRAT
hsa_circ_0043256	hsa_miR_512-5p	CHRM1
hsa_circ_0043256	hsa_miR_512-5p	DBF4
hsa_circ_0043256	hsa_miR_1303	AGMO
hsa_circ_0043256	hsa_miR_1303	AVL9
hsa_circ_0043256	hsa_miR_1303	TMEM151B
hsa_circ_0043256	hsa_miR_1303	VWC2
hsa_circ_0043256	hsa_miR_1303	FAM81A
hsa_circ_0043256	hsa_miR_1303	TET1
hsa_circ_0043256	hsa_miR_1303	KCTD12
hsa_circ_0043256	hsa_miR_1303	MYLK
hsa_circ_0043256	hsa_miR_1303	CLIC6
hsa_circ_0043256	hsa_miR_1303	COBL
hsa_circ_0043256	hsa_miR_1303	AGTR1
hsa_circ_0043256	hsa_miR_1303	SNX31
hsa_circ_0043256	hsa_miR_1303	CLDN18
hsa_circ_0043256	hsa_miR_1303	GALNT13
hsa_circ_0043256	hsa_miR_1303	EPHX4
hsa_circ_0043256	hsa_miR_1303	EGR2
hsa_circ_0043256	hsa_miR_1303	P2RY14
hsa_circ_0043256	hsa_miR_1303	ACADL
hsa_circ_0043256	hsa_miR_1303	AOC3
hsa_circ_0043256	hsa_miR_1303	PDE1C
hsa_circ_0043256	hsa_miR_1303	LSAMP
hsa_circ_0043256	hsa_miR_1303	NOVA1
hsa_circ_0043256	hsa_miR_1303	EFNA5
hsa_circ_0043256	hsa_miR_1303	RUNX2
hsa_circ_0043256	hsa_miR_1303	TNFAIP8L3
hsa_circ_0043256	hsa_miR_1303	ENPP2
hsa_circ_0043256	hsa_miR_1303	ZNF217
hsa_circ_0043256	hsa_miR_1303	SCN3B
hsa_circ_0043256	hsa_miR_1303	ADH1B
hsa_circ_0043256	hsa_miR_1303	NETO2
hsa_circ_0043256	hsa_miR_1303	MOB3B
hsa_circ_0043256	hsa_miR_1303	IGSF1
hsa_circ_0043256	hsa_miR_1303	CHEK1
hsa_circ_0043256	hsa_miR_1303	TUBA1A
hsa_circ_0043256	hsa_miR_1303	RNF182
hsa_circ_0043256	hsa_miR_1303	PI15
hsa_circ_0043256	hsa_miR_1303	CNTNAP2
hsa_circ_0043256	hsa_miR_1303	ELOVL6
hsa_circ_0043256	hsa_miR_1303	FOXR2
hsa_circ_0043256	hsa_miR_1303	ERVFRD-1
hsa_circ_0043256	hsa_miR_1303	UNC13C
hsa_circ_0043256	hsa_miR_1303	FAM9A
hsa_circ_0043256	hsa_miR_1303	FAM9C
hsa_circ_0043256	hsa_miR_1303	BOLL
hsa_circ_0043256	hsa_miR_1303	KHDRBS2
hsa_circ_0043256	hsa_miR_1303	TPRG1
hsa_circ_0043256	hsa_miR_1303	SLC24A4
hsa_circ_0043256	hsa_miR_1303	VGLL3
hsa_circ_0043256	hsa_miR_1303	GPSM2
hsa_circ_0043256	hsa_miR_1303	MCMDC2
hsa_circ_0043256	hsa_miR_1303	CRIM1
hsa_circ_0043256	hsa_miR_1303	LYVE1
hsa_circ_0043256	hsa_miR_1303	CALB1

hsa_circ_0043256	hsa_miR_1303	MAGI1
hsa_circ_0043256	hsa_miR_1303	SEZ6
hsa_circ_0043256	hsa_miR_1303	COL4A3
hsa_circ_0043256	hsa_miR_1303	CEACAM7
hsa_circ_0043256	hsa_miR_1303	HOXA10
hsa_circ_0043256	hsa_miR_1303	FGF11
hsa_circ_0043256	hsa_miR_1303	PPM1H
hsa_circ_0043256	hsa_miR_1303	SBK1
hsa_circ_0043256	hsa_miR_1303	ZBTB20
hsa_circ_0043256	hsa_miR_1303	RORA
hsa_circ_0043256	hsa_miR_1303	LY86
hsa_circ_0043256	hsa_miR_1303	MME
hsa_circ_0043256	hsa_miR_1303	GABRR2
hsa_circ_0043256	hsa_miR_1303	FCRL4
hsa_circ_0043256	hsa_miR_1303	PLEKHG4B
hsa_circ_0043256	hsa_miR_1303	NEGR1
hsa_circ_0043256	hsa_miR_1303	IFNK
hsa_circ_0043256	hsa_miR_1303	TLL1
hsa_circ_0043256	hsa_miR_1303	DGKI
hsa_circ_0043256	hsa_miR_1303	LPXN
hsa_circ_0043256	hsa_miR_1303	LILRB3
hsa_circ_0043256	hsa_miR_1303	KCNE1
hsa_circ_0043256	hsa_miR_1303	VSIG4
hsa_circ_0043256	hsa_miR_1303	KCNQ3
hsa_circ_0043256	hsa_miR_1303	ZNF117
hsa_circ_0043256	hsa_miR_1303	PNPLA5
hsa_circ_0043256	hsa_miR_1303	ADAM33
hsa_circ_0043256	hsa_miR_1303	CERS6
hsa_circ_0043256	hsa_miR_1303	CCNE2
hsa_circ_0043256	hsa_miR_1303	TMEM40
hsa_circ_0043256	hsa_miR_1303	APOL4
hsa_circ_0043256	hsa_miR_1303	CHST6
hsa_circ_0043256	hsa_miR_1303	BCL2L15
hsa_circ_0043256	hsa_miR_1303	ZBTB8B
hsa_circ_0043256	hsa_miR_1303	JAM2
hsa_circ_0043256	hsa_miR_1303	FUT3
hsa_circ_0043256	hsa_miR_1303	PTPRT
hsa_circ_0043256	hsa_miR_1303	ANKRD34B
hsa_circ_0043256	hsa_miR_1303	FAM9B
hsa_circ_0043256	hsa_miR_1303	CDC14A
hsa_circ_0043256	hsa_miR_1303	PRR11
hsa_circ_0043256	hsa_miR_1303	KCNJ16
hsa_circ_0043256	hsa_miR_1303	LRP2BP
hsa_circ_0043256	hsa_miR_1303	NAALADL2
hsa_circ_0043256	hsa_miR_1303	CHRNB1
hsa_circ_0043256	hsa_miR_1303	CLVS2
hsa_circ_0043256	hsa_miR_1303	ARHGAP23
hsa_circ_0043256	hsa_miR_1303	PAQR5
hsa_circ_0043256	hsa_miR_1303	LHX3
hsa_circ_0043256	hsa_miR_1303	C5orf49
hsa_circ_0043256	hsa_miR_1303	GLDN
hsa_circ_0043256	hsa_miR_1303	CEACAM6
hsa_circ_0043256	hsa_miR_1303	FRMD3
hsa_circ_0043256	hsa_miR_1303	CYP11B1
hsa_circ_0043256	hsa_miR_1303	PDK4
hsa_circ_0043256	hsa_miR_1303	INA
hsa_circ_0043256	hsa_miR_1303	CASP14
hsa_circ_0043256	hsa_miR_1303	DEPDC7

hsa_circ_0043256	hsa_miR_1303	CALCRL
hsa_circ_0043256	hsa_miR_1303	BRCA1
hsa_circ_0043256	hsa_miR_1303	ZC3H12B
hsa_circ_0043256	hsa_miR_1303	HOXA1
hsa_circ_0043256	hsa_miR_1303	ESRP1
hsa_circ_0043256	hsa_miR_1303	DDO
hsa_circ_0043256	hsa_miR_1303	AQP11
hsa_circ_0043256	hsa_miR_1303	JUNB
hsa_circ_0043256	hsa_miR_1303	ZIC1
hsa_circ_0043256	hsa_miR_1303	P2RY12
hsa_circ_0043256	hsa_miR_1303	CCR3
hsa_circ_0043256	hsa_miR_1303	DIXDC1
hsa_circ_0043256	hsa_miR_1303	RNF122
hsa_circ_0043256	hsa_miR_1303	A1CF
hsa_circ_0043256	hsa_miR_1303	MUCL1
hsa_circ_0043256	hsa_miR_1303	TCF21
hsa_circ_0043256	hsa_miR_1303	XRCC2
hsa_circ_0043256	hsa_miR_1303	RBPJL
hsa_circ_0043256	hsa_miR_1303	SLC6A11
hsa_circ_0043256	hsa_miR_1303	MTMR10
hsa_circ_0043256	hsa_miR_1303	PCDH9
hsa_circ_0043256	hsa_miR_1303	SRRM5
hsa_circ_0043256	hsa_miR_1303	TFAP2B
hsa_circ_0043256	hsa_miR_1303	NKD1
hsa_circ_0043256	hsa_miR_1303	ACE2
hsa_circ_0043256	hsa_miR_1303	LRAT
hsa_circ_0043256	hsa_miR_1303	PRLR
hsa_circ_0043256	hsa_miR_1303	PGC
hsa_circ_0043256	hsa_miR_1303	NMNAT2
hsa_circ_0043256	hsa_miR_1303	GPER1
hsa_circ_0043256	hsa_miR_1303	RBMS3
hsa_circ_0043256	hsa_miR_1303	NEBL
hsa_circ_0043256	hsa_miR_1303	NFKBIZ
hsa_circ_0043256	hsa_miR_1303	RUFY4
hsa_circ_0043256	hsa_miR_1303	DNER
hsa_circ_0043256	hsa_miR_1303	KPRP
hsa_circ_0043256	hsa_miR_1303	SLC2A1
hsa_circ_0043256	hsa_miR_1303	FAM72C
hsa_circ_0043256	hsa_miR_1303	POC1A
hsa_circ_0043256	hsa_miR_1303	SRSF12
hsa_circ_0043256	hsa_miR_1303	DSG1
hsa_circ_0043256	hsa_miR_1303	CCL28
hsa_circ_0043256	hsa_miR_1303	FAM72D
hsa_circ_0043256	hsa_miR_1303	COL4A4
hsa_circ_0043256	hsa_miR_1303	TARS2
hsa_circ_0043256	hsa_miR_1303	PXMP4
hsa_circ_0043256	hsa_miR_1303	ATRNL1
hsa_circ_0043256	hsa_miR_1303	ADAM28
hsa_circ_0043256	hsa_miR_1303	AMER3
hsa_circ_0043256	hsa_miR_1303	MMRN1
hsa_circ_0043256	hsa_miR_1303	IGFBPL1
hsa_circ_0043256	hsa_miR_1303	CYP1A2
hsa_circ_0043256	hsa_miR_1303	COL6A6
hsa_circ_0043256	hsa_miR_1303	IL12B
hsa_circ_0043256	hsa_miR_1303	MFSD9
hsa_circ_0043256	hsa_miR_1303	GRIK2
hsa_circ_0043256	hsa_miR_1303	PLOD2
hsa_circ_0043256	hsa_miR_1303	CPNE4

hsa_circ_0043256	hsa_miR_1303	MYCT1
hsa_circ_0043256	hsa_miR_1303	GRIN2A
hsa_circ_0043256	hsa_miR_1303	WNK3
hsa_circ_0043256	hsa_miR_1303	MEI4
hsa_circ_0043256	hsa_miR_1303	DIO2
hsa_circ_0043256	hsa_miR_1303	CLEC12B
hsa_circ_0043256	hsa_miR_1303	CYP2B6
hsa_circ_0043256	hsa_miR_1303	CNPY1
hsa_circ_0043256	hsa_miR_139-3p	PLD5
hsa_circ_0043256	hsa_miR_139-3p	C1QL4
hsa_circ_0043256	hsa_miR_139-3p	LMO2
hsa_circ_0043256	hsa_miR_139-3p	KIF18B
hsa_circ_0043256	hsa_miR_139-3p	RGS17
hsa_circ_0043256	hsa_miR_139-3p	CSMD2
hsa_circ_0043256	hsa_miR_139-3p	CIT
hsa_circ_0043256	hsa_miR_139-3p	RAD54B
hsa_circ_0043256	hsa_miR_139-3p	TTLL6
hsa_circ_0043256	hsa_miR_139-3p	C1orf105
hsa_circ_0043256	hsa_miR_139-3p	RAPGEF4
hsa_circ_0043256	hsa_miR_139-3p	SYT2
hsa_circ_0043256	hsa_miR_139-3p	ANGPT4
hsa_circ_0043256	hsa_miR_139-3p	SPRY4
hsa_circ_0043256	hsa_miR_139-3p	BCL2L14
hsa_circ_0043256	hsa_miR_139-3p	MMP16
hsa_circ_0043256	hsa_miR_139-3p	NPR3
hsa_circ_0043256	hsa_miR_139-3p	DUOX2
hsa_circ_0043256	hsa_miR_139-3p	SLC22A11
hsa_circ_0043256	hsa_miR_139-3p	SH2D3C
hsa_circ_0043256	hsa_miR_139-3p	OLFML2A
hsa_circ_0043256	hsa_miR_139-3p	EFEMP1
hsa_circ_0043256	hsa_miR_139-3p	SIRPA
hsa_circ_0043256	hsa_miR_139-3p	ATCAY
hsa_circ_0043256	hsa_miR_139-3p	NEGR1
hsa_circ_0043256	hsa_miR_139-3p	VGLL2
hsa_circ_0043256	hsa_miR_139-3p	SULT4A1
hsa_circ_0043256	hsa_miR_139-3p	TOM1L2
hsa_circ_0043256	hsa_miR_139-3p	CD177
hsa_circ_0043256	hsa_miR_139-3p	OPTC
hsa_circ_0043256	hsa_miR_139-3p	SDK1
hsa_circ_0043256	hsa_miR_139-3p	COL6A3
hsa_circ_0043256	hsa_miR_139-3p	CABLES2
hsa_circ_0043256	hsa_miR_139-3p	SLC2A2
hsa_circ_0043256	hsa_miR_139-3p	LHX6
hsa_circ_0043256	hsa_miR_139-3p	DPP6
hsa_circ_0043256	hsa_miR_139-3p	CDH10
hsa_circ_0043256	hsa_miR_139-3p	PRDM9
hsa_circ_0043256	hsa_miR_139-3p	DENND2C
hsa_circ_0043256	hsa_miR_139-3p	PACSIN1
hsa_circ_0043256	hsa_miR_139-3p	HOXD13
hsa_circ_0043256	hsa_miR_139-3p	SH2D5
hsa_circ_0043256	hsa_miR_139-3p	NFASC
hsa_circ_0043256	hsa_miR_556-5p	IL18R1
hsa_circ_0043256	hsa_miR_556-5p	BCL2L2-PABPN1
hsa_circ_0043256	hsa_miR_556-5p	COLEC10
hsa_circ_0043256	hsa_miR_556-5p	OSR2
hsa_circ_0043256	hsa_miR_556-5p	KLF6
hsa_circ_0043256	hsa_miR_556-5p	RIMS4
hsa_circ_0043256	hsa_miR_556-5p	PIP5KL1

hsa_circ_0043256	hsa_miR_556-5p	HOXA1
hsa_circ_0043256	hsa_miR_556-5p	KCNJ15
hsa_circ_0043256	hsa_miR_556-5p	LGALSL
hsa_circ_0043256	hsa_miR_556-5p	LRP2BP
hsa_circ_0043256	hsa_miR_556-5p	HTR2C
hsa_circ_0043256	hsa_miR_556-5p	RASSF6
hsa_circ_0043256	hsa_miR_556-5p	AGTR2
hsa_circ_0043256	hsa_miR_556-5p	NDC1
hsa_circ_0043256	hsa_miR_556-5p	WFIKKN2
hsa_circ_0043256	hsa_miR_556-5p	BCL2L14
hsa_circ_0043256	hsa_miR_556-5p	CERS6
hsa_circ_0043256	hsa_miR_556-5p	NUP210
hsa_circ_0043256	hsa_miR_556-5p	HS3ST3A1
hsa_circ_0043256	hsa_miR_556-5p	CYBRD1
hsa_circ_0043256	hsa_miR_556-5p	SEMA5A
hsa_circ_0043256	hsa_miR_556-5p	B3GNT3
hsa_circ_0043256	hsa_miR_556-5p	ZNF322
hsa_circ_0043256	hsa_miR_556-5p	FUT9
hsa_circ_0043256	hsa_miR_556-5p	RUNX1T1
hsa_circ_0043256	hsa_miR_556-5p	NRIP3
hsa_circ_0043256	hsa_miR_556-5p	LIFR
hsa_circ_0043256	hsa_miR_556-5p	NEUROD4
hsa_circ_0043256	hsa_miR_556-5p	A1CF
hsa_circ_0043256	hsa_miR_556-5p	CYP4F3
hsa_circ_0043256	hsa_miR_556-5p	MYOCD
hsa_circ_0043256	hsa_miR_556-5p	PTPRD
hsa_circ_0043256	hsa_miR_556-5p	MAGEA9
hsa_circ_0043256	hsa_miR_556-5p	MAGEA9B
hsa_circ_0043256	hsa_miR_556-5p	USH1C
hsa_circ_0043256	hsa_miR_556-5p	ZNF217
hsa_circ_0043256	hsa_miR_556-5p	KCNJ1
hsa_circ_0043256	hsa_miR_556-5p	PRF1
hsa_circ_0043256	hsa_miR_556-5p	EDDM3A
hsa_circ_0043256	hsa_miR_556-5p	EDDM3B
hsa_circ_0043256	hsa_miR_556-5p	RIC3
hsa_circ_0043256	hsa_miR_556-5p	IGSF1
hsa_circ_0043256	hsa_miR_556-5p	LYZ
hsa_circ_0043256	hsa_miR_556-5p	GNRHR
hsa_circ_0043256	hsa_miR_556-5p	CLVS2
hsa_circ_0043256	hsa_miR_556-5p	VGLL3
hsa_circ_0043256	hsa_miR_556-5p	CYP4B1
hsa_circ_0043256	hsa_miR_556-5p	AKR1C2
hsa_circ_0043256	hsa_miR_556-5p	ENTPD7
hsa_circ_0043256	hsa_miR_556-5p	LRIG3
hsa_circ_0043256	hsa_miR_556-5p	RGS5
hsa_circ_0043256	hsa_miR_556-5p	SLC5A6
hsa_circ_0043256	hsa_miR_556-5p	LRAT
hsa_circ_0043256	hsa_miR_556-5p	ALDH1L2
hsa_circ_0043256	hsa_miR_556-5p	FAM124B
hsa_circ_0043256	hsa_miR_556-5p	SUV39H2
hsa_circ_0043256	hsa_miR_556-5p	PARD3B
hsa_circ_0043256	hsa_miR_556-5p	CAV2
hsa_circ_0043256	hsa_miR_577	TFAP2A
hsa_circ_0043256	hsa_miR_577	RBFOX1
hsa_circ_0043256	hsa_miR_577	AMER2
hsa_circ_0043256	hsa_miR_577	PTF1A
hsa_circ_0043256	hsa_miR_577	CCNE2
hsa_circ_0043256	hsa_miR_577	RTN4RL2

hsa_circ_0043256	hsa_miR_577	GPR65
hsa_circ_0043256	hsa_miR_577	GRIN2A
hsa_circ_0043256	hsa_miR_577	PRKAA2
hsa_circ_0043256	hsa_miR_577	SLC7A11
hsa_circ_0043256	hsa_miR_577	IGSF11
hsa_circ_0043256	hsa_miR_577	SLC27A6
hsa_circ_0043256	hsa_miR_577	CYBRD1
hsa_circ_0043256	hsa_miR_577	ADRA1A
hsa_circ_0043256	hsa_miR_577	RORA
hsa_circ_0043256	hsa_miR_577	SKP2
hsa_circ_0043256	hsa_miR_577	IGF2BP1
hsa_circ_0043256	hsa_miR_577	RBP4
hsa_circ_0043256	hsa_miR_577	HOXA1
hsa_circ_0043256	hsa_miR_577	DACH1
hsa_circ_0043256	hsa_miR_577	TENM1
hsa_circ_0043256	hsa_miR_577	AQP4
hsa_circ_0043256	hsa_miR_577	SPDYE1
hsa_circ_0043256	hsa_miR_577	FAM71F1
hsa_circ_0043256	hsa_miR_577	SLIT3
hsa_circ_0043256	hsa_miR_577	MLLT11
hsa_circ_0043256	hsa_miR_577	SH3GL2
hsa_circ_0043256	hsa_miR_577	KCNJ13
hsa_circ_0043256	hsa_miR_577	CRHR1
hsa_circ_0043256	hsa_miR_577	SAMHD1
hsa_circ_0043256	hsa_miR_577	PRDM12
hsa_circ_0043256	hsa_miR_577	SLC2A14
hsa_circ_0043256	hsa_miR_577	APLN
hsa_circ_0043256	hsa_miR_577	DENND2A
hsa_circ_0043256	hsa_miR_577	NCKAP5
hsa_circ_0043256	hsa_miR_577	UGT3A1
hsa_circ_0043256	hsa_miR_577	LYPD6
hsa_circ_0043256	hsa_miR_577	RANBP3L
hsa_circ_0043256	hsa_miR_577	DPP10
hsa_circ_0043256	hsa_miR_577	ADAMTSL3
hsa_circ_0043256	hsa_miR_577	SDK1
hsa_circ_0043256	hsa_miR_577	KCNJ10
hsa_circ_0043256	hsa_miR_577	DCX
hsa_circ_0043256	hsa_miR_577	TRHDE
hsa_circ_0043256	hsa_miR_577	TFPI2
hsa_circ_0043256	hsa_miR_577	VGLL3
hsa_circ_0043256	hsa_miR_577	SALL4
hsa_circ_0043256	hsa_miR_577	TFAP2B
hsa_circ_0043256	hsa_miR_577	EPHA5
hsa_circ_0043256	hsa_miR_577	OSR2
hsa_circ_0043256	hsa_miR_577	CAV1
hsa_circ_0043256	hsa_miR_577	ZEB2
hsa_circ_0043256	hsa_miR_577	GLB1L3
hsa_circ_0043256	hsa_miR_577	PARP1
hsa_circ_0043256	hsa_miR_577	SPTBN1
hsa_circ_0043256	hsa_miR_577	DGKI
hsa_circ_0043256	hsa_miR_577	SHMT2
hsa_circ_0043256	hsa_miR_577	ZYG11A
hsa_circ_0043256	hsa_miR_577	SEMA5A
hsa_circ_0043256	hsa_miR_577	TESC
hsa_circ_0043256	hsa_miR_577	CALCRL
hsa_circ_0043256	hsa_miR_577	MCHR2
hsa_circ_0043256	hsa_miR_577	RBMS3
hsa_circ_0043256	hsa_miR_577	FRMPD4

hsa_circ_0043256	hsa_miR_577	BMPR2
hsa_circ_0043256	hsa_miR_577	GRIA4
hsa_circ_0043256	hsa_miR_577	ZSCAN2
hsa_circ_0043256	hsa_miR_577	KCNA4
hsa_circ_0043256	hsa_miR_577	PLCL1
hsa_circ_0043256	hsa_miR_577	GUCY1A2
hsa_circ_0043256	hsa_miR_577	SYCP2
hsa_circ_0043256	hsa_miR_577	PIWIL1
hsa_circ_0043256	hsa_miR_577	TAF7L
hsa_circ_0043256	hsa_miR_577	GFPT1
hsa_circ_0043256	hsa_miR_577	GKN2
hsa_circ_0043256	hsa_miR_577	LHFPL3
hsa_circ_0043256	hsa_miR_577	ITGB8
hsa_circ_0043256	hsa_miR_577	FGG
hsa_circ_0043256	hsa_miR_577	STYK1
hsa_circ_0043256	hsa_miR_577	SOX9
hsa_circ_0043256	hsa_miR_577	PPM1H
hsa_circ_0043256	hsa_miR_577	GRIK4
hsa_circ_0043256	hsa_miR_577	UGT2A3
hsa_circ_0043256	hsa_miR_577	SYT16
hsa_circ_0043256	hsa_miR_577	MAFF
hsa_circ_0043256	hsa_miR_577	CNOT11
hsa_circ_0043256	hsa_miR_577	KLF4
hsa_circ_0043256	hsa_miR_577	PXDC1
hsa_circ_0043256	hsa_miR_577	TMEM61
hsa_circ_0043256	hsa_miR_577	SDC2
hsa_circ_0043256	hsa_miR_577	LRRN1
hsa_circ_0043256	hsa_miR_577	RAB11FIP1
hsa_circ_0043256	hsa_miR_577	TMEM145
hsa_circ_0043256	hsa_miR_577	KIF20B
hsa_circ_0043256	hsa_miR_577	DAZL
hsa_circ_0043256	hsa_miR_577	FTHL17
hsa_circ_0043256	hsa_miR_577	CLEC12B
hsa_circ_0043256	hsa_miR_577	DCHS2
hsa_circ_0043256	hsa_miR_577	MTFR1
hsa_circ_0043256	hsa_miR_577	BDNF
hsa_circ_0043256	hsa_miR_577	DMC1
hsa_circ_0043256	hsa_miR_577	KCNJ16
hsa_circ_0043256	hsa_miR_577	LIN28B
hsa_circ_0043256	hsa_miR_577	RGS7
hsa_circ_0043256	hsa_miR_577	CDHR3
hsa_circ_0043256	hsa_miR_577	LFNG
hsa_circ_0043256	hsa_miR_577	CYBB
hsa_circ_0043256	hsa_miR_577	UNC5D
hsa_circ_0043256	hsa_miR_577	MYLK
hsa_circ_0043256	hsa_miR_577	MAGEB6
hsa_circ_0043256	hsa_miR_577	SLC16A7
hsa_circ_0043256	hsa_miR_577	FABP7
hsa_circ_0043256	hsa_miR_577	OTP
hsa_circ_0043256	hsa_miR_577	TCTEX1D1
hsa_circ_0043256	hsa_miR_577	CTSE
hsa_circ_0043256	hsa_miR_577	LRP1B
hsa_circ_0043256	hsa_miR_577	FOXF1
hsa_circ_0043256	hsa_miR_577	ONECUT2
hsa_circ_0043256	hsa_miR_577	KLF9
hsa_circ_0043256	hsa_miR_577	ATP2B2
hsa_circ_0043256	hsa_miR_577	QKI
hsa_circ_0043256	hsa_miR_577	OTX1

hsa_circ_0043256	hsa_miR_577	TPRG1
hsa_circ_0043256	hsa_miR_577	FGF5
hsa_circ_0043256	hsa_miR_577	BARD1
hsa_circ_0043256	hsa_miR_577	CDC14A
hsa_circ_0043256	hsa_miR_577	ATRNL1
hsa_circ_0043256	hsa_miR_577	RALGPS2
hsa_circ_0043256	hsa_miR_577	GABRA4
hsa_circ_0043256	hsa_miR_577	MYOCD
hsa_circ_0043256	hsa_miR_577	FLVCR1
hsa_circ_0043256	hsa_miR_577	SPP1
hsa_circ_0043256	hsa_miR_577	LAMP3
hsa_circ_0043256	hsa_miR_577	MEI4
hsa_circ_0043256	hsa_miR_577	ADAMTS16
hsa_circ_0043256	hsa_miR_577	PSAT1
hsa_circ_0043256	hsa_miR_577	TMEM215
hsa_circ_0043256	hsa_miR_577	RGS5
hsa_circ_0043256	hsa_miR_577	TOX3
hsa_circ_0043256	hsa_miR_577	SECISBP2L
hsa_circ_0043256	hsa_miR_577	CELF2
hsa_circ_0043256	hsa_miR_577	CSMD2
hsa_circ_0043256	hsa_miR_577	NRSN1
hsa_circ_0043256	hsa_miR_577	SOX7
hsa_circ_0043256	hsa_miR_577	TMEM45A
hsa_circ_0043256	hsa_miR_577	IQSEC3
hsa_circ_0043256	hsa_miR_577	RNF103-CHMP3
hsa_circ_0043256	hsa_miR_577	CYP27C1
hsa_circ_0043256	hsa_miR_577	RGS13
hsa_circ_0043256	hsa_miR_577	ZSCAN4
hsa_circ_0043256	hsa_miR_577	DNER
hsa_circ_0043256	hsa_miR_577	AKR1C4
hsa_circ_0043256	hsa_miR_577	FAM169A
hsa_circ_0043256	hsa_miR_577	SLC25A13
hsa_circ_0043256	hsa_miR_577	MTHFD2
hsa_circ_0043256	hsa_miR_577	ZBTB20
hsa_circ_0043256	hsa_miR_577	SYNPO2
hsa_circ_0043256	hsa_miR_577	AGTPBP1
hsa_circ_0043256	hsa_miR_577	GLIPR1L2
hsa_circ_0043256	hsa_miR_577	SCD5
hsa_circ_0043256	hsa_miR_577	ANGPTL3
hsa_circ_0043256	hsa_miR_577	ID4
hsa_circ_0043256	hsa_miR_577	RIMS2
hsa_circ_0043256	hsa_miR_577	LHX2
hsa_circ_0043256	hsa_miR_577	NEGR1
hsa_circ_0043256	hsa_miR_577	TOP2A
hsa_circ_0043256	hsa_miR_577	ZNF781
hsa_circ_0043256	hsa_miR_577	CPN2
hsa_circ_0043256	hsa_miR_577	LHCGR
hsa_circ_0043256	hsa_miR_577	RSPO4
hsa_circ_0043256	hsa_miR_577	ST18
hsa_circ_0043256	hsa_miR_577	BRIX1
hsa_circ_0043256	hsa_miR_577	TMTC1
hsa_circ_0043256	hsa_miR_577	KCNB1
hsa_circ_0043256	hsa_miR_577	DEPTOR
hsa_circ_0043256	hsa_miR_577	C2CD4A
hsa_circ_0043256	hsa_miR_577	GALNT4
hsa_circ_0043256	hsa_miR_577	DOCK4
hsa_circ_0043256	hsa_miR_577	KLF6
hsa_circ_0043256	hsa_miR_577	CHGA

hsa_circ_0043256	hsa_miR_577	G6PC
hsa_circ_0043256	hsa_miR_577	TOX2
hsa_circ_0043256	hsa_miR_577	RTKN2
hsa_circ_0043256	hsa_miR_577	PGM2L1
hsa_circ_0043256	hsa_miR_577	PGR
hsa_circ_0043256	hsa_miR_577	TRIM49
hsa_circ_0043256	hsa_miR_577	PAX6
hsa_circ_0043256	hsa_miR_577	SLC5A12
hsa_circ_0043256	hsa_miR_577	RASSF8
hsa_circ_0043256	hsa_miR_577	PDE8B
hsa_circ_0043256	hsa_miR_577	P2RY1
hsa_circ_0043256	hsa_miR_577	CCSER1
hsa_circ_0043256	hsa_miR_577	METTL7A
hsa_circ_0043256	hsa_miR_577	FURIN
hsa_circ_0043256	hsa_miR_577	MPP6
hsa_circ_0043256	hsa_miR_577	BRIP1
hsa_circ_0043256	hsa_miR_577	MMP16
hsa_circ_0043256	hsa_miR_577	ANGPTL7
hsa_circ_0043256	hsa_miR_577	BCL6B
hsa_circ_0043256	hsa_miR_577	ST8SIA3
hsa_circ_0043256	hsa_miR_577	RBM46
hsa_circ_0043256	hsa_miR_577	ZNF587
hsa_circ_0043256	hsa_miR_577	ARAP3
hsa_circ_0043256	hsa_miR_577	SIX1
hsa_circ_0043256	hsa_miR_577	SLC25A2
hsa_circ_0043256	hsa_miR_577	LDHA
hsa_circ_0043256	hsa_miR_577	WNK3
hsa_circ_0043256	hsa_miR_577	PKHD1
hsa_circ_0043256	hsa_miR_577	PROK2
hsa_circ_0043256	hsa_miR_577	SLIT2
hsa_circ_0043256	hsa_miR_577	RNF222
hsa_circ_0043256	hsa_miR_577	TACC1
hsa_circ_0043256	hsa_miR_577	TNFSF4
hsa_circ_0043256	hsa_miR_577	NAALADL2
hsa_circ_0043256	hsa_miR_577	ZWILCH
hsa_circ_0043256	hsa_miR_577	NRG1
hsa_circ_0043256	hsa_miR_577	LIFR
hsa_circ_0043256	hsa_miR_577	SLC9A7
hsa_circ_0043256	hsa_miR_577	FERMT1
hsa_circ_0043256	hsa_miR_577	LMO7
hsa_circ_0043256	hsa_miR_577	SRD5A1
hsa_circ_0043256	hsa_miR_577	OGN
hsa_circ_0043256	hsa_miR_577	ASPH
hsa_circ_0043256	hsa_miR_577	NEBL
hsa_circ_0043256	hsa_miR_577	TBX18
hsa_circ_0043256	hsa_miR_577	FAM83H
hsa_circ_0043256	hsa_miR_577	DAB2IP
hsa_circ_0043256	hsa_miR_577	AK7
hsa_circ_0043256	hsa_miR_577	TBX4
hsa_circ_0043256	hsa_miR_577	KLHL17
hsa_circ_0043256	hsa_miR_577	CCDC34
hsa_circ_0043256	hsa_miR_577	TMEM220
hsa_circ_0043256	hsa_miR_577	CPNE4
hsa_circ_0043256	hsa_miR_577	SPOCK3
hsa_circ_0043256	hsa_miR_577	KDM5B
hsa_circ_0043256	hsa_miR_577	CYP4F2
hsa_circ_0043256	hsa_miR_577	TUBB4A
hsa_circ_0043256	hsa_miR_577	EYS

hsa_circ_0043256	hsa_miR_577	TUBG1
hsa_circ_0043256	hsa_miR_577	DIO2
hsa_circ_0043256	hsa_miR_577	SNTG1
hsa_circ_0043256	hsa_miR_577	PLAGL1
hsa_circ_0043256	hsa_miR_577	SCIN
hsa_circ_0043256	hsa_miR_577	TRIM15
hsa_circ_0043256	hsa_miR_577	SCEL
hsa_circ_0043256	hsa_miR_577	LGR4
hsa_circ_0043256	hsa_miR_577	LYVE1
hsa_circ_0043256	hsa_miR_577	GNRHR
hsa_circ_0043256	hsa_miR_577	LCOR
hsa_circ_0043256	hsa_miR_577	NTN4
hsa_circ_0043256	hsa_miR_577	HS3ST3A1
hsa_circ_0043256	hsa_miR_577	SLCO1A2
hsa_circ_0043256	hsa_miR_577	RAB25
hsa_circ_0043256	hsa_miR_577	RGS17
hsa_circ_0043256	hsa_miR_577	SPRR2D
hsa_circ_0043256	hsa_miR_1206	SNTG1
hsa_circ_0043256	hsa_miR_1206	TMEFF1
hsa_circ_0043256	hsa_miR_1206	KCND2
hsa_circ_0043256	hsa_miR_1206	SEMA6A
hsa_circ_0043256	hsa_miR_1206	KCNQ5
hsa_circ_0043256	hsa_miR_1206	HOXA1
hsa_circ_0043256	hsa_miR_1206	LRP1B
hsa_circ_0043256	hsa_miR_1206	NDNF
hsa_circ_0043256	hsa_miR_1206	FUT8
hsa_circ_0043256	hsa_miR_1206	NR5A2
hsa_circ_0043256	hsa_miR_1206	DSCC1
hsa_circ_0043256	hsa_miR_1206	DOK6
hsa_circ_0043256	hsa_miR_1206	SKA3
hsa_circ_0043256	hsa_miR_1206	THRB
hsa_circ_0043256	hsa_miR_1206	KLHL32
hsa_circ_0043256	hsa_miR_1206	LRRC31
hsa_circ_0043256	hsa_miR_1206	KCNB2
hsa_circ_0043256	hsa_miR_1206	TIMP3
hsa_circ_0043256	hsa_miR_1206	SHISA3
hsa_circ_0043256	hsa_miR_1206	TPBG
hsa_circ_0043256	hsa_miR_1206	VSTM4
hsa_circ_0043256	hsa_miR_1206	GRIK2
hsa_circ_0043256	hsa_miR_1206	FAT1
hsa_circ_0043256	hsa_miR_1206	HTR4
hsa_circ_0043256	hsa_miR_1206	RHNO1
hsa_circ_0043256	hsa_miR_1206	PHEX
hsa_circ_0043256	hsa_miR_1206	NTN4
hsa_circ_0043256	hsa_miR_1206	TLL2
hsa_circ_0043256	hsa_miR_1206	QKI
hsa_circ_0043256	hsa_miR_1206	NIPSNAP1
hsa_circ_0043256	hsa_miR_1206	OPRK1
hsa_circ_0043256	hsa_miR_1206	GCLM
hsa_circ_0043256	hsa_miR_1206	ADAMTS19
hsa_circ_0043256	hsa_miR_1206	IGF2
hsa_circ_0043256	hsa_miR_1206	PROS1
hsa_circ_0043256	hsa_miR_1206	FAXC
hsa_circ_0043256	hsa_miR_1206	CCDC141
hsa_circ_0043256	hsa_miR_1206	PHF21B
hsa_circ_0043256	hsa_miR_1206	ABCA10
hsa_circ_0043256	hsa_miR_1206	SASH1
hsa_circ_0043256	hsa_miR_1206	SORBS1

hsa_circ_0043256	hsa_miR_1206	EPHA7
hsa_circ_0043256	hsa_miR_1206	TIMM8A
hsa_circ_0043256	hsa_miR_1206	ZBTB20
hsa_circ_0043256	hsa_miR_1206	MRAS
hsa_circ_0043256	hsa_miR_1206	SLC27A2
hsa_circ_0043256	hsa_miR_1206	FUT9
hsa_circ_0043256	hsa_miR_1206	FAM216B
hsa_circ_0043256	hsa_miR_1206	CCBE1
hsa_circ_0043256	hsa_miR_1206	CHML
hsa_circ_0043256	hsa_miR_1206	GDAP1L1
hsa_circ_0043256	hsa_miR_1206	F2RL2
hsa_circ_0043256	hsa_miR_1206	ALDH1L2
hsa_circ_0043256	hsa_miR_1206	BARD1
hsa_circ_0043256	hsa_miR_1206	NKAIN3
hsa_circ_0043256	hsa_miR_1206	ZNF367
hsa_circ_0043256	hsa_miR_1206	RIPPLY3
hsa_circ_0043256	hsa_miR_1206	RBMS3
hsa_circ_0043256	hsa_miR_1206	SHROOM4
hsa_circ_0043256	hsa_miR_1206	CAMK2B
hsa_circ_0043256	hsa_miR_1206	C1QTNF7
hsa_circ_0043256	hsa_miR_1206	SSTR1
hsa_circ_0043256	hsa_miR_1206	NTM
hsa_circ_0043256	hsa_miR_1206	EGLN3
hsa_circ_0043256	hsa_miR_1206	SYTL5
hsa_circ_0043256	hsa_miR_1206	DUSP4
hsa_circ_0043256	hsa_miR_1206	SCN3A
hsa_circ_0043256	hsa_miR_1206	ROR1
hsa_circ_0043256	hsa_miR_1206	SEMA3A
hsa_circ_0043256	hsa_miR_1206	RASSF6
hsa_circ_0043256	hsa_miR_1206	KCNB1
hsa_circ_0043256	hsa_miR_1206	PEAR1
hsa_circ_0043256	hsa_miR_1206	CDH10
hsa_circ_0043256	hsa_miR_1206	GUCY1A2
hsa_circ_0043256	hsa_miR_1206	MITF
hsa_circ_0043256	hsa_miR_1206	NOVA1
hsa_circ_0043256	hsa_miR_1206	RS1
hsa_circ_0043256	hsa_miR_1206	SLC5A7
hsa_circ_0043256	hsa_miR_1206	KCNK15
hsa_circ_0043256	hsa_miR_1206	CBR1
hsa_circ_0043256	hsa_miR_1206	VSX2
hsa_circ_0043256	hsa_miR_1206	PRDM5
hsa_circ_0043256	hsa_miR_1206	FRMD3
hsa_circ_0043256	hsa_miR_1206	TFF3
hsa_circ_0043256	hsa_miR_1206	KCNJ1
hsa_circ_0043256	hsa_miR_1206	RASSF8
hsa_circ_0043256	hsa_miR_1206	ROBO2
hsa_circ_0043256	hsa_miR_1206	RASSF2
hsa_circ_0043256	hsa_miR_1206	SLC2A2
hsa_circ_0043256	hsa_miR_1206	RD3
hsa_circ_0043256	hsa_miR_1206	IL5RA
hsa_circ_0043256	hsa_miR_1206	SCAI
hsa_circ_0043256	hsa_miR_1206	CREG2
hsa_circ_0043256	hsa_miR_1206	NSG2
hsa_circ_0043256	hsa_miR_1206	TMEM132C
hsa_circ_0043256	hsa_miR_1206	CHL1
hsa_circ_0043256	hsa_miR_1206	C20orf197
hsa_circ_0043256	hsa_miR_576-5p	NR4A3
hsa_circ_0043256	hsa_miR_576-5p	FUT9

hsa_circ_0043256	hsa_miR_576-5p	NECAB1
hsa_circ_0043256	hsa_miR_576-5p	KLF10
hsa_circ_0043256	hsa_miR_576-5p	FGF2
hsa_circ_0043256	hsa_miR_576-5p	AQP4
hsa_circ_0043256	hsa_miR_576-5p	RGS5
hsa_circ_0043256	hsa_miR_576-5p	BOLL
hsa_circ_0043256	hsa_miR_576-5p	IGDCC4
hsa_circ_0043256	hsa_miR_576-5p	KCNQ5
hsa_circ_0043256	hsa_miR_576-5p	ASPH
hsa_circ_0043256	hsa_miR_576-5p	RBMS3
hsa_circ_0043256	hsa_miR_576-5p	SIX2
hsa_circ_0043256	hsa_miR_576-5p	ZEB1
hsa_circ_0043256	hsa_miR_576-5p	SLC5A12
hsa_circ_0043256	hsa_miR_576-5p	CNTNAP2
hsa_circ_0043256	hsa_miR_576-5p	WDR72
hsa_circ_0043256	hsa_miR_576-5p	EFNA5
hsa_circ_0043256	hsa_miR_576-5p	HOOK1
hsa_circ_0043256	hsa_miR_576-5p	SCAI
hsa_circ_0043256	hsa_miR_576-5p	FLI1
hsa_circ_0043256	hsa_miR_576-5p	ARHGAP11A
hsa_circ_0043256	hsa_miR_576-5p	HPRT1
hsa_circ_0043256	hsa_miR_576-5p	FAR2
hsa_circ_0043256	hsa_miR_576-5p	PCDHB2
hsa_circ_0043256	hsa_miR_576-5p	CYYR1
hsa_circ_0043256	hsa_miR_576-5p	MMP20
hsa_circ_0043256	hsa_miR_576-5p	GAB1
hsa_circ_0043256	hsa_miR_576-5p	FAXC
hsa_circ_0043256	hsa_miR_576-5p	ARMC3
hsa_circ_0043256	hsa_miR_576-5p	AK7
hsa_circ_0043256	hsa_miR_576-5p	ITGB8
hsa_circ_0043256	hsa_miR_576-5p	ZFHX4
hsa_circ_0043256	hsa_miR_576-5p	GCLM
hsa_circ_0043256	hsa_miR_576-5p	ONECUT2
hsa_circ_0043256	hsa_miR_576-5p	GRM3
hsa_circ_0043256	hsa_miR_576-5p	ANKRD29
hsa_circ_0043256	hsa_miR_576-5p	PEX5L
hsa_circ_0043256	hsa_miR_576-5p	GRIN2A
hsa_circ_0043256	hsa_miR_576-5p	SOHLH2
hsa_circ_0043256	hsa_miR_576-5p	LDB2
hsa_circ_0043256	hsa_miR_576-5p	SLC7A11
hsa_circ_0043256	hsa_miR_576-5p	HS3ST3A1
hsa_circ_0043256	hsa_miR_576-5p	FAM169A
hsa_circ_0043256	hsa_miR_576-5p	PLCL1
hsa_circ_0043256	hsa_miR_576-5p	TMEM196
hsa_circ_0043256	hsa_miR_576-5p	KCTD4
hsa_circ_0043256	hsa_miR_576-5p	NCKAP5
hsa_circ_0043256	hsa_miR_576-5p	CDCA4
hsa_circ_0043256	hsa_miR_576-5p	MSX2
hsa_circ_0043256	hsa_miR_576-5p	SLC16A7
hsa_circ_0043256	hsa_miR_576-5p	CAV1
hsa_circ_0043256	hsa_miR_576-5p	KCNA4
hsa_circ_0043256	hsa_miR_576-5p	AVL9
hsa_circ_0043256	hsa_miR_576-5p	TRDN
hsa_circ_0043256	hsa_miR_576-5p	FSTL5
hsa_circ_0043256	hsa_miR_576-5p	BMP2
hsa_circ_0043256	hsa_miR_576-5p	PLEKHA8
hsa_circ_0043256	hsa_miR_576-5p	DCLK1
hsa_circ_0043256	hsa_miR_576-5p	PCDH10

hsa_circ_0043256	hsa_miR_576-5p	EPHX4
hsa_circ_0043256	hsa_miR_576-5p	SLCO4C1
hsa_circ_0043256	hsa_miR_576-5p	SUMO4
hsa_circ_0043256	hsa_miR_576-5p	SI
hsa_circ_0043256	hsa_miR_576-5p	EGF
hsa_circ_0043256	hsa_miR_576-5p	SLC9A4
hsa_circ_0043256	hsa_miR_576-5p	SYTL2
hsa_circ_0043256	hsa_miR_576-5p	LY6K
hsa_circ_0043256	hsa_miR_576-5p	PCDH17
hsa_circ_0043256	hsa_miR_576-5p	MRAS
hsa_circ_0043256	hsa_miR_576-5p	NPR3
hsa_circ_0043256	hsa_miR_576-5p	SALL3
hsa_circ_0043256	hsa_miR_576-5p	BACE2
hsa_circ_0043256	hsa_miR_576-5p	ARHGAP18
hsa_circ_0043256	hsa_miR_576-5p	SNX31
hsa_circ_0043256	hsa_miR_576-5p	RORA
hsa_circ_0043256	hsa_miR_576-5p	MICU3
hsa_circ_0043256	hsa_miR_576-5p	IL2RA
hsa_circ_0043256	hsa_miR_576-5p	NEGR1
hsa_circ_0043256	hsa_miR_576-5p	PRTFDC1
hsa_circ_0043256	hsa_miR_576-5p	CLSTN2
hsa_circ_0043256	hsa_miR_576-5p	OLFM4
hsa_circ_0043256	hsa_miR_576-5p	CDKN2B
hsa_circ_0043256	hsa_miR_576-5p	RTKN2
hsa_circ_0043256	hsa_miR_576-5p	NUP62CL
hsa_circ_0043256	hsa_miR_576-5p	UNC45B
hsa_circ_0043256	hsa_miR_576-5p	HS3ST5
hsa_circ_0043256	hsa_miR_576-5p	KCTD8
hsa_circ_0043256	hsa_miR_576-5p	CYP26A1
hsa_circ_0043256	hsa_miR_576-5p	QKI
hsa_circ_0043256	hsa_miR_576-5p	MEFV
hsa_circ_0043256	hsa_miR_576-5p	ZBTB20
hsa_circ_0043256	hsa_miR_576-5p	CALCRL
hsa_circ_0043256	hsa_miR_576-5p	FRMD4B
hsa_circ_0043256	hsa_miR_576-5p	PHEX
hsa_circ_0043256	hsa_miR_576-5p	ADAMTS19
hsa_circ_0043256	hsa_miR_576-5p	C20orf194
hsa_circ_0043256	hsa_miR_576-5p	BHMT
hsa_circ_0043256	hsa_miR_576-5p	CHAC2
hsa_circ_0043256	hsa_miR_576-5p	SLC22A11
hsa_circ_0043256	hsa_miR_576-5p	TCF24
hsa_circ_0043256	hsa_miR_576-5p	SHISA3
hsa_circ_0043256	hsa_miR_576-5p	GUCY1A2
hsa_circ_0043256	hsa_miR_576-5p	PRKCH
hsa_circ_0043256	hsa_miR_576-5p	LCTL
hsa_circ_0043256	hsa_miR_576-5p	SELE
hsa_circ_0043256	hsa_miR_576-5p	ACKR2
hsa_circ_0043256	hsa_miR_576-5p	TAF1D
hsa_circ_0043256	hsa_miR_576-5p	ENDOU
hsa_circ_0043256	hsa_miR_576-5p	RASSF8
hsa_circ_0043256	hsa_miR_576-5p	CRY2
hsa_circ_0043256	hsa_miR_576-5p	KIF20B
hsa_circ_0043256	hsa_miR_576-5p	CYP19A1
hsa_circ_0043256	hsa_miR_576-5p	ZNF793
hsa_circ_0043256	hsa_miR_576-5p	DAB2IP
hsa_circ_0043256	hsa_miR_576-5p	C1orf141
hsa_circ_0043256	hsa_miR_576-5p	SLC6A11
hsa_circ_0043256	hsa_miR_576-5p	ABI3BP

hsa_circ_0043256	hsa_miR_576-5p	MTMR7
hsa_circ_0043256	hsa_miR_576-5p	PAPSS2
hsa_circ_0043256	hsa_miR_576-5p	SLFN13
hsa_circ_0043256	hsa_miR_576-5p	PTHLH
hsa_circ_0043256	hsa_miR_576-5p	MAGEB16
hsa_circ_0043256	hsa_miR_576-5p	EFCAB13
hsa_circ_0043256	hsa_miR_576-5p	PHACTR2
hsa_circ_0043256	hsa_miR_576-5p	CHL1
hsa_circ_0043256	hsa_miR_576-5p	CDH19
hsa_circ_0043256	hsa_miR_576-5p	PROS1
hsa_circ_0043256	hsa_miR_576-5p	DGKB
hsa_circ_0043256	hsa_miR_576-5p	LYPLA1
hsa_circ_0043256	hsa_miR_576-5p	SPRY4
hsa_circ_0043256	hsa_miR_576-5p	HRG
hsa_circ_0043256	hsa_miR_576-5p	SOX21
hsa_circ_0043256	hsa_miR_576-5p	THSD4
hsa_circ_0043256	hsa_miR_576-5p	LUZP4
hsa_circ_0043256	hsa_miR_576-5p	ANGPTL5
hsa_circ_0043256	hsa_miR_576-5p	A1CF
hsa_circ_0043256	hsa_miR_576-5p	ARG1
hsa_circ_0043256	hsa_miR_576-5p	LEPR
hsa_circ_0043256	hsa_miR_576-5p	SGK1
hsa_circ_0043256	hsa_miR_576-5p	SLITRK5
hsa_circ_0043256	hsa_miR_576-5p	PCSK2
hsa_circ_0043256	hsa_miR_576-5p	UTRN
hsa_circ_0043256	hsa_miR_576-5p	GJA5
hsa_circ_0043256	hsa_miR_576-5p	ST8SIA3
hsa_circ_0043256	hsa_miR_576-5p	DCX
hsa_circ_0043256	hsa_miR_576-5p	C20orf197
hsa_circ_0043256	hsa_miR_576-5p	LCOR
hsa_circ_0043256	hsa_miR_576-5p	TENM3
hsa_circ_0043256	hsa_miR_576-5p	NEUROD1
hsa_circ_0043256	hsa_miR_576-5p	FSHB
hsa_circ_0043256	hsa_miR_576-5p	CERS6
hsa_circ_0043256	hsa_miR_576-5p	RUNX1T1
hsa_circ_0043256	hsa_miR_576-5p	PRLR
hsa_circ_0043256	hsa_miR_576-5p	KCNJ6
hsa_circ_0043256	hsa_miR_576-5p	IGSF1
hsa_circ_0043256	hsa_miR_576-5p	SYT1
hsa_circ_0043256	hsa_miR_576-5p	CLVS2
hsa_circ_0043256	hsa_miR_576-5p	AFF3
hsa_circ_0043256	hsa_miR_576-5p	SCG3
hsa_circ_0043256	hsa_miR_576-5p	GFRA1
hsa_circ_0043256	hsa_miR_576-5p	UGT2B4
hsa_circ_0043256	hsa_miR_576-5p	GSG1L
hsa_circ_0043256	hsa_miR_576-5p	ETV1
hsa_circ_0043256	hsa_miR_576-5p	PAK3
hsa_circ_0043256	hsa_miR_576-5p	COL3A1
hsa_circ_0043256	hsa_miR_576-5p	RBM24
hsa_circ_0043256	hsa_miR_576-5p	ROBO2
hsa_circ_0043256	hsa_miR_576-5p	UGT2A1
hsa_circ_0043256	hsa_miR_576-5p	TPTE
hsa_circ_0043256	hsa_miR_576-5p	CACNB3
hsa_circ_0043256	hsa_miR_576-5p	GDA
hsa_circ_0043256	hsa_miR_576-5p	SCN4B
hsa_circ_0043256	hsa_miR_576-5p	PCNA
hsa_circ_0043256	hsa_miR_576-5p	MOB3B
hsa_circ_0043256	hsa_miR_576-5p	MYOZ3

hsa_circ_0043256	hsa_miR_576-5p	EPHA5
hsa_circ_0043256	hsa_miR_576-5p	PLOD2
hsa_circ_0043256	hsa_miR_576-5p	ELAVL4
hsa_circ_0043256	hsa_miR_576-5p	RIMBP2
hsa_circ_0043256	hsa_miR_576-5p	FOXF2
hsa_circ_0043256	hsa_miR_576-5p	SST
hsa_circ_0043256	hsa_miR_576-5p	EMP2
hsa_circ_0043256	hsa_miR_576-5p	CD36
hsa_circ_0043256	hsa_miR_576-5p	ATRNL1
hsa_circ_0043256	hsa_miR_576-5p	NKD1
hsa_circ_0043256	hsa_miR_576-5p	UGT2A3
hsa_circ_0043256	hsa_miR_576-5p	TFAP2A
hsa_circ_0043256	hsa_miR_576-5p	FOXE1
hsa_circ_0043256	hsa_miR_576-5p	DNAJC22
hsa_circ_0043256	hsa_miR_576-5p	FUT8
hsa_circ_0043256	hsa_miR_576-5p	CCDC85A
hsa_circ_0043256	hsa_miR_576-5p	SEMA3D
hsa_circ_0043256	hsa_miR_576-5p	ZNF492
hsa_circ_0043256	hsa_miR_576-5p	KLF6
hsa_circ_0043256	hsa_miR_576-5p	SOCS2
hsa_circ_0043256	hsa_miR_576-5p	CLDN12
hsa_circ_0043256	hsa_miR_576-5p	DPP10
hsa_circ_0043256	hsa_miR_576-5p	EMCN
hsa_circ_0043256	hsa_miR_576-5p	GMNC
hsa_circ_0043256	hsa_miR_576-5p	SLCO1A2
hsa_circ_0043256	hsa_miR_576-5p	TSPAN6
hsa_circ_0043256	hsa_miR_576-5p	TMPRSS11E
hsa_circ_0043256	hsa_miR_576-5p	TMEM45B
hsa_circ_0043256	hsa_miR_576-5p	STXBP5L
hsa_circ_0043256	hsa_miR_576-5p	TMEM47
hsa_circ_0043256	hsa_miR_576-5p	SIX4
hsa_circ_0043256	hsa_miR_576-5p	CKAP2
hsa_circ_0043256	hsa_miR_576-5p	TET1
hsa_circ_0043256	hsa_miR_576-5p	BEST3
hsa_circ_0043256	hsa_miR_576-5p	SLC7A2
hsa_circ_0043256	hsa_miR_576-5p	RSPO2
hsa_circ_0043256	hsa_miR_576-5p	RNF144B
hsa_circ_0043256	hsa_miR_576-5p	NT5E
hsa_circ_0043256	hsa_miR_576-5p	PRKCQ
hsa_circ_0043256	hsa_miR_576-5p	ARRDC4
hsa_circ_0043256	hsa_miR_576-5p	AGTR2
hsa_circ_0043256	hsa_miR_576-5p	OSBPL6
hsa_circ_0043256	hsa_miR_576-5p	SPRED3
hsa_circ_0043256	hsa_miR_576-5p	RXFP1
hsa_circ_0043256	hsa_miR_576-5p	ASAH2B
hsa_circ_0043256	hsa_miR_576-5p	RALYL
hsa_circ_0043256	hsa_miR_576-5p	MAPK4
hsa_circ_0043256	hsa_miR_576-5p	SLC9A7
hsa_circ_0043256	hsa_miR_576-5p	LIPA
hsa_circ_0043256	hsa_miR_576-5p	MDGA2
hsa_circ_0043256	hsa_miR_576-5p	CLIC5
hsa_circ_0043256	hsa_miR_576-5p	NTRK2
hsa_circ_0043256	hsa_miR_576-5p	TMEM182
hsa_circ_0043256	hsa_miR_576-5p	CCND2
hsa_circ_0043256	hsa_miR_576-5p	GEN1
hsa_circ_0043256	hsa_miR_576-5p	TRIM17
hsa_circ_0043256	hsa_miR_576-5p	SASS6
hsa_circ_0043256	hsa_miR_576-5p	SGMS2

hsa_circ_0043256	hsa_miR_576-5p	HNF4G
hsa_circ_0043256	hsa_miR_576-5p	TGFA
hsa_circ_0043256	hsa_miR_576-5p	UBASH3B
hsa_circ_0043256	hsa_miR_576-5p	MASP1
hsa_circ_0043256	hsa_miR_576-5p	BRIP1
hsa_circ_0043256	hsa_miR_576-5p	EIF4E3
hsa_circ_0043256	hsa_miR_576-5p	MORC1
hsa_circ_0043256	hsa_miR_576-5p	CCNE2
hsa_circ_0043256	hsa_miR_576-5p	GJA1
hsa_circ_0043256	hsa_miR_576-5p	CDH7
hsa_circ_0043256	hsa_miR_576-5p	TWIST1
hsa_circ_0043256	hsa_miR_576-5p	GJA3
hsa_circ_0043256	hsa_miR_576-5p	ZNF322
hsa_circ_0043256	hsa_miR_576-5p	SASH1
hsa_circ_0043256	hsa_miR_576-5p	MYCT1
hsa_circ_0043256	hsa_miR_576-5p	ARHGAP29
hsa_circ_0043256	hsa_miR_576-5p	STK39
hsa_circ_0043256	hsa_miR_576-5p	CNR1
hsa_circ_0043256	hsa_miR_576-5p	PCDH9
hsa_circ_0043256	hsa_miR_576-5p	TMEM215
hsa_circ_0043256	hsa_miR_576-5p	EPHA7
hsa_circ_0043256	hsa_miR_576-5p	DMC1
hsa_circ_0043256	hsa_miR_576-5p	CPED1
hsa_circ_0043256	hsa_miR_576-5p	RNF157
hsa_circ_0043256	hsa_miR_576-5p	IL7R
hsa_circ_0043256	hsa_miR_330-3p	GNRHR
hsa_circ_0043256	hsa_miR_330-3p	RCAN1
hsa_circ_0043256	hsa_miR_330-3p	PTGFR
hsa_circ_0043256	hsa_miR_330-3p	SOSTDC1
hsa_circ_0043256	hsa_miR_330-3p	TAC3
hsa_circ_0043256	hsa_miR_330-3p	RSPO2
hsa_circ_0043256	hsa_miR_330-3p	KLF10
hsa_circ_0043256	hsa_miR_330-3p	ADAM12
hsa_circ_0043256	hsa_miR_330-3p	CLDN18
hsa_circ_0043256	hsa_miR_330-3p	DLX1
hsa_circ_0043256	hsa_miR_330-3p	UBL3
hsa_circ_0043256	hsa_miR_330-3p	ZNF367
hsa_circ_0043256	hsa_miR_330-3p	RAI2
hsa_circ_0043256	hsa_miR_330-3p	AGTR2
hsa_circ_0043256	hsa_miR_330-3p	GPR37
hsa_circ_0043256	hsa_miR_330-3p	ZNF423
hsa_circ_0043256	hsa_miR_330-3p	PRTFDC1
hsa_circ_0043256	hsa_miR_330-3p	TXNDC17
hsa_circ_0043256	hsa_miR_330-3p	DLX6
hsa_circ_0043256	hsa_miR_330-3p	S100B
hsa_circ_0043256	hsa_miR_330-3p	C1orf115
hsa_circ_0043256	hsa_miR_330-3p	ITM2C
hsa_circ_0043256	hsa_miR_330-3p	TDRKH
hsa_circ_0043256	hsa_miR_330-3p	USP2
hsa_circ_0043256	hsa_miR_330-3p	ALAS2
hsa_circ_0043256	hsa_miR_330-3p	KLHL32
hsa_circ_0043256	hsa_miR_330-3p	NDC1
hsa_circ_0043256	hsa_miR_330-3p	MYPN
hsa_circ_0043256	hsa_miR_330-3p	SHOX2
hsa_circ_0043256	hsa_miR_330-3p	CDK1
hsa_circ_0043256	hsa_miR_330-3p	KCNC2
hsa_circ_0043256	hsa_miR_330-3p	S1PR1
hsa_circ_0043256	hsa_miR_330-3p	ONECUT2

hsa_circ_0043256	hsa_miR_330-3p	ELAVL2
hsa_circ_0043256	hsa_miR_330-3p	TBX5
hsa_circ_0043256	hsa_miR_330-3p	DTL
hsa_circ_0043256	hsa_miR_330-3p	SCG3
hsa_circ_0043256	hsa_miR_330-3p	SALL4
hsa_circ_0043256	hsa_miR_330-3p	SPRYD7
hsa_circ_0043256	hsa_miR_330-3p	FRAS1
hsa_circ_0043256	hsa_miR_330-3p	PGAM5
hsa_circ_0043256	hsa_miR_330-3p	TFAP2B
hsa_circ_0043256	hsa_miR_330-3p	SMAD7
hsa_circ_0043256	hsa_miR_330-3p	MGAM
hsa_circ_0043256	hsa_miR_330-3p	TPBG
hsa_circ_0043256	hsa_miR_330-3p	ARHGEF10
hsa_circ_0043256	hsa_miR_330-3p	JAM2
hsa_circ_0043256	hsa_miR_330-3p	SLC24A2
hsa_circ_0043256	hsa_miR_330-3p	MYRF
hsa_circ_0043256	hsa_miR_330-3p	ERBB4
hsa_circ_0043256	hsa_miR_330-3p	LAPTM5
hsa_circ_0043256	hsa_miR_330-3p	MICU3
hsa_circ_0043256	hsa_miR_330-3p	GBX2
hsa_circ_0043256	hsa_miR_330-3p	GDF6
hsa_circ_0043256	hsa_miR_330-3p	HOXC8
hsa_circ_0043256	hsa_miR_330-3p	SLC28A3
hsa_circ_0043256	hsa_miR_330-3p	GALNT7
hsa_circ_0043256	hsa_miR_330-3p	PROX1
hsa_circ_0043256	hsa_miR_330-3p	TSHR
hsa_circ_0043256	hsa_miR_330-3p	PPARGC1B
hsa_circ_0043256	hsa_miR_330-3p	COL6A5
hsa_circ_0043256	hsa_miR_330-3p	PTPRM
hsa_circ_0043256	hsa_miR_330-3p	RFWD3
hsa_circ_0043256	hsa_miR_330-3p	BFSP2
hsa_circ_0043256	hsa_miR_330-3p	DIRAS2
hsa_circ_0043256	hsa_miR_330-3p	KAT2B
hsa_circ_0043256	hsa_miR_330-3p	EGR4
hsa_circ_0043256	hsa_miR_330-3p	DRP2
hsa_circ_0043256	hsa_miR_330-3p	STXBP5L
hsa_circ_0043256	hsa_miR_330-3p	ST8SIA3
hsa_circ_0043256	hsa_miR_330-3p	KANK2
hsa_circ_0043256	hsa_miR_330-3p	NRG3
hsa_circ_0043256	hsa_miR_330-3p	LRP8
hsa_circ_0043256	hsa_miR_330-3p	ADCY9
hsa_circ_0043256	hsa_miR_330-3p	L1CAM
hsa_circ_0043256	hsa_miR_330-3p	C1orf21
hsa_circ_0043256	hsa_miR_330-3p	SEMA3A
hsa_circ_0043256	hsa_miR_330-3p	TGFBR3
hsa_circ_0043256	hsa_miR_330-3p	E2F1
hsa_circ_0043256	hsa_miR_330-3p	TNFAIP3
hsa_circ_0043256	hsa_miR_330-3p	NDNF
hsa_circ_0043256	hsa_miR_330-3p	SCN1A
hsa_circ_0043256	hsa_miR_330-3p	PCYT1B
hsa_circ_0043256	hsa_miR_330-3p	NAT8L
hsa_circ_0043256	hsa_miR_330-3p	PRKG1
hsa_circ_0043256	hsa_miR_330-3p	BMPR2
hsa_circ_0043256	hsa_miR_330-3p	NKAIN1
hsa_circ_0043256	hsa_miR_330-3p	ADCYAP1R1
hsa_circ_0043256	hsa_miR_330-3p	SYT5
hsa_circ_0043256	hsa_miR_330-3p	GJA3
hsa_circ_0043256	hsa_miR_330-3p	ADRA2A

hsa_circ_0043256	hsa_miR_330-3p	ATP2B2
hsa_circ_0043256	hsa_miR_330-3p	MYH10
hsa_circ_0043256	hsa_miR_330-3p	NPR3
hsa_circ_0043256	hsa_miR_330-3p	ANKRD33B
hsa_circ_0043256	hsa_miR_330-3p	FBLN5
hsa_circ_0043256	hsa_miR_330-3p	MAPK10
hsa_circ_0043256	hsa_miR_330-3p	JPH3
hsa_circ_0043256	hsa_miR_330-3p	CA12
hsa_circ_0043256	hsa_miR_330-3p	GPM6A
hsa_circ_0043256	hsa_miR_330-3p	PPM1H
hsa_circ_0043256	hsa_miR_330-3p	TYRP1
hsa_circ_0043256	hsa_miR_330-3p	PCDHA7
hsa_circ_0043256	hsa_miR_330-3p	PCDHA12
hsa_circ_0043256	hsa_miR_330-3p	PCDHA5
hsa_circ_0043256	hsa_miR_330-3p	PCDHA11
hsa_circ_0043256	hsa_miR_330-3p	PCDHA9
hsa_circ_0043256	hsa_miR_330-3p	PCDHA6
hsa_circ_0043256	hsa_miR_330-3p	PCDHA1
hsa_circ_0043256	hsa_miR_330-3p	PCDHA4
hsa_circ_0043256	hsa_miR_330-3p	ZBTB20
hsa_circ_0043256	hsa_miR_433	GCLC
hsa_circ_0043256	hsa_miR_433	FERMT2
hsa_circ_0043256	hsa_miR_433	B4GALT3
hsa_circ_0043256	hsa_miR_433	IGFBP1
hsa_circ_0043256	hsa_miR_433	GPC5
hsa_circ_0043256	hsa_miR_433	NR2F6
hsa_circ_0043256	hsa_miR_433	C1orf131
hsa_circ_0043256	hsa_miR_433	HOXA1
hsa_circ_0043256	hsa_miR_433	TLR10
hsa_circ_0043256	hsa_miR_433	SASS6
hsa_circ_0043256	hsa_miR_433	ST6GALNAC3
hsa_circ_0043256	hsa_miR_433	EPHA7
hsa_circ_0043256	hsa_miR_433	SGMS2
hsa_circ_0043256	hsa_miR_433	TDRD5
hsa_circ_0043256	hsa_miR_433	E2F3
hsa_circ_0043256	hsa_miR_433	CHL1
hsa_circ_0043256	hsa_miR_433	HECW2
hsa_circ_0043256	hsa_miR_433	QKI
hsa_circ_0043256	hsa_miR_433	TENM1
hsa_circ_0043256	hsa_miR_668	ID4
hsa_circ_0043256	hsa_miR_668	TOX3
hsa_circ_0043256	hsa_miR_668	AK4
hsa_circ_0043256	hsa_miR_668	LMOD3
hsa_circ_0043256	hsa_miR_668	NEGR1
hsa_circ_0043256	hsa_miR_668	SCN2B
hsa_circ_0043256	hsa_miR_668	C1orf189
hsa_circ_0043256	hsa_miR_668	FUT8
hsa_circ_0043256	hsa_miR_668	CDH2
hsa_circ_0049271	hsa_miR_1206	SNTG1
hsa_circ_0049271	hsa_miR_1206	TMEFF1
hsa_circ_0049271	hsa_miR_1206	KCND2
hsa_circ_0049271	hsa_miR_1206	SEMA6A
hsa_circ_0049271	hsa_miR_1206	KCNQ5
hsa_circ_0049271	hsa_miR_1206	HOXA1
hsa_circ_0049271	hsa_miR_1206	LRP1B
hsa_circ_0049271	hsa_miR_1206	NDNF
hsa_circ_0049271	hsa_miR_1206	FUT8
hsa_circ_0049271	hsa_miR_1206	NR5A2

hsa_circ_0049271	hsa_miR_1206	DSCC1
hsa_circ_0049271	hsa_miR_1206	DOK6
hsa_circ_0049271	hsa_miR_1206	SKA3
hsa_circ_0049271	hsa_miR_1206	THRB
hsa_circ_0049271	hsa_miR_1206	KLHL32
hsa_circ_0049271	hsa_miR_1206	LRRC31
hsa_circ_0049271	hsa_miR_1206	KCNB2
hsa_circ_0049271	hsa_miR_1206	TIMP3
hsa_circ_0049271	hsa_miR_1206	SHISA3
hsa_circ_0049271	hsa_miR_1206	TPBG
hsa_circ_0049271	hsa_miR_1206	VSTM4
hsa_circ_0049271	hsa_miR_1206	GRIK2
hsa_circ_0049271	hsa_miR_1206	FAT1
hsa_circ_0049271	hsa_miR_1206	HTR4
hsa_circ_0049271	hsa_miR_1206	RHNO1
hsa_circ_0049271	hsa_miR_1206	PHEX
hsa_circ_0049271	hsa_miR_1206	NTN4
hsa_circ_0049271	hsa_miR_1206	TLL2
hsa_circ_0049271	hsa_miR_1206	QKI
hsa_circ_0049271	hsa_miR_1206	NIPSNAP1
hsa_circ_0049271	hsa_miR_1206	OPRK1
hsa_circ_0049271	hsa_miR_1206	GCLM
hsa_circ_0049271	hsa_miR_1206	ADAMTS19
hsa_circ_0049271	hsa_miR_1206	IGF2
hsa_circ_0049271	hsa_miR_1206	PROS1
hsa_circ_0049271	hsa_miR_1206	FAXC
hsa_circ_0049271	hsa_miR_1206	CCDC141
hsa_circ_0049271	hsa_miR_1206	PHF21B
hsa_circ_0049271	hsa_miR_1206	ABCA10
hsa_circ_0049271	hsa_miR_1206	SASH1
hsa_circ_0049271	hsa_miR_1206	SORBS1
hsa_circ_0049271	hsa_miR_1206	EPHA7
hsa_circ_0049271	hsa_miR_1206	TIMM8A
hsa_circ_0049271	hsa_miR_1206	ZBTB20
hsa_circ_0049271	hsa_miR_1206	MRAS
hsa_circ_0049271	hsa_miR_1206	SLC27A2
hsa_circ_0049271	hsa_miR_1206	FUT9
hsa_circ_0049271	hsa_miR_1206	FAM216B
hsa_circ_0049271	hsa_miR_1206	CCBE1
hsa_circ_0049271	hsa_miR_1206	CHML
hsa_circ_0049271	hsa_miR_1206	GDAP1L1
hsa_circ_0049271	hsa_miR_1206	F2RL2
hsa_circ_0049271	hsa_miR_1206	ALDH1L2
hsa_circ_0049271	hsa_miR_1206	BARD1
hsa_circ_0049271	hsa_miR_1206	NKAIN3
hsa_circ_0049271	hsa_miR_1206	ZNF367
hsa_circ_0049271	hsa_miR_1206	RIPPLY3
hsa_circ_0049271	hsa_miR_1206	RBMS3
hsa_circ_0049271	hsa_miR_1206	SHROOM4
hsa_circ_0049271	hsa_miR_1206	CAMK2B
hsa_circ_0049271	hsa_miR_1206	C1QTNF7
hsa_circ_0049271	hsa_miR_1206	SSTR1
hsa_circ_0049271	hsa_miR_1206	NTM
hsa_circ_0049271	hsa_miR_1206	EGLN3
hsa_circ_0049271	hsa_miR_1206	SYTL5
hsa_circ_0049271	hsa_miR_1206	DUSP4
hsa_circ_0049271	hsa_miR_1206	SCN3A
hsa_circ_0049271	hsa_miR_1206	ROR1

hsa_circ_0049271	hsa_miR_1206	SEMA3A
hsa_circ_0049271	hsa_miR_1206	RASSF6
hsa_circ_0049271	hsa_miR_1206	KCNB1
hsa_circ_0049271	hsa_miR_1206	PEAR1
hsa_circ_0049271	hsa_miR_1206	CDH10
hsa_circ_0049271	hsa_miR_1206	GUCY1A2
hsa_circ_0049271	hsa_miR_1206	MITF
hsa_circ_0049271	hsa_miR_1206	NOVA1
hsa_circ_0049271	hsa_miR_1206	RS1
hsa_circ_0049271	hsa_miR_1206	SLC5A7
hsa_circ_0049271	hsa_miR_1206	KCNK15
hsa_circ_0049271	hsa_miR_1206	CBR1
hsa_circ_0049271	hsa_miR_1206	VSX2
hsa_circ_0049271	hsa_miR_1206	PRDM5
hsa_circ_0049271	hsa_miR_1206	FRMD3
hsa_circ_0049271	hsa_miR_1206	TFF3
hsa_circ_0049271	hsa_miR_1206	KCNJ1
hsa_circ_0049271	hsa_miR_1206	RASSF8
hsa_circ_0049271	hsa_miR_1206	ROBO2
hsa_circ_0049271	hsa_miR_1206	RASSF2
hsa_circ_0049271	hsa_miR_1206	SLC2A2
hsa_circ_0049271	hsa_miR_1206	RD3
hsa_circ_0049271	hsa_miR_1206	IL5RA
hsa_circ_0049271	hsa_miR_1206	SCAI
hsa_circ_0049271	hsa_miR_1206	CREG2
hsa_circ_0049271	hsa_miR_1206	NSG2
hsa_circ_0049271	hsa_miR_1206	TMEM132C
hsa_circ_0049271	hsa_miR_1206	CHL1
hsa_circ_0049271	hsa_miR_1206	C20orf197
hsa_circ_0049271	hsa_miR_1256	CDKN2B
hsa_circ_0049271	hsa_miR_1256	SLC23A3
hsa_circ_0049271	hsa_miR_1256	ZNF474
hsa_circ_0049271	hsa_miR_1256	PDZRN4
hsa_circ_0049271	hsa_miR_1256	PFN2
hsa_circ_0049271	hsa_miR_1256	FUT8
hsa_circ_0049271	hsa_miR_1256	GABRE
hsa_circ_0049271	hsa_miR_1256	N4BP2L1
hsa_circ_0049271	hsa_miR_1256	STX11
hsa_circ_0049271	hsa_miR_1256	NR2E1
hsa_circ_0049271	hsa_miR_1256	HTR3A
hsa_circ_0049271	hsa_miR_1256	LRRC18
hsa_circ_0049271	hsa_miR_1256	CYYR1
hsa_circ_0049271	hsa_miR_1256	C12orf42
hsa_circ_0049271	hsa_miR_1256	GALNT4
hsa_circ_0049271	hsa_miR_1256	ZHX1-C8orf76
hsa_circ_0049271	hsa_miR_1256	PCDH10
hsa_circ_0049271	hsa_miR_1256	IGF2BP1
hsa_circ_0049271	hsa_miR_1256	SCN1A
hsa_circ_0049271	hsa_miR_1256	LCOR
hsa_circ_0049271	hsa_miR_1256	PCSK2
hsa_circ_0049271	hsa_miR_1256	UNC5D
hsa_circ_0049271	hsa_miR_1256	TMEM196
hsa_circ_0049271	hsa_miR_1256	DPP10
hsa_circ_0049271	hsa_miR_1256	CT62
hsa_circ_0049271	hsa_miR_1256	COL11A1
hsa_circ_0049271	hsa_miR_1256	MYPN
hsa_circ_0049271	hsa_miR_1256	ZNF107
hsa_circ_0049271	hsa_miR_1256	SLC24A2

hsa_circ_0049271	hsa_miR_1256	FRMD3
hsa_circ_0049271	hsa_miR_1256	BCHE
hsa_circ_0049271	hsa_miR_1256	PUS7
hsa_circ_0049271	hsa_miR_1256	ANKRD29
hsa_circ_0049271	hsa_miR_1256	KANK4
hsa_circ_0049271	hsa_miR_1256	RDH10
hsa_circ_0049271	hsa_miR_1256	NDST1
hsa_circ_0049271	hsa_miR_1256	SLC16A14
hsa_circ_0049271	hsa_miR_1256	ST6GALNAC5
hsa_circ_0049271	hsa_miR_1256	ASGR1
hsa_circ_0049271	hsa_miR_1256	ST8SIA3
hsa_circ_0049271	hsa_miR_1256	NTRK2
hsa_circ_0049271	hsa_miR_1256	OTUD1
hsa_circ_0049271	hsa_miR_1256	PHACTR2
hsa_circ_0049271	hsa_miR_1256	GRIN2A
hsa_circ_0049271	hsa_miR_1256	TRIM9
hsa_circ_0049271	hsa_miR_1256	CERS6
hsa_circ_0049271	hsa_miR_1256	EYA4
hsa_circ_0049271	hsa_miR_1256	ZIC5
hsa_circ_0049271	hsa_miR_1256	C2orf72
hsa_circ_0049271	hsa_miR_1256	PDE11A
hsa_circ_0049271	hsa_miR_1256	CILP
hsa_circ_0049271	hsa_miR_1256	GTSF1
hsa_circ_0049271	hsa_miR_1256	PRLR
hsa_circ_0049271	hsa_miR_1256	CFL2
hsa_circ_0049271	hsa_miR_1256	FOXG1
hsa_circ_0049271	hsa_miR_1256	FHL5
hsa_circ_0049271	hsa_miR_1256	GABBR2
hsa_circ_0049271	hsa_miR_1256	TRIM29
hsa_circ_0049271	hsa_miR_1256	EFNA5
hsa_circ_0049271	hsa_miR_1256	RCC2
hsa_circ_0049271	hsa_miR_1256	P2RX5
hsa_circ_0049271	hsa_miR_1256	FAM124A
hsa_circ_0049271	hsa_miR_1256	CHST15
hsa_circ_0049271	hsa_miR_1256	LIN28B
hsa_circ_0049271	hsa_miR_1256	LRRC55
hsa_circ_0049271	hsa_miR_1256	TGFA
hsa_circ_0049271	hsa_miR_1256	ADAM12
hsa_circ_0049271	hsa_miR_1256	KRT75
hsa_circ_0049271	hsa_miR_1256	CNGB1
hsa_circ_0049271	hsa_miR_1256	EPB41L2
hsa_circ_0049271	hsa_miR_1256	NEUROD1
hsa_circ_0049271	hsa_miR_1256	TTC28
hsa_circ_0049271	hsa_miR_1256	DSG4
hsa_circ_0049271	hsa_miR_1256	PREX2
hsa_circ_0049271	hsa_miR_1256	CEACAM6
hsa_circ_0049271	hsa_miR_1256	XAGE1B
hsa_circ_0049271	hsa_miR_1256	TMOD1
hsa_circ_0049271	hsa_miR_1256	AQP4
hsa_circ_0049271	hsa_miR_1256	KIAA1549L
hsa_circ_0049271	hsa_miR_1256	BASP1
hsa_circ_0049271	hsa_miR_1256	SCML2
hsa_circ_0049271	hsa_miR_1256	FAM111B
hsa_circ_0049271	hsa_miR_1256	VSTM4
hsa_circ_0049271	hsa_miR_1256	SEMA6A
hsa_circ_0049271	hsa_miR_1256	GPX8
hsa_circ_0049271	hsa_miR_1256	DCC
hsa_circ_0049271	hsa_miR_1256	ZNF793

hsa_circ_0049271	hsa_miR_1256	HSD3B2
hsa_circ_0049271	hsa_miR_1256	CYBB
hsa_circ_0049271	hsa_miR_1256	GREB1
hsa_circ_0049271	hsa_miR_1256	NBPF4
hsa_circ_0049271	hsa_miR_1256	CCDC177
hsa_circ_0049271	hsa_miR_1256	DMRT3
hsa_circ_0049271	hsa_miR_1256	RGS7
hsa_circ_0049271	hsa_miR_1256	PRSS35
hsa_circ_0049271	hsa_miR_1256	LGSN
hsa_circ_0049271	hsa_miR_1256	SLCO2A1
hsa_circ_0049271	hsa_miR_1256	TFAP2B
hsa_circ_0049271	hsa_miR_1256	HTR4
hsa_circ_0049271	hsa_miR_1256	RAB11FIP1
hsa_circ_0049271	hsa_miR_1256	SPTSSB
hsa_circ_0049271	hsa_miR_1256	CLEC1A
hsa_circ_0049271	hsa_miR_1256	CYP26A1
hsa_circ_0049271	hsa_miR_604	IGSF10
hsa_circ_0049271	hsa_miR_604	PLCH2
hsa_circ_0049271	hsa_miR_604	SCN8A
hsa_circ_0049271	hsa_miR_604	KRTCAP3
hsa_circ_0049271	hsa_miR_604	SLC6A19
hsa_circ_0049271	hsa_miR_604	PPP1R14D
hsa_circ_0049271	hsa_miR_604	TMEM212
hsa_circ_0049271	hsa_miR_604	GDNF
hsa_circ_0049271	hsa_miR_604	SCN2B
hsa_circ_0049271	hsa_miR_604	DOK7
hsa_circ_0049271	hsa_miR_604	MYBL1
hsa_circ_0049271	hsa_miR_604	SLC11A1
hsa_circ_0049271	hsa_miR_604	PAK6
hsa_circ_0049271	hsa_miR_604	KCNB1
hsa_circ_0049271	hsa_miR_604	PPM1F
hsa_circ_0049271	hsa_miR_604	CA10
hsa_circ_0049271	hsa_miR_604	MOV10L1
hsa_circ_0049271	hsa_miR_604	GALNT16
hsa_circ_0049271	hsa_miR_604	NTSR2
hsa_circ_0049271	hsa_miR_604	WNT8B
hsa_circ_0049271	hsa_miR_604	CIT
hsa_circ_0049271	hsa_miR_604	TMEM139
hsa_circ_0049271	hsa_miR_604	C12orf42
hsa_circ_0049271	hsa_miR_604	GRIA2
hsa_circ_0049271	hsa_miR_604	APBA2
hsa_circ_0049271	hsa_miR_604	CABLES2
hsa_circ_0049271	hsa_miR_604	SLC24A4
hsa_circ_0049271	hsa_miR_604	LINGO2
hsa_circ_0049271	hsa_miR_604	ARHGEF37
hsa_circ_0049271	hsa_miR_604	ABHD6
hsa_circ_0049271	hsa_miR_604	REEP6
hsa_circ_0049271	hsa_miR_604	ARHGEF15
hsa_circ_0049271	hsa_miR_604	RASSF2
hsa_circ_0049271	hsa_miR_604	ESPN
hsa_circ_0049271	hsa_miR_604	LHX6
hsa_circ_0049271	hsa_miR_604	DUSP8
hsa_circ_0049271	hsa_miR_604	NKX2-5
hsa_circ_0049271	hsa_miR_604	HNF1A
hsa_circ_0049271	hsa_miR_604	GRIK3
hsa_circ_0049271	hsa_miR_604	EFNA5
hsa_circ_0049271	hsa_miR_433	GCLC
hsa_circ_0049271	hsa_miR_433	FERMT2

hsa_circ_0049271	hsa_miR_433	B4GALT3
hsa_circ_0049271	hsa_miR_433	IGFBP1
hsa_circ_0049271	hsa_miR_433	GPC5
hsa_circ_0049271	hsa_miR_433	NR2F6
hsa_circ_0049271	hsa_miR_433	C1orf131
hsa_circ_0049271	hsa_miR_433	HOXA1
hsa_circ_0049271	hsa_miR_433	TLR10
hsa_circ_0049271	hsa_miR_433	SASS6
hsa_circ_0049271	hsa_miR_433	ST6GALNAC3
hsa_circ_0049271	hsa_miR_433	EPHA7
hsa_circ_0049271	hsa_miR_433	SGMS2
hsa_circ_0049271	hsa_miR_433	TDRD5
hsa_circ_0049271	hsa_miR_433	E2F3
hsa_circ_0049271	hsa_miR_433	CHL1
hsa_circ_0049271	hsa_miR_433	HECW2
hsa_circ_0049271	hsa_miR_433	QKI
hsa_circ_0049271	hsa_miR_433	TENM1
hsa_circ_0049271	hsa_miR_486-3p	GPX8
hsa_circ_0049271	hsa_miR_486-3p	C5orf64
hsa_circ_0049271	hsa_miR_486-3p	SP5
hsa_circ_0049271	hsa_miR_486-3p	COL6A6
hsa_circ_0049271	hsa_miR_486-3p	CDH7
hsa_circ_0049271	hsa_miR_486-3p	ST6GALNAC6
hsa_circ_0049271	hsa_miR_486-3p	PIK3R1
hsa_circ_0049271	hsa_miR_486-3p	SMOC1
hsa_circ_0049271	hsa_miR_486-3p	AFF3
hsa_circ_0049271	hsa_miR_486-3p	SEMA3A
hsa_circ_0049271	hsa_miR_486-3p	DOCK3
hsa_circ_0049271	hsa_miR_486-3p	CNKSR2
hsa_circ_0049271	hsa_miR_486-3p	CDK4
hsa_circ_0049271	hsa_miR_486-3p	CELF2
hsa_circ_0049271	hsa_miR_486-3p	ARHGAP44
hsa_circ_0049271	hsa_miR_486-3p	NCKAP5
hsa_circ_0049271	hsa_miR_486-3p	CLDN10
hsa_circ_0049271	hsa_miR_486-3p	KCNQ3
hsa_circ_0049271	hsa_miR_486-3p	DKK2
hsa_circ_0049271	hsa_miR_486-3p	KDM5B
hsa_circ_0049271	hsa_miR_486-3p	FAT3
hsa_circ_0049271	hsa_miR_486-3p	TMEM178B
hsa_circ_0049271	hsa_miR_486-3p	TBX2
hsa_circ_0049271	hsa_miR_486-3p	CELSR3
hsa_circ_0049271	hsa_miR_486-3p	CADM1
hsa_circ_0049271	hsa_miR_767-3p	PTPRT
hsa_circ_0049271	hsa_miR_767-3p	JPH3
hsa_circ_0049271	hsa_miR_767-3p	CNR1
hsa_circ_0049271	hsa_miR_767-3p	FHL1
hsa_circ_0049271	hsa_miR_767-3p	ARAP2
hsa_circ_0049271	hsa_miR_767-3p	GJB2
hsa_circ_0049271	hsa_miR_767-3p	SIRPB1
hsa_circ_0049271	hsa_miR_767-3p	DONSON
hsa_circ_0049271	hsa_miR_767-3p	NEFH
hsa_circ_0049271	hsa_miR_767-3p	KDELR3
hsa_circ_0049271	hsa_miR_767-3p	PCDHA5
hsa_circ_0049271	hsa_miR_767-3p	PCDHA6
hsa_circ_0049271	hsa_miR_767-3p	PCDHA11
hsa_circ_0049271	hsa_miR_767-3p	ADCYAP1R1
hsa_circ_0049271	hsa_miR_767-3p	PCDHA4
hsa_circ_0049271	hsa_miR_767-3p	PCDHA1

hsa_circ_0049271	hsa_miR_767-3p	PRICKLE1
hsa_circ_0049271	hsa_miR_767-3p	PCDHA7
hsa_circ_0049271	hsa_miR_767-3p	PCDHA12
hsa_circ_0049271	hsa_miR_767-3p	VSTM2L
hsa_circ_0049271	hsa_miR_767-3p	C20orf194
hsa_circ_0049271	hsa_miR_767-3p	LRRC32
hsa_circ_0049271	hsa_miR_767-3p	LATS2
hsa_circ_0049271	hsa_miR_767-3p	CHRD
hsa_circ_0049271	hsa_miR_767-3p	CLDN18
hsa_circ_0049271	hsa_miR_767-3p	ZNF710
hsa_circ_0049271	hsa_miR_767-3p	NFASC
hsa_circ_0049271	hsa_miR_767-3p	P2RX2
hsa_circ_0049271	hsa_miR_767-3p	ZEB2
hsa_circ_0049271	hsa_miR_767-3p	PCDHA9
hsa_circ_0049271	hsa_miR_767-3p	CHAC1
hsa_circ_0049271	hsa_miR_767-3p	CPLX1
hsa_circ_0049271	hsa_miR_767-3p	FAM83A
hsa_circ_0049271	hsa_miR_767-3p	TMPRSS15
hsa_circ_0049271	hsa_miR_767-3p	BTG2
hsa_circ_0049271	hsa_miR_767-3p	MTHFD2
hsa_circ_0049271	hsa_miR_767-3p	ZBTB20
hsa_circ_0049271	hsa_miR_767-3p	PCYOX1
hsa_circ_0049271	hsa_miR_767-3p	WWC2
hsa_circ_0049271	hsa_miR_767-3p	APOBEC4
hsa_circ_0049271	hsa_miR_767-3p	KIF4A
hsa_circ_0049271	hsa_miR_767-3p	UCK2
hsa_circ_0049271	hsa_miR_767-3p	TRIM71
hsa_circ_0049271	hsa_miR_767-3p	ANKRD22
hsa_circ_0049271	hsa_miR_767-3p	KL
hsa_circ_0049271	hsa_miR_767-3p	KLF6
hsa_circ_0049271	hsa_miR_767-3p	RIMS4
hsa_circ_0049271	hsa_miR_767-3p	NRG3
hsa_circ_0049271	hsa_miR_767-3p	FRMD5
hsa_circ_0049271	hsa_miR_767-3p	PPP4R4
hsa_circ_0049271	hsa_miR_767-3p	FHDC1
hsa_circ_0049271	hsa_miR_767-3p	CARD17
hsa_circ_0049271	hsa_miR_767-3p	SCN8A
hsa_circ_0049271	hsa_miR_767-3p	PPFIA4
hsa_circ_0049271	hsa_miR_767-3p	PRCD
hsa_circ_0049271	hsa_miR_767-3p	ARNTL2
hsa_circ_0049271	hsa_miR_767-3p	LCOR
hsa_circ_0049271	hsa_miR_767-3p	LIN28B
hsa_circ_0049271	hsa_miR_767-3p	ELOVL6
hsa_circ_0049271	hsa_miR_767-3p	PCDHA8
hsa_circ_0049271	hsa_miR_767-3p	RAB3B
hsa_circ_0049271	hsa_miR_767-3p	OTUB2
hsa_circ_0049271	hsa_miR_767-3p	PFKP
hsa_circ_0049271	hsa_miR_767-3p	GRIK3
hsa_circ_0049271	hsa_miR_767-3p	CD300E
hsa_circ_0049271	hsa_miR_767-3p	DCN
hsa_circ_0049271	hsa_miR_767-3p	ONECUT2
hsa_circ_0049271	hsa_miR_767-3p	NDST1
hsa_circ_0049271	hsa_miR_767-3p	STON1
hsa_circ_0049271	hsa_miR_767-3p	ECM1
hsa_circ_0049271	hsa_miR_767-3p	GAGE1
hsa_circ_0049271	hsa_miR_767-3p	KIF14
hsa_circ_0049271	hsa_miR_767-3p	SNCA
hsa_circ_0049271	hsa_miR_767-3p	WNT3

hsa_circ_0049271	hsa_miR_767-3p	PPIL1
hsa_circ_0049271	hsa_miR_767-3p	MARCO
hsa_circ_0049271	hsa_miR_767-3p	RUNX2
hsa_circ_0049271	hsa_miR_767-3p	PPARGC1B
hsa_circ_0049271	hsa_miR_767-3p	SEC14L4
hsa_circ_0049271	hsa_miR_767-3p	ANKRD33B
hsa_circ_0049271	hsa_miR_767-3p	AMER2
hsa_circ_0049271	hsa_miR_767-3p	LSAMP
hsa_circ_0049271	hsa_miR_767-3p	SYT1
hsa_circ_0049271	hsa_miR_767-3p	PDE2A
hsa_circ_0049271	hsa_miR_767-3p	TUBAL3
hsa_circ_0049271	hsa_miR_767-3p	XPO5
hsa_circ_0049271	hsa_miR_767-3p	ARRB1
hsa_circ_0049271	hsa_miR_767-3p	SOX4
hsa_circ_0049271	hsa_miR_767-3p	KAZN
hsa_circ_0049271	hsa_miR_767-3p	SNRK
hsa_circ_0049271	hsa_miR_767-3p	BRSK2
hsa_circ_0049271	hsa_miR_767-3p	DUOX1
hsa_circ_0049271	hsa_miR_767-3p	PPM1H
hsa_circ_0049271	hsa_miR_767-3p	MYOZ1
hsa_circ_0049271	hsa_miR_767-3p	ADARB1
hsa_circ_0049271	hsa_miR_767-3p	CCDC78
hsa_circ_0049271	hsa_miR_767-3p	ASPH
hsa_circ_0049271	hsa_miR_767-3p	BMX
hsa_circ_0049271	hsa_miR_767-3p	DCDC2B
hsa_circ_0049271	hsa_miR_767-3p	PLEKHA8
hsa_circ_0049271	hsa_miR_767-3p	PTPRQ
hsa_circ_0049271	hsa_miR_767-3p	IGLON5
hsa_circ_0049271	hsa_miR_767-3p	PAPPA
hsa_circ_0049271	hsa_miR_767-3p	CAV2
hsa_circ_0049271	hsa_miR_767-3p	ATP2B2
hsa_circ_0049271	hsa_miR_767-3p	DDN
hsa_circ_0049271	hsa_miR_767-3p	SLC1A4
hsa_circ_0049271	hsa_miR_767-3p	PCK1
hsa_circ_0049271	hsa_miR_767-3p	SLITRK1
hsa_circ_0049271	hsa_miR_767-3p	DIXDC1
hsa_circ_0049271	hsa_miR_767-3p	SIM1
hsa_circ_0049271	hsa_miR_767-3p	KIF4B
hsa_circ_0049271	hsa_miR_767-3p	DUSP8
hsa_circ_0049271	hsa_miR_767-3p	ITGB3
hsa_circ_0049271	hsa_miR_767-3p	TRDN
hsa_circ_0049271	hsa_miR_767-3p	NKD1
hsa_circ_0049271	hsa_miR_767-3p	FOXI1
hsa_circ_0049271	hsa_miR_767-3p	CRIM1
hsa_circ_0049271	hsa_miR_767-3p	SULT4A1
hsa_circ_0049271	hsa_miR_767-3p	NPR1
hsa_circ_0049271	hsa_miR_767-3p	ASF1B
hsa_circ_0049271	hsa_miR_767-3p	CENPO
hsa_circ_0049271	hsa_miR_767-3p	GUCY1A2
hsa_circ_0049271	hsa_miR_767-3p	ALG3
hsa_circ_0049271	hsa_miR_767-3p	MAGI1
hsa_circ_0049271	hsa_miR_767-3p	AJAP1
hsa_circ_0049271	hsa_miR_767-3p	DTYMK
hsa_circ_0049271	hsa_miR_767-3p	FAXC
hsa_circ_0049271	hsa_miR_767-3p	CNGB1
hsa_circ_0049271	hsa_miR_767-3p	AGT
hsa_circ_0049271	hsa_miR_767-3p	ACE2
hsa_circ_0049271	hsa_miR_767-3p	PROX1

hsa_circ_0049271	hsa_miR_767-3p	FBXO32
hsa_circ_0049271	hsa_miR_767-3p	OLFML2A
hsa_circ_0049271	hsa_miR_767-3p	CCSER1
hsa_circ_0049271	hsa_miR_767-3p	AK4
hsa_circ_0049271	hsa_miR_767-3p	ARC
hsa_circ_0049271	hsa_miR_767-3p	PHEX
hsa_circ_0049271	hsa_miR_767-3p	KCNJ9
hsa_circ_0049271	hsa_miR_767-3p	MOCS1
hsa_circ_0049271	hsa_miR_767-3p	LGSN
hsa_circ_0049271	hsa_miR_767-3p	KNCN
hsa_circ_0049271	hsa_miR_767-3p	MDGA1
hsa_circ_0049271	hsa_miR_767-3p	SGPL1
hsa_circ_0049271	hsa_miR_767-3p	SLC13A5
hsa_circ_0049271	hsa_miR_767-3p	C5orf49
hsa_circ_0049271	hsa_miR_767-3p	VSTM2B
hsa_circ_0049271	hsa_miR_767-3p	HOXA5
hsa_circ_0049271	hsa_miR_767-3p	TIGIT
hsa_circ_0049271	hsa_miR_767-3p	ELAVL4
hsa_circ_0049271	hsa_miR_767-3p	MYRF
hsa_circ_0049271	hsa_miR_767-5p	TET1
hsa_circ_0049271	hsa_miR_767-5p	MYCN
hsa_circ_0049271	hsa_miR_767-5p	CPS1
hsa_circ_0049271	hsa_miR_767-5p	ACSM5
hsa_circ_0049271	hsa_miR_767-5p	COL3A1
hsa_circ_0049271	hsa_miR_767-5p	COL11A1
hsa_circ_0049271	hsa_miR_767-5p	KLF6
hsa_circ_0049271	hsa_miR_767-5p	KLF2
hsa_circ_0049271	hsa_miR_767-5p	PMP22
hsa_circ_0049271	hsa_miR_767-5p	PI15
hsa_circ_0049271	hsa_miR_767-5p	SMAD6
hsa_circ_0049271	hsa_miR_767-5p	DPYSL5
hsa_circ_0049271	hsa_miR_767-5p	BASP1
hsa_circ_0049271	hsa_miR_767-5p	TMEM236
hsa_circ_0049271	hsa_miR_767-5p	ADAM12
hsa_circ_0049271	hsa_miR_767-5p	FBN2
hsa_circ_0049271	hsa_miR_767-5p	COL6A3
hsa_circ_0049271	hsa_miR_767-5p	ANKRD13B
hsa_circ_0049271	hsa_miR_767-5p	SPOCK3
hsa_circ_0049271	hsa_miR_767-5p	N4BP2L1
hsa_circ_0049271	hsa_miR_767-5p	SLC16A14
hsa_circ_0049271	hsa_miR_767-5p	SOCS2
hsa_circ_0049271	hsa_miR_767-5p	GINS1
hsa_circ_0049271	hsa_miR_767-5p	GRIP1
hsa_circ_0049271	hsa_miR_767-5p	CLDN11
hsa_circ_0049271	hsa_miR_767-5p	DCC
hsa_circ_0049271	hsa_miR_767-5p	FOXE1
hsa_circ_0049271	hsa_miR_767-5p	STMN2
hsa_circ_0049271	hsa_miR_767-5p	TLL1
hsa_circ_0049271	hsa_miR_767-5p	COL4A4
hsa_circ_0049271	hsa_miR_767-5p	SCAI
hsa_circ_0049271	hsa_miR_767-5p	NCKAP5
hsa_circ_0049271	hsa_miR_767-5p	RIMS2
hsa_circ_0049271	hsa_miR_767-5p	KIF26B
hsa_circ_0049271	hsa_miR_767-5p	COL2A1
hsa_circ_0049271	hsa_miR_767-5p	TUBB2A
hsa_circ_0049271	hsa_miR_767-5p	KLF4
hsa_circ_0049271	hsa_miR_767-5p	HAPLN3
hsa_circ_0049271	hsa_miR_767-5p	FUT8

hsa_circ_0049271	hsa_miR_767-5p	TMEM88
hsa_circ_0049271	hsa_miR_767-5p	ZFHX4
hsa_circ_0049271	hsa_miR_767-5p	C11orf24
hsa_circ_0049271	hsa_miR_767-5p	SNRK
hsa_circ_0049271	hsa_miR_767-5p	KRT79
hsa_circ_0049271	hsa_miR_767-5p	COL5A2
hsa_circ_0049271	hsa_miR_767-5p	GALNT13
hsa_circ_0049271	hsa_miR_767-5p	ENPP2
hsa_circ_0049271	hsa_miR_767-5p	IDH2
hsa_circ_0049271	hsa_miR_767-5p	ISL1
hsa_circ_0049271	hsa_miR_767-5p	GPR37
hsa_circ_0049271	hsa_miR_767-5p	KCNK10
hsa_circ_0049271	hsa_miR_767-5p	DCLK1
hsa_circ_0049271	hsa_miR_767-5p	SLC35F3
hsa_circ_0049271	hsa_miR_767-5p	RTKN2
hsa_circ_0049271	hsa_miR_767-5p	SDC2
hsa_circ_0049271	hsa_miR_767-5p	KCNB1
hsa_circ_0049271	hsa_miR_767-5p	PIK3R1
hsa_circ_0049271	hsa_miR_767-5p	MEST
hsa_circ_0049271	hsa_miR_767-5p	ZPBP2
hsa_circ_0049271	hsa_miR_767-5p	SLC7A11
hsa_circ_0049271	hsa_miR_767-5p	KCNE4
hsa_circ_0049271	hsa_miR_767-5p	UNC5D
hsa_circ_0049271	hsa_miR_767-5p	RNF39
hsa_circ_0049271	hsa_miR_767-5p	SCN2B
hsa_circ_0049271	hsa_miR_767-5p	ONECUT2
hsa_circ_0049271	hsa_miR_767-5p	TRIM71
hsa_circ_0049271	hsa_miR_767-5p	SDK1
hsa_circ_0049271	hsa_miR_767-5p	PLGLB2
hsa_circ_0049271	hsa_miR_767-5p	TIMELESS
hsa_circ_0049271	hsa_miR_767-5p	COL1A2
hsa_circ_0049271	hsa_miR_767-5p	GRIK4
hsa_circ_0049271	hsa_miR_767-5p	COL10A1
hsa_circ_0049271	hsa_miR_767-5p	FGF4
hsa_circ_0049271	hsa_miR_767-5p	PROM2
hsa_circ_0049271	hsa_miR_767-5p	ZNF366
hsa_circ_0049271	hsa_miR_767-5p	COL7A1
hsa_circ_0049271	hsa_miR_767-5p	NR4A3
hsa_circ_0049271	hsa_miR_767-5p	GRAMD1B
hsa_circ_0049271	hsa_miR_767-5p	RIMS1
hsa_circ_0049271	hsa_miR_767-5p	HOXA10
hsa_circ_0049271	hsa_miR_767-5p	PGM5
hsa_circ_0049271	hsa_miR_767-5p	DLX5
hsa_circ_0049271	hsa_miR_767-5p	STK32B
hsa_circ_0049271	hsa_miR_767-5p	SPN
hsa_circ_0049271	hsa_miR_767-5p	PDGFB
hsa_circ_0049271	hsa_miR_767-5p	TNFAIP3
hsa_circ_0049271	hsa_miR_767-5p	PAX9
hsa_circ_0049271	hsa_miR_767-5p	ELOVL4
hsa_circ_0049271	hsa_miR_767-5p	TFAP2C
hsa_circ_0049271	hsa_miR_767-5p	TRIM16L
hsa_circ_0049271	hsa_miR_767-5p	SLC9A2
hsa_circ_0049271	hsa_miR_767-5p	ELOVL6
hsa_circ_0049271	hsa_miR_767-5p	LOXL2
hsa_circ_0049271	hsa_miR_767-5p	TENM1
hsa_circ_0049271	hsa_miR_767-5p	CYP27C1
hsa_circ_0049271	hsa_miR_767-5p	PHACTR2
hsa_circ_0049271	hsa_miR_767-5p	MEX3B

hsa_circ_0049271	hsa_miR_767-5p	HEY2
hsa_circ_0049271	hsa_miR_767-5p	CDH5
hsa_circ_0049271	hsa_miR_767-5p	CLEC4D
hsa_circ_0049271	hsa_miR_767-5p	NANOS1
hsa_circ_0049271	hsa_miR_767-5p	SYNPO2
hsa_circ_0049271	hsa_miR_767-5p	PROX1
hsa_circ_0049271	hsa_miR_767-5p	HECW1
hsa_circ_0049271	hsa_miR_767-5p	PHF21B
hsa_circ_0049271	hsa_miR_767-5p	NKD1
hsa_circ_0049271	hsa_miR_767-5p	NRG1
hsa_circ_0049271	hsa_miR_767-5p	GNAQ
hsa_circ_0049271	hsa_miR_767-5p	SGMS2
hsa_circ_0049271	hsa_miR_767-5p	TMEM212
hsa_circ_0049271	hsa_miR_767-5p	TUBB6
hsa_circ_0049271	hsa_miR_767-5p	SMCO1
hsa_circ_0049271	hsa_miR_767-5p	ZIC5
hsa_circ_0049271	hsa_miR_767-5p	SPDL1
hsa_circ_0049271	hsa_miR_767-5p	FZD4
hsa_circ_0049271	hsa_miR_767-5p	EYA4
hsa_circ_0049271	hsa_miR_767-5p	NDST1
hsa_circ_0049271	hsa_miR_767-5p	ZFP42
hsa_circ_0049271	hsa_miR_767-5p	IL11
hsa_circ_0049271	hsa_miR_767-5p	TPBG
hsa_circ_0049271	hsa_miR_767-5p	MTMR7
hsa_circ_0049271	hsa_miR_767-5p	GINS2
hsa_circ_0049271	hsa_miR_767-5p	BMPER
hsa_circ_0049271	hsa_miR_767-5p	SLC5A7
hsa_circ_0049271	hsa_miR_767-5p	SELE
hsa_circ_0049271	hsa_miR_767-5p	CCND2
hsa_circ_0049271	hsa_miR_767-5p	STRIP2
hsa_circ_0049271	hsa_miR_767-5p	SOX4
hsa_circ_0049271	hsa_miR_767-5p	SIM1
hsa_circ_0049271	hsa_miR_767-5p	XIRP2
hsa_circ_0049271	hsa_miR_767-5p	NPAS3
hsa_circ_0049271	hsa_miR_767-5p	DIXDC1
hsa_circ_0049271	hsa_miR_767-5p	IFNB1
hsa_circ_0049271	hsa_miR_767-5p	FAM135B
hsa_circ_0049271	hsa_miR_767-5p	MUC22
hsa_circ_0049271	hsa_miR_324-5p	MGAT3
hsa_circ_0049271	hsa_miR_324-5p	PCYT1B
hsa_circ_0049271	hsa_miR_324-5p	CAMKV
hsa_circ_0049271	hsa_miR_324-5p	DCX
hsa_circ_0049271	hsa_miR_324-5p	LMX1A
hsa_circ_0049271	hsa_miR_324-5p	APOLD1
hsa_circ_0049271	hsa_miR_324-5p	ARHGAP36
hsa_circ_0049271	hsa_miR_324-5p	PTPRD
hsa_circ_0049271	hsa_miR_324-5p	MMP19
hsa_circ_0049271	hsa_miR_324-5p	EYA4
hsa_circ_0049271	hsa_miR_1270	N4BP2L1
hsa_circ_0049271	hsa_miR_1270	TESC
hsa_circ_0049271	hsa_miR_1270	UBASH3B
hsa_circ_0049271	hsa_miR_1270	ZIC5
hsa_circ_0049271	hsa_miR_1270	AFP
hsa_circ_0049271	hsa_miR_1270	STOML3
hsa_circ_0049271	hsa_miR_1270	SLC24A2
hsa_circ_0049271	hsa_miR_1270	DNAAF3
hsa_circ_0049271	hsa_miR_1270	XKR9
hsa_circ_0049271	hsa_miR_1270	SLITRK2

hsa_circ_0049271	hsa_miR_1270	LYSMD1
hsa_circ_0049271	hsa_miR_1270	UNC45B
hsa_circ_0049271	hsa_miR_1270	HAS1
hsa_circ_0049271	hsa_miR_1270	SPTBN1
hsa_circ_0049271	hsa_miR_1270	UNC5CL
hsa_circ_0049271	hsa_miR_1270	KCNA4
hsa_circ_0049271	hsa_miR_1270	CNDP1
hsa_circ_0049271	hsa_miR_1270	INHBB
hsa_circ_0049271	hsa_miR_1270	DENND2C
hsa_circ_0049271	hsa_miR_1270	CEACAM1
hsa_circ_0049271	hsa_miR_1270	CASP14
hsa_circ_0049271	hsa_miR_1270	CNGB3
hsa_circ_0049271	hsa_miR_1270	CNNM1
hsa_circ_0049271	hsa_miR_1270	DCX
hsa_circ_0049271	hsa_miR_1270	BAAT
hsa_circ_0049271	hsa_miR_1270	COL22A1
hsa_circ_0049271	hsa_miR_1270	CCDC74B
hsa_circ_0049271	hsa_miR_1270	F8
hsa_circ_0049271	hsa_miR_1270	ANGPTL7
hsa_circ_0049271	hsa_miR_1270	TBC1D26
hsa_circ_0049271	hsa_miR_1270	RGS7
hsa_circ_0049271	hsa_miR_1270	NOVA2
hsa_circ_0049271	hsa_miR_1270	CTSE
hsa_circ_0049271	hsa_miR_1270	DIXDC1
hsa_circ_0049271	hsa_miR_1270	ACTBL2
hsa_circ_0049271	hsa_miR_1270	NEGR1
hsa_circ_0049271	hsa_miR_1270	LHX2
hsa_circ_0049271	hsa_miR_1270	FAM189A1
hsa_circ_0049271	hsa_miR_1270	GPC5
hsa_circ_0049271	hsa_miR_1270	STEAP2
hsa_circ_0049271	hsa_miR_1270	DLX3
hsa_circ_0049271	hsa_miR_1270	LGI1
hsa_circ_0049271	hsa_miR_1270	SLC39A8
hsa_circ_0049271	hsa_miR_1270	OLFM4
hsa_circ_0049271	hsa_miR_1270	ILF2
hsa_circ_0049271	hsa_miR_1270	HOXC5
hsa_circ_0049271	hsa_miR_1270	ST8SIA6
hsa_circ_0049271	hsa_miR_1270	SHISA9
hsa_circ_0049271	hsa_miR_1270	NEUROD1
hsa_circ_0049271	hsa_miR_1270	SHISA3
hsa_circ_0049271	hsa_miR_1270	ABCA3
hsa_circ_0049271	hsa_miR_1270	C6orf15
hsa_circ_0049271	hsa_miR_1270	TMEM236
hsa_circ_0049271	hsa_miR_1270	HMGCLL1
hsa_circ_0049271	hsa_miR_1270	SPN
hsa_circ_0049271	hsa_miR_1270	PATE2
hsa_circ_0049271	hsa_miR_1270	SH2D4B
hsa_circ_0049271	hsa_miR_1270	EMCN
hsa_circ_0049271	hsa_miR_1270	SRD5A3
hsa_circ_0049271	hsa_miR_1270	SLC1A1
hsa_circ_0049271	hsa_miR_1270	GPR26
hsa_circ_0049271	hsa_miR_1270	KIAA1210
hsa_circ_0049271	hsa_miR_1270	BMP8B
hsa_circ_0049271	hsa_miR_1270	CPNE6
hsa_circ_0049271	hsa_miR_1270	MUC21
hsa_circ_0049271	hsa_miR_1270	FABP5
hsa_circ_0049271	hsa_miR_1270	IL17RD
hsa_circ_0049271	hsa_miR_1270	REG1B

hsa_circ_0049271	hsa_miR_215	EREG
hsa_circ_0049271	hsa_miR_215	NIPAL1
hsa_circ_0049271	hsa_miR_215	GPR22
hsa_circ_0049271	hsa_miR_215	NKAIN2
hsa_circ_0049271	hsa_miR_215	ZEB2
hsa_circ_0049271	hsa_miR_215	FRMD4B
hsa_circ_0049271	hsa_miR_215	OLIG3
hsa_circ_0049271	hsa_miR_215	ZNF536
hsa_circ_0049271	hsa_miR_215	MYLK
hsa_circ_0049271	hsa_miR_215	RAD54B
hsa_circ_0049271	hsa_miR_215	GALNTL6
hsa_circ_0049271	hsa_miR_215	IGDCC3
hsa_circ_0049271	hsa_miR_215	FAXC
hsa_circ_0049271	hsa_miR_215	ENC1
hsa_circ_0049271	hsa_miR_215	RUNX1T1
hsa_circ_0049271	hsa_miR_215	COL5A1
hsa_circ_0049271	hsa_miR_215	TCTEX1D1
hsa_circ_0072088	hsa_miR_532-3p	NDP
hsa_circ_0072088	hsa_miR_532-3p	CLVS2
hsa_circ_0072088	hsa_miR_532-3p	CXCL2
hsa_circ_0072088	hsa_miR_532-3p	SLC39A8
hsa_circ_0072088	hsa_miR_532-3p	LINGO2
hsa_circ_0072088	hsa_miR_532-3p	NKD1
hsa_circ_0072088	hsa_miR_532-3p	ERCC6L
hsa_circ_0072088	hsa_miR_532-3p	NSG2
hsa_circ_0072088	hsa_miR_532-3p	TRHDE
hsa_circ_0072088	hsa_miR_532-3p	CHL1
hsa_circ_0072088	hsa_miR_532-3p	CPD
hsa_circ_0072088	hsa_miR_1270	N4BP2L1
hsa_circ_0072088	hsa_miR_1270	TESC
hsa_circ_0072088	hsa_miR_1270	UBASH3B
hsa_circ_0072088	hsa_miR_1270	ZIC5
hsa_circ_0072088	hsa_miR_1270	AFP
hsa_circ_0072088	hsa_miR_1270	STOML3
hsa_circ_0072088	hsa_miR_1270	SLC24A2
hsa_circ_0072088	hsa_miR_1270	DNAAF3
hsa_circ_0072088	hsa_miR_1270	XKR9
hsa_circ_0072088	hsa_miR_1270	SLITRK2
hsa_circ_0072088	hsa_miR_1270	LYSMD1
hsa_circ_0072088	hsa_miR_1270	UNC45B
hsa_circ_0072088	hsa_miR_1270	HAS1
hsa_circ_0072088	hsa_miR_1270	SPTBN1
hsa_circ_0072088	hsa_miR_1270	UNC5CL
hsa_circ_0072088	hsa_miR_1270	KCNA4
hsa_circ_0072088	hsa_miR_1270	CNDP1
hsa_circ_0072088	hsa_miR_1270	INHBB
hsa_circ_0072088	hsa_miR_1270	DENND2C
hsa_circ_0072088	hsa_miR_1270	CEACAM1
hsa_circ_0072088	hsa_miR_1270	CASP14
hsa_circ_0072088	hsa_miR_1270	CNGB3
hsa_circ_0072088	hsa_miR_1270	CNNM1
hsa_circ_0072088	hsa_miR_1270	DCX
hsa_circ_0072088	hsa_miR_1270	BAAT
hsa_circ_0072088	hsa_miR_1270	COL22A1
hsa_circ_0072088	hsa_miR_1270	CCDC74B
hsa_circ_0072088	hsa_miR_1270	F8
hsa_circ_0072088	hsa_miR_1270	ANGPTL7
hsa_circ_0072088	hsa_miR_1270	TBC1D26

hsa_circ_0072088	hsa_miR_1270	RGS7
hsa_circ_0072088	hsa_miR_1270	NOVA2
hsa_circ_0072088	hsa_miR_1270	CTSE
hsa_circ_0072088	hsa_miR_1270	DIXDC1
hsa_circ_0072088	hsa_miR_1270	ACTBL2
hsa_circ_0072088	hsa_miR_1270	NEGR1
hsa_circ_0072088	hsa_miR_1270	LHX2
hsa_circ_0072088	hsa_miR_1270	FAM189A1
hsa_circ_0072088	hsa_miR_1270	GPC5
hsa_circ_0072088	hsa_miR_1270	STEAP2
hsa_circ_0072088	hsa_miR_1270	DLX3
hsa_circ_0072088	hsa_miR_1270	LGI1
hsa_circ_0072088	hsa_miR_1270	SLC39A8
hsa_circ_0072088	hsa_miR_1270	OLFM4
hsa_circ_0072088	hsa_miR_1270	ILF2
hsa_circ_0072088	hsa_miR_1270	HOXC5
hsa_circ_0072088	hsa_miR_1270	ST8SIA6
hsa_circ_0072088	hsa_miR_1270	SHISA9
hsa_circ_0072088	hsa_miR_1270	NEUROD1
hsa_circ_0072088	hsa_miR_1270	SHISA3
hsa_circ_0072088	hsa_miR_1270	ABCA3
hsa_circ_0072088	hsa_miR_1270	C6orf15
hsa_circ_0072088	hsa_miR_1270	TMEM236
hsa_circ_0072088	hsa_miR_1270	HMGCLL1
hsa_circ_0072088	hsa_miR_1270	SPN
hsa_circ_0072088	hsa_miR_1270	PATE2
hsa_circ_0072088	hsa_miR_1270	SH2D4B
hsa_circ_0072088	hsa_miR_1270	EMCN
hsa_circ_0072088	hsa_miR_1270	SRD5A3
hsa_circ_0072088	hsa_miR_1270	SLC1A1
hsa_circ_0072088	hsa_miR_1270	GPR26
hsa_circ_0072088	hsa_miR_1270	KIAA1210
hsa_circ_0072088	hsa_miR_1270	BMP8B
hsa_circ_0072088	hsa_miR_1270	CPNE6
hsa_circ_0072088	hsa_miR_1270	MUC21
hsa_circ_0072088	hsa_miR_1270	FABP5
hsa_circ_0072088	hsa_miR_1270	IL17RD
hsa_circ_0072088	hsa_miR_1270	REG1B
hsa_circ_0072088	hsa_miR_330-3p	GNRHR
hsa_circ_0072088	hsa_miR_330-3p	RCAN1
hsa_circ_0072088	hsa_miR_330-3p	PTGFR
hsa_circ_0072088	hsa_miR_330-3p	SOSTDC1
hsa_circ_0072088	hsa_miR_330-3p	TAC3
hsa_circ_0072088	hsa_miR_330-3p	RSPO2
hsa_circ_0072088	hsa_miR_330-3p	KLF10
hsa_circ_0072088	hsa_miR_330-3p	ADAM12
hsa_circ_0072088	hsa_miR_330-3p	CLDN18
hsa_circ_0072088	hsa_miR_330-3p	DLX1
hsa_circ_0072088	hsa_miR_330-3p	UBL3
hsa_circ_0072088	hsa_miR_330-3p	ZNF367
hsa_circ_0072088	hsa_miR_330-3p	RAI2
hsa_circ_0072088	hsa_miR_330-3p	AGTR2
hsa_circ_0072088	hsa_miR_330-3p	GPR37
hsa_circ_0072088	hsa_miR_330-3p	ZNF423
hsa_circ_0072088	hsa_miR_330-3p	PRTFDC1
hsa_circ_0072088	hsa_miR_330-3p	TXNDC17
hsa_circ_0072088	hsa_miR_330-3p	DLX6
hsa_circ_0072088	hsa_miR_330-3p	S100B

hsa_circ_0072088	hsa_miR_330-3p	C1orf115
hsa_circ_0072088	hsa_miR_330-3p	ITM2C
hsa_circ_0072088	hsa_miR_330-3p	TDRKH
hsa_circ_0072088	hsa_miR_330-3p	USP2
hsa_circ_0072088	hsa_miR_330-3p	ALAS2
hsa_circ_0072088	hsa_miR_330-3p	KLHL32
hsa_circ_0072088	hsa_miR_330-3p	NDC1
hsa_circ_0072088	hsa_miR_330-3p	MYPN
hsa_circ_0072088	hsa_miR_330-3p	SHOX2
hsa_circ_0072088	hsa_miR_330-3p	CDK1
hsa_circ_0072088	hsa_miR_330-3p	KCNC2
hsa_circ_0072088	hsa_miR_330-3p	S1PR1
hsa_circ_0072088	hsa_miR_330-3p	ONECUT2
hsa_circ_0072088	hsa_miR_330-3p	ELAVL2
hsa_circ_0072088	hsa_miR_330-3p	TBX5
hsa_circ_0072088	hsa_miR_330-3p	DTL
hsa_circ_0072088	hsa_miR_330-3p	SCG3
hsa_circ_0072088	hsa_miR_330-3p	SALL4
hsa_circ_0072088	hsa_miR_330-3p	SPRYD7
hsa_circ_0072088	hsa_miR_330-3p	FRAS1
hsa_circ_0072088	hsa_miR_330-3p	PGAM5
hsa_circ_0072088	hsa_miR_330-3p	TFAP2B
hsa_circ_0072088	hsa_miR_330-3p	SMAD7
hsa_circ_0072088	hsa_miR_330-3p	MGAM
hsa_circ_0072088	hsa_miR_330-3p	TPBG
hsa_circ_0072088	hsa_miR_330-3p	ARHGEF10
hsa_circ_0072088	hsa_miR_330-3p	JAM2
hsa_circ_0072088	hsa_miR_330-3p	SLC24A2
hsa_circ_0072088	hsa_miR_330-3p	MYRF
hsa_circ_0072088	hsa_miR_330-3p	ERBB4
hsa_circ_0072088	hsa_miR_330-3p	LAPTM5
hsa_circ_0072088	hsa_miR_330-3p	MICU3
hsa_circ_0072088	hsa_miR_330-3p	GBX2
hsa_circ_0072088	hsa_miR_330-3p	GDF6
hsa_circ_0072088	hsa_miR_330-3p	HOXC8
hsa_circ_0072088	hsa_miR_330-3p	SLC28A3
hsa_circ_0072088	hsa_miR_330-3p	GALNT7
hsa_circ_0072088	hsa_miR_330-3p	PROX1
hsa_circ_0072088	hsa_miR_330-3p	TSHR
hsa_circ_0072088	hsa_miR_330-3p	PPARGC1B
hsa_circ_0072088	hsa_miR_330-3p	COL6A5
hsa_circ_0072088	hsa_miR_330-3p	PTPRM
hsa_circ_0072088	hsa_miR_330-3p	RFWD3
hsa_circ_0072088	hsa_miR_330-3p	BFSP2
hsa_circ_0072088	hsa_miR_330-3p	DIRAS2
hsa_circ_0072088	hsa_miR_330-3p	KAT2B
hsa_circ_0072088	hsa_miR_330-3p	EGR4
hsa_circ_0072088	hsa_miR_330-3p	DRP2
hsa_circ_0072088	hsa_miR_330-3p	STXBP5L
hsa_circ_0072088	hsa_miR_330-3p	ST8SIA3
hsa_circ_0072088	hsa_miR_330-3p	KANK2
hsa_circ_0072088	hsa_miR_330-3p	NRG3
hsa_circ_0072088	hsa_miR_330-3p	LRP8
hsa_circ_0072088	hsa_miR_330-3p	ADCY9
hsa_circ_0072088	hsa_miR_330-3p	L1CAM
hsa_circ_0072088	hsa_miR_330-3p	C1orf21
hsa_circ_0072088	hsa_miR_330-3p	SEMA3A
hsa_circ_0072088	hsa_miR_330-3p	TGFBR3

hsa_circ_0072088	hsa_miR_330-3p	E2F1
hsa_circ_0072088	hsa_miR_330-3p	TNFAIP3
hsa_circ_0072088	hsa_miR_330-3p	NDNF
hsa_circ_0072088	hsa_miR_330-3p	SCN1A
hsa_circ_0072088	hsa_miR_330-3p	PCYT1B
hsa_circ_0072088	hsa_miR_330-3p	NAT8L
hsa_circ_0072088	hsa_miR_330-3p	PRKG1
hsa_circ_0072088	hsa_miR_330-3p	BMPR2
hsa_circ_0072088	hsa_miR_330-3p	NKAIN1
hsa_circ_0072088	hsa_miR_330-3p	ADCYAP1R1
hsa_circ_0072088	hsa_miR_330-3p	SYT5
hsa_circ_0072088	hsa_miR_330-3p	GJA3
hsa_circ_0072088	hsa_miR_330-3p	ADRA2A
hsa_circ_0072088	hsa_miR_330-3p	ATP2B2
hsa_circ_0072088	hsa_miR_330-3p	MYH10
hsa_circ_0072088	hsa_miR_330-3p	NPR3
hsa_circ_0072088	hsa_miR_330-3p	ANKRD33B
hsa_circ_0072088	hsa_miR_330-3p	FBLN5
hsa_circ_0072088	hsa_miR_330-3p	MAPK10
hsa_circ_0072088	hsa_miR_330-3p	JPH3
hsa_circ_0072088	hsa_miR_330-3p	CA12
hsa_circ_0072088	hsa_miR_330-3p	GPM6A
hsa_circ_0072088	hsa_miR_330-3p	PPM1H
hsa_circ_0072088	hsa_miR_330-3p	TYRP1
hsa_circ_0072088	hsa_miR_330-3p	PCDHA7
hsa_circ_0072088	hsa_miR_330-3p	PCDHA12
hsa_circ_0072088	hsa_miR_330-3p	PCDHA5
hsa_circ_0072088	hsa_miR_330-3p	PCDHA11
hsa_circ_0072088	hsa_miR_330-3p	PCDHA9
hsa_circ_0072088	hsa_miR_330-3p	PCDHA6
hsa_circ_0072088	hsa_miR_330-3p	PCDHA1
hsa_circ_0072088	hsa_miR_330-3p	PCDHA4
hsa_circ_0072088	hsa_miR_330-3p	ZBTB20
hsa_circ_0072088	hsa_miR_942	LRP8
hsa_circ_0072088	hsa_miR_942	KNG1
hsa_circ_0072088	hsa_miR_942	RANBP3L
hsa_circ_0072088	hsa_miR_942	THRB
hsa_circ_0072088	hsa_miR_942	HSPD1
hsa_circ_0072088	hsa_miR_942	CCBE1
hsa_circ_0072088	hsa_miR_942	WASF3
hsa_circ_0072088	hsa_miR_942	C1orf56
hsa_circ_0072088	hsa_miR_942	CHEK1
hsa_circ_0072088	hsa_miR_942	CACNB4
hsa_circ_0072088	hsa_miR_942	CLVS2
hsa_circ_0072088	hsa_miR_942	VGLL3
hsa_circ_0072088	hsa_miR_942	TMEM178B
hsa_circ_0072088	hsa_miR_942	RAD54B
hsa_circ_0072088	hsa_miR_942	FUT8
hsa_circ_0072088	hsa_miR_942	B3GALNT1
hsa_circ_0072088	hsa_miR_942	DEPDC4
hsa_circ_0072088	hsa_miR_942	SMKR1
hsa_circ_0072088	hsa_miR_942	TAT
hsa_circ_0072088	hsa_miR_942	KIAA1549L
hsa_circ_0072088	hsa_miR_942	P2RY12
hsa_circ_0072088	hsa_miR_942	LSAMP
hsa_circ_0072088	hsa_miR_942	CCL14
hsa_circ_0072088	hsa_miR_942	TNS1
hsa_circ_0072088	hsa_miR_942	PACRG

hsa_circ_0072088	hsa_miR_942	PPM1F
hsa_circ_0072088	hsa_miR_942	PIF1
hsa_circ_0072088	hsa_miR_942	TGFA
hsa_circ_0072088	hsa_miR_942	CACNA1E
hsa_circ_0072088	hsa_miR_942	GDNF
hsa_circ_0072088	hsa_miR_942	DDX4
hsa_circ_0072088	hsa_miR_942	CD83
hsa_circ_0072088	hsa_miR_942	GINS4
hsa_circ_0072088	hsa_miR_942	C8A
hsa_circ_0072088	hsa_miR_942	PRKG1
hsa_circ_0072088	hsa_miR_942	NCKAP5
hsa_circ_0072088	hsa_miR_942	ANKRD34B
hsa_circ_0072088	hsa_miR_942	EMP2
hsa_circ_0072088	hsa_miR_942	DCAF4L2
hsa_circ_0072088	hsa_miR_942	AVL9
hsa_circ_0072088	hsa_miR_942	RAB3B
hsa_circ_0072088	hsa_miR_942	NECAB1
hsa_circ_0072088	hsa_miR_942	KCNB1
hsa_circ_0072088	hsa_miR_942	HPRT1
hsa_circ_0072088	hsa_miR_942	PKP2
hsa_circ_0072088	hsa_miR_942	HOXA10
hsa_circ_0072088	hsa_miR_942	WDHD1
hsa_circ_0072088	hsa_miR_942	MEX3A
hsa_circ_0072088	hsa_miR_942	SLC17A2
hsa_circ_0072088	hsa_miR_942	SGPL1
hsa_circ_0072088	hsa_miR_942	LRRC15
hsa_circ_0072088	hsa_miR_942	BEST4
hsa_circ_0072088	hsa_miR_942	OPCML
hsa_circ_0072088	hsa_miR_942	OTC
hsa_circ_0072088	hsa_miR_942	CSMD2
hsa_circ_0072088	hsa_miR_942	CKAP2
hsa_circ_0072088	hsa_miR_942	NEDD9
hsa_circ_0072088	hsa_miR_942	PLCL1
hsa_circ_0072088	hsa_miR_942	PDK1
hsa_circ_0072088	hsa_miR_942	EPHA7
hsa_circ_0072088	hsa_miR_942	GFPT1
hsa_circ_0072088	hsa_miR_942	COL6A6
hsa_circ_0072088	hsa_miR_942	PDK4
hsa_circ_0072088	hsa_miR_942	HMBS
hsa_circ_0072088	hsa_miR_942	NFASC
hsa_circ_0072088	hsa_miR_942	SPDYA
hsa_circ_0072088	hsa_miR_942	ROBO4
hsa_circ_0072088	hsa_miR_942	CLDN12
hsa_circ_0072088	hsa_miR_942	HS3ST5
hsa_circ_0072088	hsa_miR_942	FGFR2
hsa_circ_0072088	hsa_miR_942	PARPBP
hsa_circ_0072088	hsa_miR_942	ZNF710
hsa_circ_0072088	hsa_miR_942	LRRC10B
hsa_circ_0072088	hsa_miR_942	GUCY1A2
hsa_circ_0072088	hsa_miR_942	PCDH17
hsa_circ_0072088	hsa_miR_942	ONECUT2
hsa_circ_0072088	hsa_miR_942	HTR4
hsa_circ_0072088	hsa_miR_942	EDARADD
hsa_circ_0072088	hsa_miR_942	PHACTR2
hsa_circ_0072088	hsa_miR_942	STARD8
hsa_circ_0072088	hsa_miR_942	FCRL4
hsa_circ_0072088	hsa_miR_942	PDE1C
hsa_circ_0072088	hsa_miR_942	OR51E1

hsa_circ_0072088	hsa_miR_942	CACNG8
hsa_circ_0072088	hsa_miR_942	NFIX
hsa_circ_0072088	hsa_miR_942	NTM
hsa_circ_0072088	hsa_miR_942	BACE2
hsa_circ_0072088	hsa_miR_942	HS3ST3A1
hsa_circ_0072088	hsa_miR_942	RASSF6
hsa_circ_0072088	hsa_miR_942	ZEB2
hsa_circ_0072088	hsa_miR_942	ARRB1
hsa_circ_0072088	hsa_miR_942	CHRDL1
hsa_circ_0072088	hsa_miR_942	LIN28B
hsa_circ_0072088	hsa_miR_942	SEMA5A
hsa_circ_0072088	hsa_miR_942	FAXC
hsa_circ_0072088	hsa_miR_942	MANEAL
hsa_circ_0072088	hsa_miR_942	MSRB3
hsa_circ_0072088	hsa_miR_942	KCNC1
hsa_circ_0072088	hsa_miR_942	GATA6
hsa_circ_0072088	hsa_miR_942	TSPAN5
hsa_circ_0072088	hsa_miR_942	SDK1
hsa_circ_0072088	hsa_miR_942	F2RL3
hsa_circ_0072088	hsa_miR_942	ETS1
hsa_circ_0072088	hsa_miR_942	TEX13B
hsa_circ_0072088	hsa_miR_942	TEK
hsa_circ_0072088	hsa_miR_942	SLC1A1
hsa_circ_0072088	hsa_miR_942	SCAI
hsa_circ_0072088	hsa_miR_942	KDM5B
hsa_circ_0072088	hsa_miR_942	C1orf115
hsa_circ_0072088	hsa_miR_942	PLA2G4F
hsa_circ_0072088	hsa_miR_942	ZNF106
hsa_circ_0072088	hsa_miR_942	MEF2B
hsa_circ_0072088	hsa_miR_942	DPP4
hsa_circ_0072088	hsa_miR_942	TMPRSS11D
hsa_circ_0072088	hsa_miR_942	SLC6A11
hsa_circ_0072088	hsa_miR_942	USP2
hsa_circ_0072088	hsa_miR_942	GRID1
hsa_circ_0072088	hsa_miR_942	ENDOU
hsa_circ_0072088	hsa_miR_942	HECW1
hsa_circ_0072088	hsa_miR_942	TLL2
hsa_circ_0072088	hsa_miR_942	CBR1
hsa_circ_0072088	hsa_miR_942	EIF4E3
hsa_circ_0072088	hsa_miR_942	CCL25
hsa_circ_0072088	hsa_miR_942	KRT4
hsa_circ_0072088	hsa_miR_942	CNGB1
hsa_circ_0072088	hsa_miR_942	ZNF117
hsa_circ_0072088	hsa_miR_942	ASCL1
hsa_circ_0072088	hsa_miR_942	NR2E1
hsa_circ_0072088	hsa_miR_942	SLC24A2
hsa_circ_0072088	hsa_miR_942	STOML3
hsa_circ_0072088	hsa_miR_942	COL11A2
hsa_circ_0072088	hsa_miR_942	RNF157
hsa_circ_0072088	hsa_miR_942	SECISBP2L
hsa_circ_0072088	hsa_miR_942	RDH10
hsa_circ_0072088	hsa_miR_942	PAPPA
hsa_circ_0072088	hsa_miR_942	NMNAT2
hsa_circ_0072088	hsa_miR_942	CLEC6A
hsa_circ_0072088	hsa_miR_942	PPP2R2C
hsa_circ_0072088	hsa_miR_942	PUS7
hsa_circ_0072088	hsa_miR_942	GPR26
hsa_circ_0072088	hsa_miR_942	KANK4

hsa_circ_0072088	hsa_miR_942	HK3
hsa_circ_0072088	hsa_miR_942	GPR158
hsa_circ_0072088	hsa_miR_942	SLIT3
hsa_circ_0072088	hsa_miR_942	LPGAT1
hsa_circ_0072088	hsa_miR_942	SLC5A5
hsa_circ_0072088	hsa_miR_942	EGLN3
hsa_circ_0072088	hsa_miR_942	LRRC20
hsa_circ_0072088	hsa_miR_942	THBD
hsa_circ_0072088	hsa_miR_942	IL7R
hsa_circ_0072088	hsa_miR_942	MAPK10
hsa_circ_0072088	hsa_miR_942	SH3BP5
hsa_circ_0072088	hsa_miR_942	OTOGL
hsa_circ_0072088	hsa_miR_942	BOLA1
hsa_circ_0072088	hsa_miR_942	PAX7
hsa_circ_0072088	hsa_miR_942	NPR3
hsa_circ_0072088	hsa_miR_942	RNF43
hsa_circ_0072088	hsa_miR_942	TOM1L2
hsa_circ_0072088	hsa_miR_942	ZNF217
hsa_circ_0072088	hsa_miR_942	RNFT2
hsa_circ_0072088	hsa_miR_942	CBS
hsa_circ_0072088	hsa_miR_942	CPLX1
hsa_circ_0072088	hsa_miR_942	PRLR
hsa_circ_0072088	hsa_miR_942	BAMBI
hsa_circ_0072088	hsa_miR_942	SYNPO2
hsa_circ_0072088	hsa_miR_942	TMEM63C
hsa_circ_0072088	hsa_miR_942	CCL16
hsa_circ_0072088	hsa_miR_942	UNC5D
hsa_circ_0072088	hsa_miR_942	DCAF8L1
hsa_circ_0072088	hsa_miR_942	ALX4
hsa_circ_0072088	hsa_miR_942	DCX
hsa_circ_0072088	hsa_miR_942	MAPK8IP3
hsa_circ_0072088	hsa_miR_942	SLC8A3
hsa_circ_0072088	hsa_miR_942	PDPN
hsa_circ_0072088	hsa_miR_942	NRN1
hsa_circ_0072088	hsa_miR_942	OTX2
hsa_circ_0072088	hsa_miR_942	ZNF536
hsa_circ_0072088	hsa_miR_942	NPNT
hsa_circ_0072088	hsa_miR_942	FAM71F1
hsa_circ_0072088	hsa_miR_942	TSLP
hsa_circ_0072088	hsa_miR_942	LGSN
hsa_circ_0072088	hsa_miR_942	NPAS2
hsa_circ_0072088	hsa_miR_942	SLC11A1
hsa_circ_0072088	hsa_miR_942	PI15
hsa_circ_0072088	hsa_miR_942	A2ML1
hsa_circ_0072088	hsa_miR_942	ERG
hsa_circ_0072088	hsa_miR_942	STXBP5L
hsa_circ_0072088	hsa_miR_942	DGKI
hsa_circ_0072088	hsa_miR_942	GREM1
hsa_circ_0072088	hsa_miR_942	AMOTL1
hsa_circ_0072088	hsa_miR_942	VGLL2
hsa_circ_0072088	hsa_miR_942	UGT3A1
hsa_circ_0072088	hsa_miR_942	CRY2
hsa_circ_0072088	hsa_miR_942	TNXB
hsa_circ_0072088	hsa_miR_942	A1CF
hsa_circ_0072088	hsa_miR_942	MEOX1
hsa_circ_0072088	hsa_miR_942	MRAS
hsa_circ_0072088	hsa_miR_942	SLCO1A2
hsa_circ_0072088	hsa_miR_942	TMEM92

hsa_circ_0072088	hsa_miR_942	NEUROD1
hsa_circ_0072088	hsa_miR_942	APLN
hsa_circ_0072088	hsa_miR_942	LDLR
hsa_circ_0072088	hsa_miR_942	ERBB4
hsa_circ_0072088	hsa_miR_942	GPX8
hsa_circ_0072088	hsa_miR_942	LRRN3
hsa_circ_0072088	hsa_miR_942	ACADL
hsa_circ_0072088	hsa_miR_942	KCNA4
hsa_circ_0072088	hsa_miR_942	CEACAM16
hsa_circ_0072088	hsa_miR_942	KCNJ10
hsa_circ_0072088	hsa_miR_942	RIC3
hsa_circ_0072088	hsa_miR_942	SPOCK2
hsa_circ_0072088	hsa_miR_942	ZNF366
hsa_circ_0072088	hsa_miR_942	OTUD1
hsa_circ_0072088	hsa_miR_942	PIK3R1
hsa_circ_0072088	hsa_miR_942	SYT7
hsa_circ_0072088	hsa_miR_942	TLL1
hsa_circ_0072088	hsa_miR_942	TRAF4
hsa_circ_0072088	hsa_miR_942	VASH1
hsa_circ_0072088	hsa_miR_942	PLEKHA8
hsa_circ_0072088	hsa_miR_942	HBEGF
hsa_circ_0072088	hsa_miR_942	MDGA2
hsa_circ_0072088	hsa_miR_942	HOXC13
hsa_circ_0072088	hsa_miR_942	SLC9A7
hsa_circ_0072088	hsa_miR_942	GFRA1
hsa_circ_0072088	hsa_miR_942	LRRC18
hsa_circ_0072088	hsa_miR_942	TPPP
hsa_circ_0072088	hsa_miR_942	ZNF322
hsa_circ_0072088	hsa_miR_942	SLC22A18AS
hsa_circ_0072088	hsa_miR_942	ZC3H12B
hsa_circ_0072088	hsa_miR_942	MAGEL2
hsa_circ_0072088	hsa_miR_942	LMOD1
hsa_circ_0072088	hsa_miR_942	PKP1
