# Supplementary figures and images for: Identification of Differentially Expressed Circular RNAs as miRNA Sponges in Lung Adenocarcinoma
Source: J Oncol. 2021 Sep 10;2021:5193913. doi: 10.1155/2021/5193913 (PMC8448594; doi:10.1155/2021/5193913)

**A**

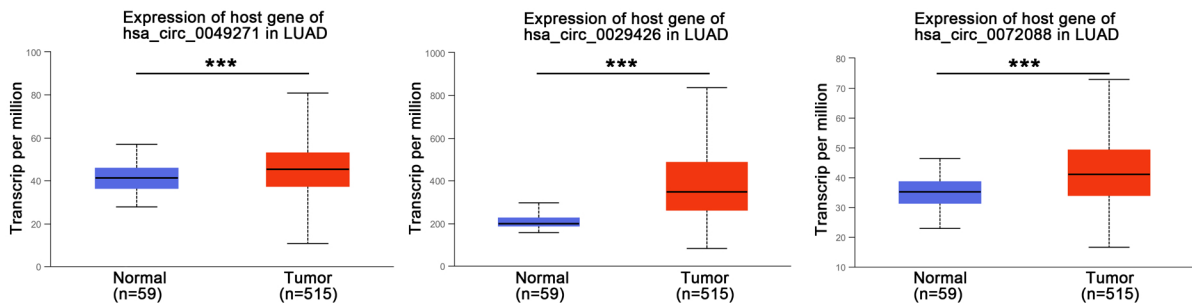

**B**

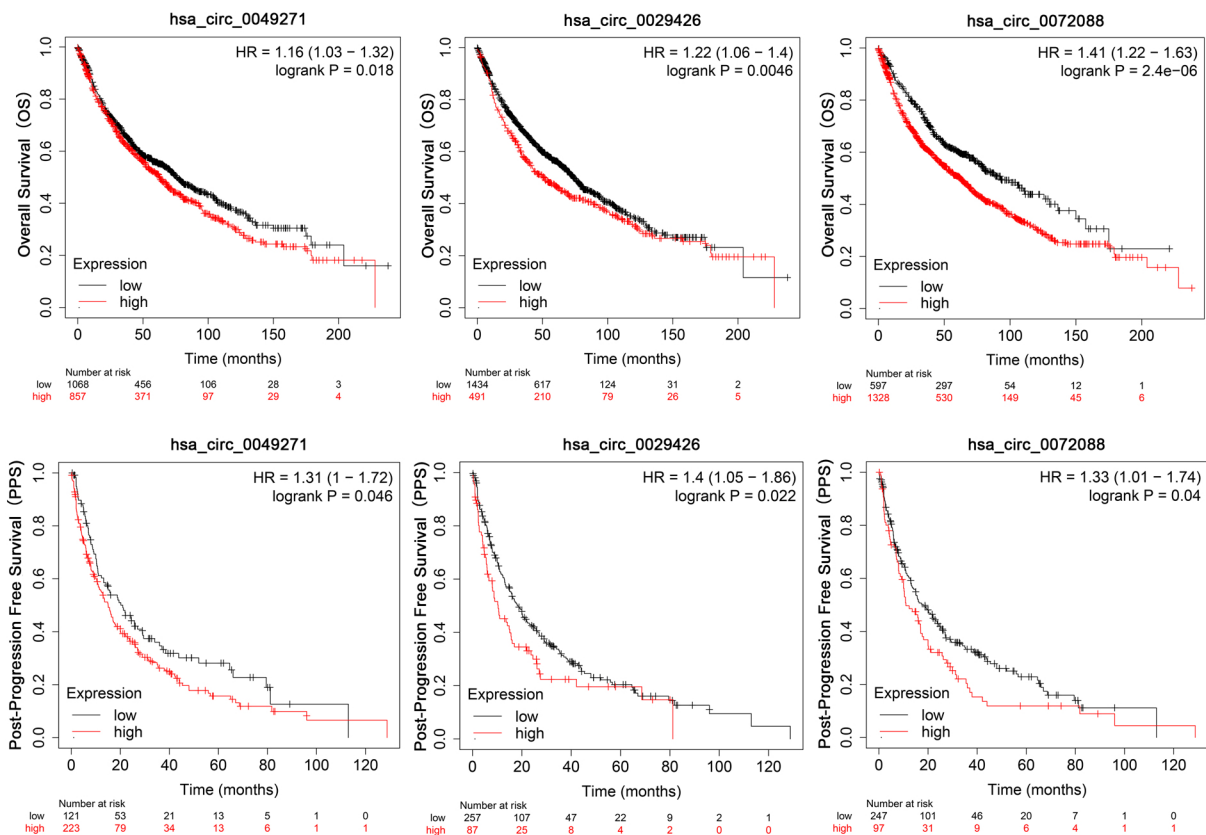

Supplement: Supplementary Materials — Supplementary Figure S1: the diagnostic and prognostic significance of the host gene. (a) The expression of the host genes of hsa_circ_0049271, hsa_circ_0029426, and hsa_circ_0072088 in The Cancer Genome Atlas (TCGA)-LUAD dataset. (b) Survival analysis for the host genes of the three circRNAs in LUAD patients was performed using the Kaplan–Meier plotter. ∗P < 0.05; ∗∗P < 0.01; ∗∗∗P < 0.001. Supplementary Figure S2: (a) GO enrichment analysis and (b) KEGG pathway enrichment analysis of DEmRNAs of hsa_circ_0003162, hsa_circ_0003528, hsa_circ_0008274, and hsa_circ_0043256. Supplementary Table 1: the construction of a circRNA-miRNA-mRNA regulatory network. [file 5193913.f1.zip › 5193913.f1/Supplementary Figure S1..pdf]

A

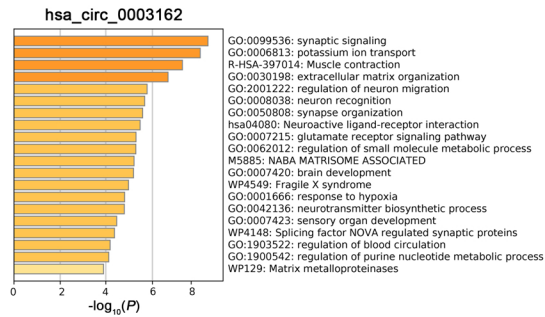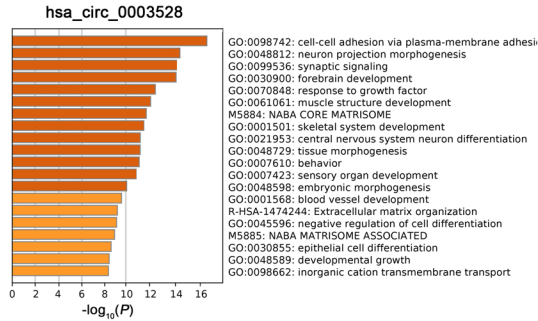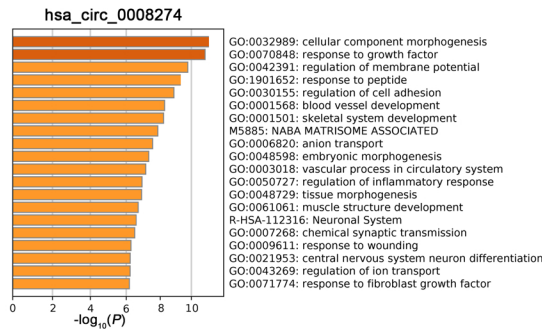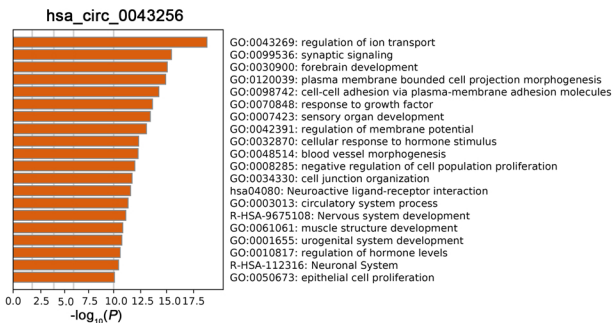

B

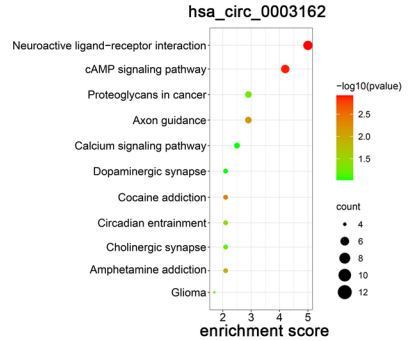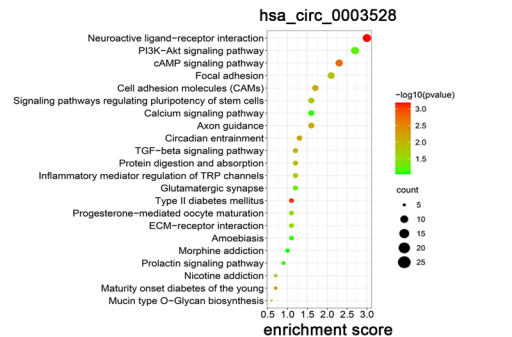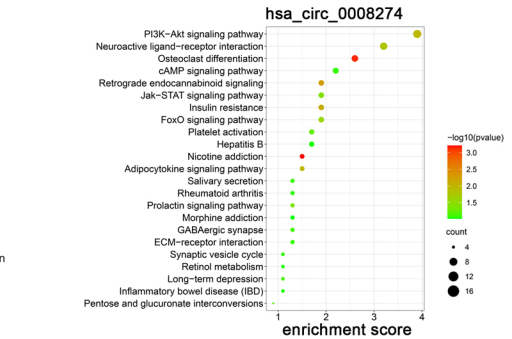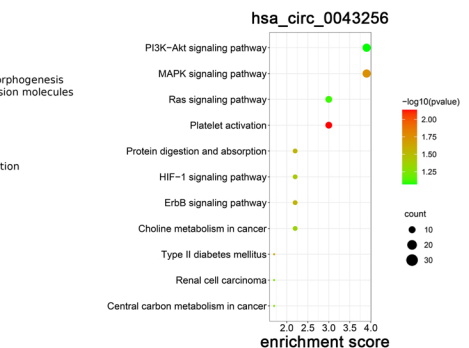

Supplement: Supplementary Materials — Supplementary Figure S1: the diagnostic and prognostic significance of the host gene. (a) The expression of the host genes of hsa_circ_0049271, hsa_circ_0029426, and hsa_circ_0072088 in The Cancer Genome Atlas (TCGA)-LUAD dataset. (b) Survival analysis for the host genes of the three circRNAs in LUAD patients was performed using the Kaplan–Meier plotter. ∗P < 0.05; ∗∗P < 0.01; ∗∗∗P < 0.001. Supplementary Figure S2: (a) GO enrichment analysis and (b) KEGG pathway enrichment analysis of DEmRNAs of hsa_circ_0003162, hsa_circ_0003528, hsa_circ_0008274, and hsa_circ_0043256. Supplementary Table 1: the construction of a circRNA-miRNA-mRNA regulatory network. [file 5193913.f1.zip › 5193913.f1/Supplementary Figure S2..pdf]
